# Stochastic transcription in the p53‐mediated response to DNA damage is modulated by burst frequency

**DOI:** 10.15252/msb.20199068

**Published:** 2019-12-02

**Authors:** Dhana Friedrich, Laura Friedel, Ana Finzel, Andreas Herrmann, Stephan Preibisch, Alexander Loewer

**Affiliations:** ^1^ Department for Biology Technische Universität Darmstadt Darmstadt Germany; ^2^ Berlin Institute for Medical Systems Biology Max Delbrück Center in the Helmholtz Association Berlin Germany; ^3^ Department for Biology Humboldt Universität zu Berlin Berlin Germany; ^4^ Janelia Research Campus Howard Hughes Medical Institute VA Ashburn USA

**Keywords:** cellular heterogeneity, DNA damage, p53 signaling, single‐cell analysis, stochastic transcription, Chromatin, Epigenetics, Genomics & Functional Genomics

## Abstract

Discontinuous transcription has been described for different mammalian cell lines and numerous promoters. However, our knowledge of how the activity of individual promoters is adjusted by dynamic signaling inputs from transcription factors is limited. To address this question, we characterized the activity of selected target genes that are regulated by pulsatile accumulation of the tumor suppressor p53 in response to ionizing radiation. We performed time‐resolved measurements of gene expression at the single‐cell level by smFISH and used the resulting data to inform a mathematical model of promoter activity. We found that p53 target promoters are regulated by frequency modulation of stochastic bursting and can be grouped along three archetypes of gene expression. The occurrence of these archetypes cannot solely be explained by nuclear p53 abundance or promoter binding of total p53. Instead, we provide evidence that the time‐varying acetylation state of p53's C‐terminal lysine residues is critical for gene‐specific regulation of stochastic bursting.

## Abstract 2

Time‐resolved gene expression measurements show that p53 induction leads to increased burst frequencies at target promoters. The promoters can be grouped into “sustained”, “pulsatile” and “transient”, and these patterns are sensitive to p53 dynamics and C‐terminal acetylation state.

## Graphical abstract

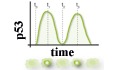

## Introduction

Cells constantly respond and adapt to extrinsic and intrinsic stimuli to mediate appropriate cell fate decisions. Intracellular signaling pathways connect these incoming signals to cellular responses through changes in abundance, localization, or post‐translational modification of signaling molecules. Recent studies employing time‐resolved single‐cell measurements highlighted that stimulus‐specific temporal activity patterns contribute to regulating gene expression and cellular phenotypes as well (Nelson *et al*, 2004; Tay *et al*, 2010; Batchelor *et al*, 2011; Hao & O'Shea, 2011; Purvis *et al*, 2012). Associated transcription factors (TF) often show pulsatile dynamics with time scales ranging from seconds (NFAT4) and minutes (NF‐κB, Msn2, Erk) to hours (p53) (Lahav *et al*, 2004; Shankaran *et al*, 2009; Tay *et al*, 2010; Hao & O'Shea, 2011; Yissachar *et al*, 2013). However, it still remains unclear how molecular circuits convert information from pulsatile TF dynamics to distinguishable expression profiles and how pulses of TFs quantitatively control transcription rates of target genes at individual promoters.

**Figure 1:**
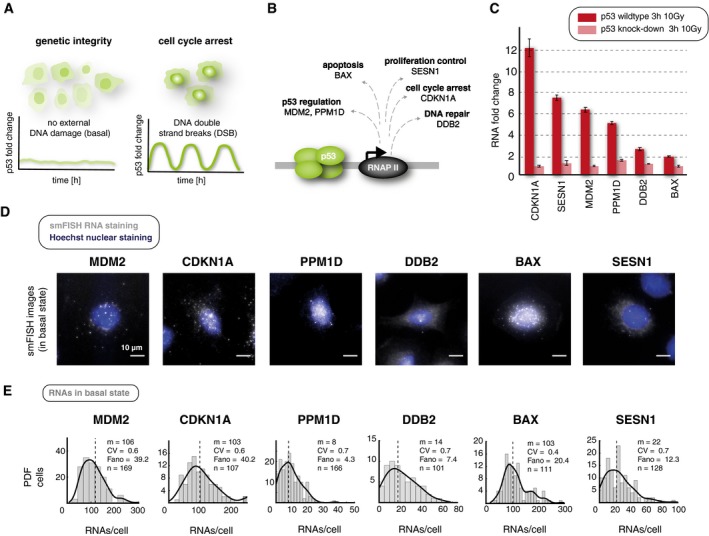
Single‐cell quantification of RNA expression by smFISH highlights strong heterogeneity of p53 target gene expression p53 has been shown to response with a series of undamped pulse to ionizing irradiation leading to cell cycle arrest while intrinsic DNA damage during cell cycle does not induce regular pulsatile p53 and subsequent gene expression programs. Schematic representations of p53 dynamics in both cellular conditions are shown.We selected p53 target genes that are involved in different cell fate programs ranging from apoptosis (BAX), DNA repair (DDB2) cell cycle arrest (CDKN1A), proliferation control (SESN1), and the regulation of the p53 network itself (PPM1D and MDM2).Induction of selected p53 target genes after DNA damage induction in A549 wild‐type and p53 knockdown cells. RNA levels were measured by qRT–PCR before and 3 h after treatment with 10 Gy IR. Fold changes relative to basal levels are shown for each cell line as mean and standard deviation from technical triplicates.Fluorescence microscopy images of smFISH probes labeled with CAL Fluor 610 (gray) overlayed with Hoechst 33342 stainings (blue) for the indicated target genes in untreated A549 cells. Scale bar corresponds to 10 μm distance; images were contrast‐ and brightness‐enhanced for better visualization.Histograms of quantitative analysis of RNAs per cell for each target gene in the absence of DNA damage (basal). smFISH staining and quantitative analysis of p53 targets show broad variability of RNA counts per cell for all genes in basal conditions. Dashed line: median; solid line: probability density estimate (see Data visualization section), CV: coefficient of variation, Fano: Fano factor, m: median, *n*: number of cells analyzed. Source data are available online for this figure.

To address these questions, we focused on the tumor suppressor p53. Its main function is to protect genetic integrity and inhibit uncontrolled proliferation in the context of cellular stress and transformation. In unstressed cells, p53 nuclear abundance is kept low through ubiquitination by the ubiquitin ligase MDM2 and rapid proteasomal degradation (Haupt *et al*, 1997; Kubbutat *et al*, 1997). In response to ionizing radiation (IR)‐induced DNA double‐strand breaks (DSBs), p53 accumulates in a series of undamped pulses (Lahav *et al*, 2004; Batchelor *et al*, 2008) (Fig 1A). In contrast, other insults such as UV radiation or chemotherapeutic drugs lead to sustained accumulation of the TF (Batchelor *et al*, 2011; Paek *et al*, 2016). P53 dynamics contribute to determining cellular outcomes, as pulsatile p53 accumulation is correlated with transient cell fate programs (cell cycle arrest) in a dose‐dependent manner, while sustained p53 levels induce terminal responses (apoptosis, senescence) (Purvis *et al*, 2012). To enable stimulus‐dependent regulation of cellular phenotypes, p53 activates the concerted transcription of target genes related to apoptosis, cell cycle arrest, DNA repair, or senescence. It has been shown that p53 activation leads to the expression of over 300 directly targeted protein‐coding genes and noncoding RNAs (Fischer, 2017). However, for many targets, the quantitative relation between p53 levels and transcriptional output has not been described. Moreover, p53 has also been detected at target sites in absence of DNA damage despite low nuclear abundance (Nikulenkov *et al*, 2012; Younger & Rinn, 2017).

According to the affinity model, the susceptibility of a target gene promoter to p53‐dependent gene expression is defined by the sequence of the corresponding p53 response element (RE). In this model, genes inducing transient phenotypes such as cell cycle arrest tend to have higher affinity for p53 binding compared to genes inducing terminal cell fates such as apoptosis (Qian *et al*, 2002; Weinberg *et al*, 2005; Murray‐Zmijewski *et al*, 2008; Kracikova *et al*, 2013). P53 REs consist of two decamers that can be separated by short spacers. Binding site affinity is primarily defined by the central conserved core motif CWWG and the length of the spacer (Riley *et al*, 2008; Verfaillie *et al*, 2016). At promoters, p53 has been shown to be involved in a set of key regulatory mechanisms, including recruitment of histone variants, histone methyltransferases, histone acetyltransferases, and components of the pre‐initiation complex (PIC) (Samuels‐Lev *et al*, 2001; Flores *et al*, 2002; Murray‐Zmijewski *et al*, 2008). Surprisingly, similar p53 levels can lead to differential locus‐ and stimulus‐specific PIC assembly. Recent live‐cell measurements of transcription at the CDKN1A promoter suggested that C‐terminal acetylation state instead of p53 abundance is the primary driving factor of transcriptional activation (Loffreda *et al*, 2017). Even though these mechanisms have been studied in biochemical assays for a selection of p53 targets, our mechanistic understanding of p53's regulatory role at promoter sites in single cells remains ambiguous at best. Mechanistic studies to date neither include temporal changes in p53 nuclear abundance, nor compare transcriptional activity at individual promoters for more than one target gene. Therefore, our current understanding on how damage‐induced dynamics of p53 are decoded on the level of gene expression remains limited.

In this study, we aimed to quantitatively measure p53‐dependent target gene expression at individual promoters in single cells. We chose a set of well‐known p53 target genes that represent different cellular response mechanisms as a paradigm and quantified corresponding nascent and matured RNA molecules by single‐molecule fluorescence *in situ* hybridization (smFISH). With the resulting quantitative data, we informed a mathematical model of promoter activity (Bahar Halpern *et al*, 2015b), which allowed us to extract transcription parameters with single‐cell and single‐molecule resolution. Using this approach, we provide a quantitative analysis of stochastic p53‐dependent gene expression at defined time points during the DNA damage response to IR induced DSBs and reveal archetypes of p53‐mediated expression dynamics. We modulated p53 dynamics using small molecule inhibitors and measured the contribution of its nuclear abundance on promoter activity. Using this approach, we found that acetylation in p53's C‐terminal lysine residues is substantially affecting stochastic transcription of target gene promoters.

## Results

### Single‐molecule mRNA quantification reveals heterogeneous expression of p53 target genes upon DNA damage with distinct abundance patterns

**Figure EV1:**
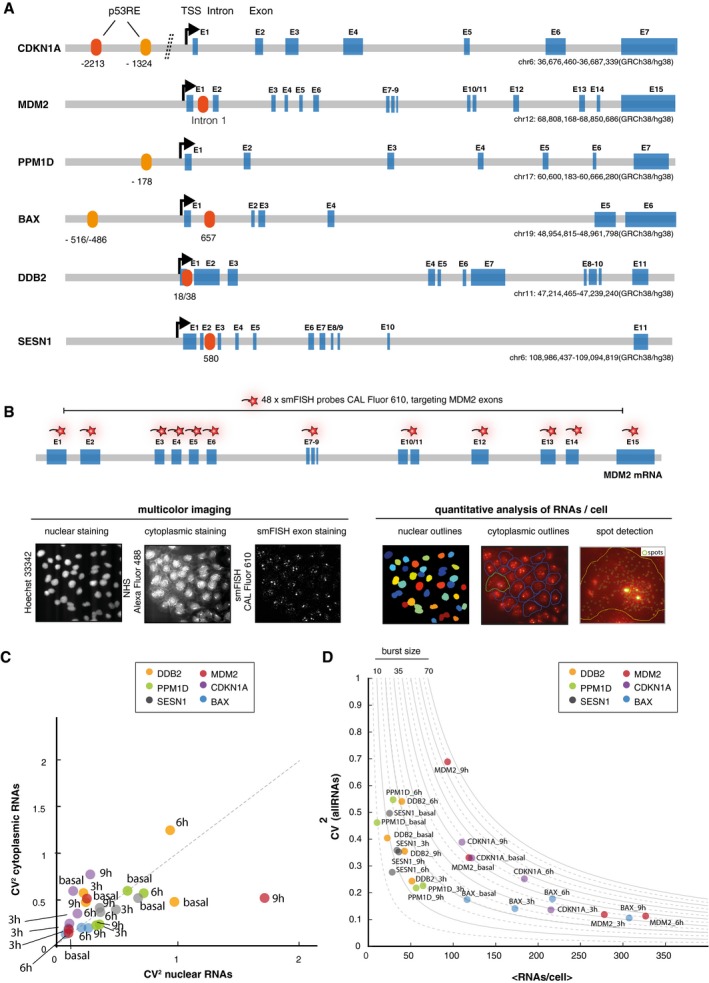
Noise in RNA abundance indicates stochastic bursting and a change in burst frequency after DNA damage p53 target genes have different genomic architecture and *cis*‐regulatory logic. The number and position of p53 response element vary dependent on the target gene. A schematic illustration shows the relative positioning of p53 response elements (RE), transcriptional start sites (TSS), and exons (Tebaldi *et al*, 2015; Fischer, 2017). p53 REs analyzed by ChIP are marked in red. Gene length and chromosomal positions are indicated. Please note that introns and exons are not drawn to scale. Gene structure represents sum of all alternative transcripts.Scheme of smFISH probe design and strategy. Multicolor fluorescent imaging enables segmentation of subcellular regions based on nuclear and cytoplasmic stains that can then be used to assign RNA counts to their cells of origin and subcellular locations. From left to right: Individual images of nuclei by Hoechst‐405, cytoplasmic staining by NHS‐488 and smFISH exon staining by Cal Fluor 610 nm staining. Examples of outline generation for nuclear and cytoplasmic regions and spot detection on exon stainings. See Materials and Methods for details.The total cytoplasmic expression noise was plotted against the total nuclear expression noise (each measured as σ^2^/μ^2 ^=^ ^CV^2^) for the indicated p53 target genes before and 3, 6, and 9 h after 10 Gy IR. Data points are labeled; time points are visualized by the indicated color code. The diagonal is shown as a guide to the eye (dashed line).The squared coefficient of variation (CV^2^) in relation to mean RNA count per cell is shown for the indicated p53 target genes over time after DNA damage (10 Gy IR). Data points are labeled with target gene and time points after IR. As guides to the eye, hyperbolic lines show the expected noise scaling upon regulating the frequency of promoter activation (CV^2 ^= *b*/<mRNA> with *b* = μ/*k*_on_ (burst size, assuming an overall low frequency of promotor activity (*k*_off_ ≫ *k*_on_), see Fig 3A and B for an illustration of the underlying model). Noise scaling for burst sizes ranging from 5 to 70 is shown as indicated on top of the graph (Singh *et al*, 2010; Dar *et al*, 2016).

To characterize how p53 pulses in response to DNA damage affect transcriptional activity at individual promoters in single cells over time, we selected a set of well‐characterized p53 targets involved in different cell fate programs (Fig 1B). The selected genes vary in *cis*‐regulatory architecture, position, and sequence of p53 REs (Fig EV1A), but show expression changes in the same order of magnitude after IR in population studies by qRT–PCR and RNA‐seq (Fig 1C and Appendix Fig S1A–C). To quantify p53‐dependent transcription at individual promoters, we performed smFISH (Bertrand *et al*, 1998; Raj *et al*, 2008) in the small cell lung carcinoma cell line A549, which shows characteristic pulses of p53 in response to IR (Finzel *et al*, 2016; Stewart‐Ornstein & Lahav, 2017) (Appendix Fig S2A and B). We assigned mRNAs to their cells of origin, using simultaneous nuclear and cytoplasmic staining and enumerated mRNAs at their subcellular localization using custom analysis scripts in combination with FISH‐quant (Fig EV1B) (Carpenter *et al*, 2006; Mueller *et al*, 2013) (see Materials and Methods section). To determine required sample sizes, we analyzed the reproducibility of our quantitative data on biological replicates of MDM2 datasets (Appendix Fig S3).

Surprisingly, our analysis showed that all selected targets were transcribed with considerable RNA counts in absence of DNA damage (Fig 1D and E, Appendix Fig S4, Dataset EV1). Basal mRNA levels varied from a few molecules to several hundreds, which is consistent with RNA‐seq data from MCF7 and MCF10A cells (Appendix Fig S1D). For all target genes, we also observed heterogeneity between individual cells (Fig 1E).

To analyze how RNA counts, localization, and variability evolve during the pulsatile p53 response in individual cells, we measured target gene mRNAs in single cells at selected time points after IR covering p53‐dependent activation of transcription, its adaptation, and progression after re‐initiation by upstream kinases (Lahav *et al*, 2004; Batchelor *et al*, 2008). In A549 cells, these time points correspond to basal (undamaged), 3 h post‐10 Gy (1^st^ p53 peak), 6 h post‐10 Gy (minimum after 1^st^ p53 pulse), and 9 h post‐10 Gy (2^nd^ p53 peak; Appendix Fig S2B). To validate pulsatile p53 level in A549 *wild‐type* cells, we performed quantitative measurements based on immunofluorescence staining (Appendix Fig S2C). Although an increase in the heterogeneity of p53 dynamics from the first to the second pulse was detected, our measurements indicate sufficient synchrony in A549 cells until 9 h after 10 Gy IR.

**Figure 2:**
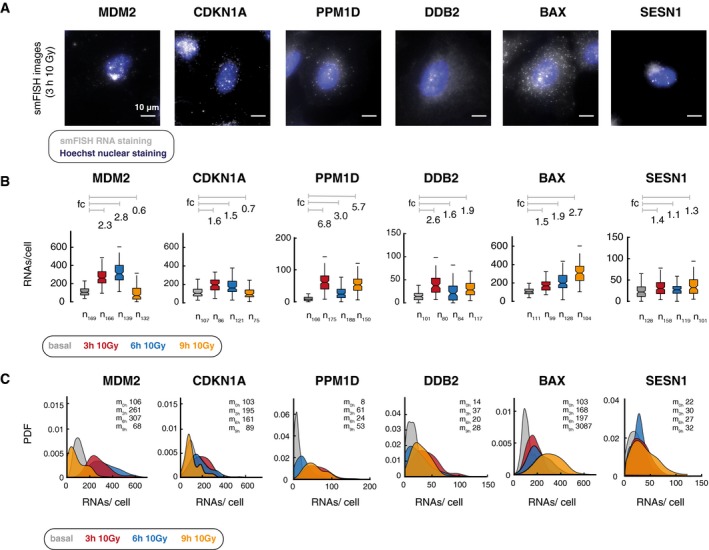
Single‐cell quantification of p53‐dependent transcription highlights distinct patterns of gene expression upon DNA damage Fluorescence microscopy images of smFISH probes CAL Fluor 610 (gray) overlayed with Hoechst 33342 staining (blue) at 3 h after 10 Gy IR or the indicated target genes in A549 cells. Scale bar corresponds to 10 μm distance; images were contrast and brightness enhanced for better visualization.We quantified RNAs per cell for the indicated target genes before (basal, gray) and 3 h (red), 6 h (blue) and 9 h (orange) after DNA damage (10 Gy IR). smFISH‐based single‐cell analysis of gene expression patterns highlights distinct RNA counts for p53 targets. RNA counts per cell are displayed as boxplots (see Data visualization section); lines indicate medians of distributions; boxes include data between the 25^th^ and 75^th^ percentiles; whiskers extend to maximum values within 1.5× the interquartile range. Notches represent 5% confidence intervals for the median. *n*: number of analyzed cells, fc: median fold of induction relative to time point basal (indicated by gray lines)Distributions of RNAs per cell for the indicated target genes before (basal, gray) and 3 h (red), 6 h (blue), and 9 h (orange) after DNA damage (10 Gy IR). Despite a clear change in median levels (m: median), single‐cell analysis reveals a strong dispersion that overlaps for the different conditions, as shown by the strongly overlapping distributions of RNA counts per cells. For better visualization, probability density estimates (PDF) based on a normal kernel are shown (see Data visualization section, compare Appendix Fig S4, raw measurements available as figure source data). Source data are available online for this figure.

In agreement with previous work, our smFISH‐based analysis showed that p53 target genes were expressed in different patterns over time with similar mean induction (fc) during the first p53 pulse for most target genes except PPM1D and gene‐specific changes at later time points (Fig 2A and B, Appendix Fig S4). The gene induction measured by smFISH was comparable with induction rates measured by RNA‐seq in MCF7 and MCF10A cells despite cell‐type‐specific differences (Appendix Fig S1D) (Porter *et al*, 2016; Hafner *et al*, 2017; Hanson *et al*, 2019). We also measured changes in the distribution of mRNA counts for each individual target when DNA damage is applied (Fig 2C) and observed gene‐specific shifts in the variability of RNA counts, indicating mechanistic changes in p53 dependent transcription upon DNA damage.

Recent literature suggested a correlation of cell cycle state and cellular volume with mRNA expression levels in single cells as well as passive buffering of expression heterogeneity through compartmentalization by limiting nuclear export (Bahar Halpern *et al*, 2015a; Battich *et al*, 2015; Padovan‐Merhar *et al*, 2015; Stoeger *et al*, 2016). Therefore, we determined total noise (measured as σ^2^/μ^2 ^= CV^2^) in the nucleus and the cytoplasm (CV^2^_nuc_/CV^2^_cyt_) for our gene set (Fig EV1C) as well as noise strength (measured as σ^2^/μ, equivalent to the Fano factor), which takes changes in mean RNA levels in cytoplasm and nucleus into account (Appendix Fig S5) (Zoller *et al*, 2015). In general, we observed comparable levels of total noise in both compartments. In condition where total noise in the nucleus was larger than in the cytoplasm, for example, for Mdm2 9 h post‐irradiation, this deviation could be explained by differences in mean RNA levels (compare Fig EV1C and S5B). In conclusion, we rather observed a trend toward noise amplification in cytoplasmic compared to nuclear fractions instead of attenuation when comparing noise strength, which is in agreement with recent work by Hansen *et al* (2018). Such an increase in noise strength might be introduced by RNA translation and degradation processes (Hansen *et al*, 2018; Baudrimont *et al*, 2019). We also only observed a minor contribution of cell cycle and volume to heterogeneity (as measured by coefficient of variation, CV; Appendix Fig S6).

### Single‐cell characterization of promoter states shows frequency modulation of bursty transcription

**Figure 3:**
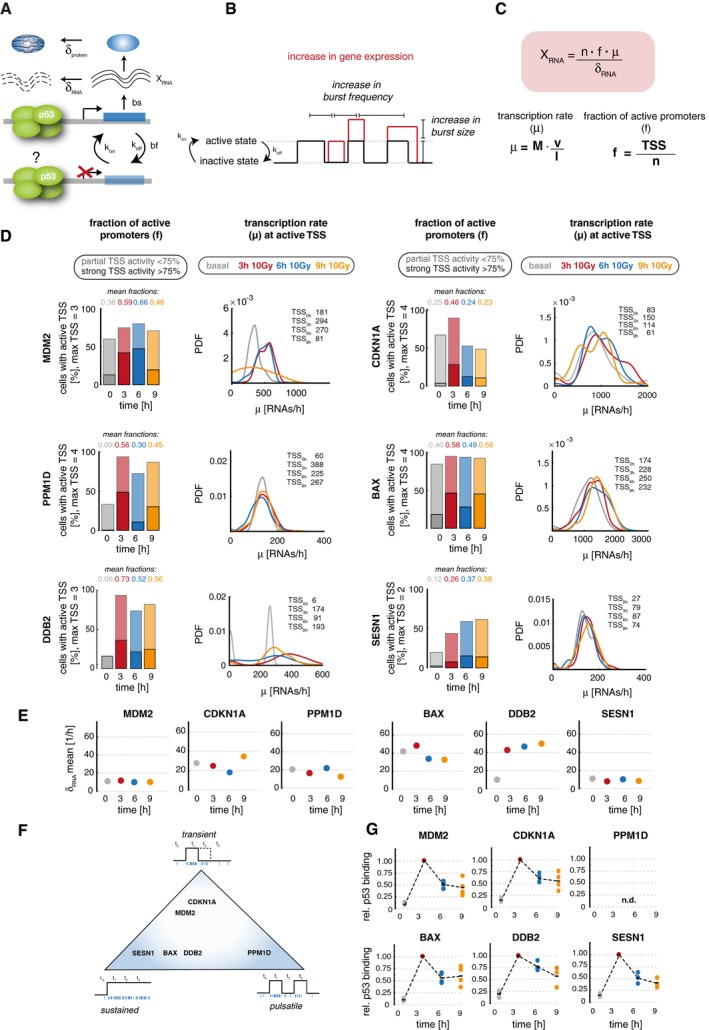
SmFISH‐based analysis at the first and second p53 pulse after IR reveals gene‐specific stochastic expression patterns Schematic illustration of the life cycle of an mRNA and the rate constants that influence RNA abundance due to stochastic bursting according to previously published models of promoter activity. While burst frequency (bf) describes the switching of a promoter between a transcriptionally active and inactive state with the rate constants *k*_on_ and *k*_off,_ the burst size (bs) describes the number of RNAs transcribed in an active period. Additionally, degradation (δ) further influences RNA levels by reducing the cytoplasmic RNA pool.Illustration of promoter activity according to the *random telegraph* model. An increase in RNA levels per cell can be due to a higher burst frequency (more active promoter periods, a higher rate of transcription initiation), or an increase in burst size (a higher rate of RNA transcription in an active period). Additionally, also mixtures of both scenarios are possible.We used smFISH data to calculated promoter activity based on previously published models. An overview of the calculations characterizing stochastic gene expression is shown. *X*_RNA_: number of quantified RNAs/cell, *n*: number of genomic loci, *f*: fraction of active promoters (proxy for burst frequency bf), μ: transcription rate per cell [RNA/h] (proxy for burst size bs), δ_RNA_: RNA degradation rate per cell [1/h], *M*: polymerase occupancy [RNAs/h], *v*: RNAP2 speed (estimated as 3 kb/min), *l*: gene length, TSS: active TSS at the moment of measurement. Further details can be found in Materials and Methods section.Quantification of stochastic gene expression for the indicated p53 target genes before (basal, gray) and 3 h (red), 6 h (blue), and 9 h (orange) after DNA damage (10 Gy IR). The fraction (f) of active promoters (proxy for burst frequency) increases, while the transcription rate (μ; proxy for burst size) at active TSS remains similar upon DNA damage for all time points. Left panel: The percentage of cells with active TSS is shown as stacked bar graphs. We subdivided the population in cells with strong TSS activity (> 75% of TSS active, solid colors) and those with partial TSS activity (at least one, but less than 75% of TSS active, shaded colors). The mean fraction of active promoters (ratio of all active TSS to the total number of genomic loci analyzed) is indicated above each bar. Right panel: Distributions of calculated transcription rates μ [RNAs/h] at active TSS are presented for each time point as probability density estimates (PDF, see Data Visualization section). The number of TSS analyzed is indicated in each plot (compare Fig EV2C).Mean degradation rates of indicated RNAs in transcriptionally active cells before (basal, gray) and 3 h (red), 6 h (blue), and 9 h (orange) after DNA damage (10 Gy IR) as calculated from smFISH data. RNA stability is not changing in the measured time frame upon DNA damage. The plot displays the average RNA degradation rate per cell [1/h] over time after DNA damage, calculated from model (C) in actively transcribing cells for each gene.Based on promoter activity, we allocated target gene promoters along three archetypical expression patterns illustrated by a schematic triangle.Amount of p53 bound to indicated target gene promoters before (basal, gray) and 3 h (red), 6 h (blue), and 9 h (orange) after DNA damage (10 Gy IR) as measured by ChIP. The amount of bound p53 was calculated as percentage of input and normalized to the time point of the first p53 peak at 3 h. Individual data points (mean values of triplicate quantification in qRT–PCR measurements) from 3 to 4 biological repeats are shown as dots; mean values are displayed as black horizontal lines. Dashed lines serve as guide to the eyes. We could not detect p53 binding above IgG controls at the published p53 response element in the PPM1D promoter (indicated by n.d.) Source data are available online for this figure.

At an individual promoter, transcription can be either continuous or a stochastic process with episodic periods of activity and silent promoter states (bursty transcription) (Golding *et al*, 2005; Raj *et al*, 2006; Zenklusen *et al*, 2008; Singh *et al*, 2010; Suter *et al*, 2011; Dar *et al*, 2012; Coulon *et al*, 2013). To investigate whether p53 target genes encounter bursty or continuous transcription, we quantified the dispersion of mRNAs for all analyzed p53 targets in A549 cells. We observed that the corresponding distributions deviated from Poisson‐like dispersions expected for constitutively active promoters (Fano_mRNA_ ≫ 1; Fano_pois _= 1; Figs 1E and 2C and Appendix Fig S5C) (Dar *et al*, 2016; Singh *et al*, 2012). Single‐cell mRNA measurements of p53 targets therefore suggest stochastic transcription under basal and induced conditions (Peccoud & Ycart, 1995; Kepler & Elston, 2001). Despite high nuclear RNA counts for some targets such as BAX, MDM2, and CDKN1A, the nuclear‐to‐cytoplasmic ratio of mRNAs did not change for most of the analyzed targets upon IR (Appendix Fig S7), indicating that nuclear export is not limiting at this time scale. Only for DDB2, we observed a marked increase in nuclear RNAs upon irradiation, while at later time points, the nuclear‐to‐cytoplasmic ratio remained constant as for the other target genes. On the level of promoter activity, RNA numbers per cell can rise by more frequent activation, longer active periods, or a higher rate of transcription during active periods (Fig 3A and B) (Raj *et al*, 2008; Larson *et al*, 2011; Lionnet & Singer, 2012). Analyses of the *random telegraph* and related models provided characteristic noise profiles associated with these molecular events (Pedraza & Paulsson, 2008; Dar *et al*, 2012; Zoller *et al*, 2015). For example, an increase in mean mRNA expression via more frequent promoter activation alone would lead to reduced gene expression noise (measured as CV^2^) scaling according to the equation CV^2 ^= *b*/<mRNA> with *b* = μ/*k*_on_ (see Fig 3A and B, assuming an overall low frequency of promotor activity [*k*_off_ ≫ *k*_on_]) (Dar *et al*, 2016). We analyzed the CV^2^ versus mean relationship for all p53 targets and observed a trend to attenuated or reduced noise with increasing mean RNA numbers (Fig EV1D). Most measurements remained in a corridor around the expected noise scaling. In some cases, for example for MDM2, we observed a stronger deviation from the expected scaling at late time points (Fig EV1D). For the lowly expressed genes PPM1D and DDB2, gene expression noise at basal level deviated from the scaling expected from measurements in damaged cells (Fig EV1D). In general, this analysis points toward a regulation of the frequency of promoter activation and suggests gene‐specific regulation patterns (Singh *et al*, 2012; Dar *et al*, 2016).

**Figure EV2:**
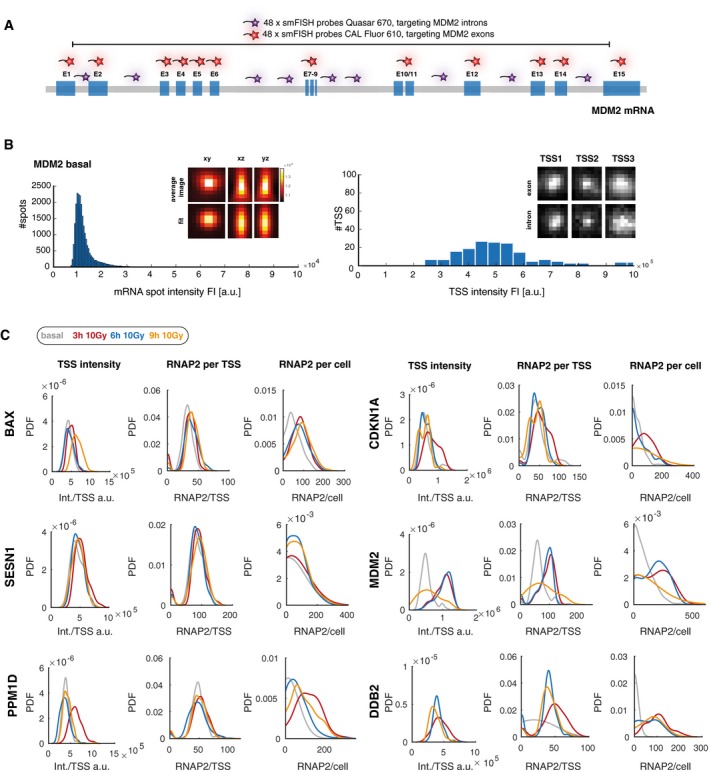
Calculation of RNAP2 occupancy and transcription rate from TSS intensity Schematic representation of smFISH co‐staining to identify TSS. Exon probes are labeled with CAL Fluor Red 610 and intron probes with Quasar 670. The latter probes are spread over several introns.Left panel: Histogram of the fluorescence intensity (FI) distribution for MDM2 RNA spots in basal state as an example. Calculated images in *xy*,* xz*, and *yz* dimensions of the average MDM2 RNA spot generated by FISH‐quant are depicted (upper row) as well as the corresponding fits (lower row). The FI intensity is indicated by a heat map. Right panel: Histogram showing the distribution of the FI for identified TSS transcribing MDM2 RNAs in basal state as an example. Image clippings show examples of intron and exons staining of three MDM2 TSS in basal state. For visualization, images were maximum‐projected and brightness‐ and contrast‐enhanced.Quantified parameters of promoter activity for the indicated target genes before (basal, gray) and 3 h (red), 6 h (blue), and 9 h (orange) after DNA damage (10 Gy IR). The left panel for each target gene shows distributions for quantified TSS intensities from FISH‐quant displayed as probability density estimates (pdf) of all active TSS. Center panels indicate distributions of RNAP2 occupancies at individual TSS, right panels the RNAP2 occupancies in the whole cell as calculated from the relative intensity of a TSS and the average cytoplasmic mRNA intensity (see Materials and Methods section for details). These occupancies were used to calculate transcription rates per hour.

However, analyzing noise scaling can only provide indirect access to stochastic gene expression. Moreover, decreased gene expression noise can be caused by other processes in addition to modulation of promoter activation frequency (Zoller *et al*, 2015). We therefore aimed to measure transcription states unambiguously in single cells after IR. Previous work has shown that dual‐color labeling of introns and exons by smFISH in combination with mathematical modeling allows to quantify transcription rates, promoter states, and mRNA life times in fixed cells (Bahar Halpern *et al*, 2015b) (Fig 3C). Using the same approach, we designed a second library of smFISH probes for each target gene to identify active sites of transcription based on intron/exon co‐staining (Fig EV2A). The fraction of active promoters (burst frequency) can hence be calculated as the ratio of co‐stained nuclear dots and the expected number of genomic loci, while the rate of transcription (burst size) is inferred from fluorescence intensity of nascent RNAs at active start sites (Figs 3C and EV2B) (Raj *et al*, 2008; Bahar Halpern *et al*, 2015b). In A549 cells, we detected sites of active transcription only inside nuclei as expected. They varied in number and fluorescence intensity, as introns are spliced and degraded co‐transcriptionally (Vargas *et al*, 2011; Levesque & Raj, 2013). As systematic co‐localization analysis showed more than two transcriptional start sites (TSS) for most p53 targets (Appendix Fig S8), we validated the maximal number of genomic loci for p53 target genes independently in A549 cells by DNA FISH (Appendix Fig S9, Dataset EV1).

**Figure EV3:**
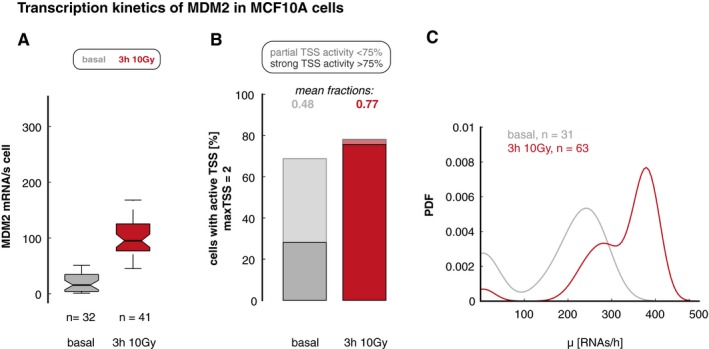
Bursting kinetics of MDM2 in MCF10A cells We quantified MDM2 RNAs per cell before (basal, gray) and 3 h (red) after DNA damage (10 Gy IR) in diploid non‐transformed MCF10A cells. Boxplots show smFISH measured RNA counts from single‐cell analysis of gene expression (see Data Visualization section); lines indicate medians of distributions; boxes include data between the 25^th^ and 75^th^ percentiles; whiskers extend to maximum values within 1.5× the interquartile range. Notches represent 5% confidence intervals for the median. The number of measured cells is indicated as *n*.The percentage of MCF10A cells with active MDM2 TSS, subdivided into populations with strong (> 75% of TSS, solid colors) and weak (< 75% of TSS, shaded colors) activity, is shown as stacked bar graphs; the mean fraction of active promoters is indicated above each bar. As for A549 cells, we observed an increase in the fraction of active MDM2 promoters (as a proxy for burst frequency) upon DNA damage.Distributions of calculated transcription rates at active MDM2 TSS are presented for each time point as probability density estimates (PDF, see Data visualization section). The transcription rates increased 3 h after DNA damage.

To analyze how stochastic bursting at target gene promoters changes with pulsatile p53 after IR, we characterized the fraction of active promoters, RNAP2 occupancy (M), transcription rate (μ) [RNAs/h], and RNA stability as degradation rate (δ_RNA_) [1/h] (Figs 3D and E, and EV2C and Appendix Fig S10, see Materials and Methods section for details). The fraction of active promoters serves as a proxy for the burst frequency, while the transcription rate approximates burst sizes. For all p53 target genes, we detected a strong increase in the fraction of actively transcribing promoters with the first p53 pulse (Fig 3D). When p53 levels decreased to basal level at 6 h, we saw that the MDM2, BAX, DDB2 as well as to a lesser extent the SESN1 promoter retained their increased fraction of active promoters indicating sustained high burst frequencies. In contrast, we detected a lower number of CDKN1A and PPM1D transcription sites. Interestingly, p53 accumulation during the second pulse was not linked to an up‐regulation in the fraction of active promoters for all targets. RNAP2 occupancy and transcription rate per TSS did not change strongly upon IR for all time points after 3 h (Figs 3D and EV2C). Furthermore, we did not observe noticeable changes in RNA stability upon IR for most of the selected genes and time points upon IR (Fig 3E). Only for DDB2, we observed an increase in RNA degradation upon DNA damage that remained high at later time points. This may explain the altered ratio of nuclear and cytoplasmic DDB2 RNA observed upon irradiation (Appendix Fig S7) and is consistent with previously reported post‐transcriptional regulation of DDB2 mRNA location and stability (Melanson *et al*, 2013). To compare our results from A549 cells to p53‐mediated gene expression in a diploid cell line, we performed smFISH of MDM2 in diploid MCF10A cells and obtained comparable results upon quantifying stochastic gene expression in response to IR (Fig EV3).

To help our understanding of the observed gene‐specific time‐dependent patterns of stochastic gene expression, we defined the three promoter archetypes “sustained”, “pulsatile”, and “transient” and assigned our set of target genes gradually along this spectrum (Fig 3F). For some genes, this resulted in a clear classification, as PPM1D, for example, showed obviously pulsatile promoter activity. For other genes such as DDB2 and SESN1, the assignment was more ambiguous, although they mostly trended toward one archetype of activity.

Transcriptional burst frequency can be modulated by concentration sensitive TF binding (Senecal *et al*, 2014; Kafri *et al*, 2016), interaction with distal *cis‐*regulatory elements (Fukaya *et al*, 2016), and the H3K27ac state of promoters (Nicolas *et al*, 2018). To test whether gene‐specific differences in transcriptional activity can be explained by differential p53 binding or changes in histone modifications, we performed ChIP experiments for selected target genes that resemble the transient and sustained promoter archetypes. P53 promoter binding reached a maximum at the first accumulation pulse as expected (Fig 3G). Surprisingly, it was not reduced to basal levels at 6 h. Instead, we found that for all analyzed promoters, p53 binding decreased gradually to intermediated levels, although its global concentration varied significantly between the trough and the second peak at 9 h. H3K27 methylation, a mark for repressed chromatin was initially reduced upon irradiation and remained constant at later time points for all promoters analyzed (Appendix Fig S11). H3K27ac increased upon irradiation to different extents at the analyzed promoters and remained at high levels at later time points without notable differences (Appendix Fig S11). Importantly, the observed differences in initial H3K27ac accumulation did not correlate with the observed expression pattern of the corresponding target genes.

### P53 dynamics affect stochastic transcription

Our results so far suggested a gene‐specific shift in p53's potency as a transcriptional activator after IR despite continuous promoter binding. As previous work has correlated stimulus‐dependent p53 dynamics with cell fate‐specific gene expression (Purvis *et al*, 2012), we investigated how modulation of p53 dynamics after the first peak affects bursting kinetics of the observed target gene archetypes. To this end, we used small molecular inhibitors in combination with IR to tune the p53 response into transient or sustained dynamics and tested four representative targets from our gene set: MDM2, BAX, PPM1D, and CDKN1A.

**Figure 4:**
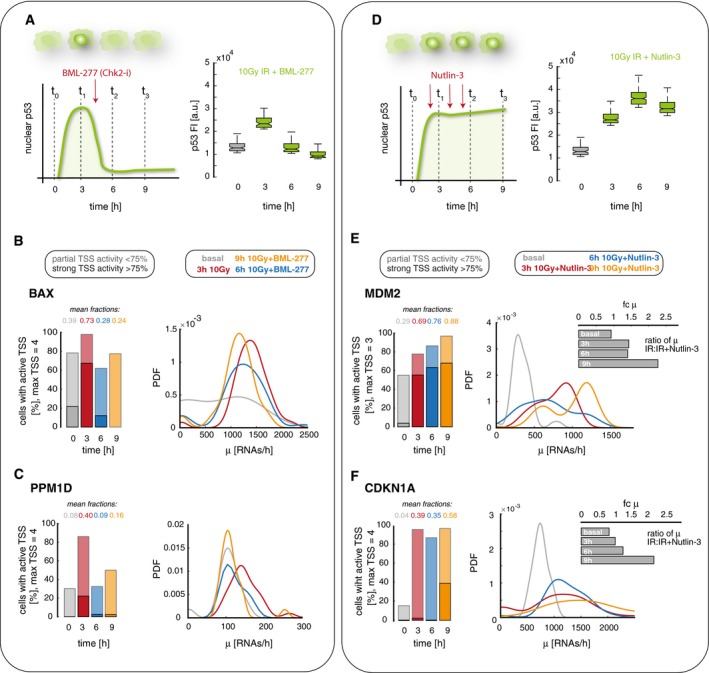
Promoter archetypes change upon modulation of p53 dynamics through small molecule inhibitors AChk2 inhibition with the small molecule BML‐277 induces transient p53 dynamics with only one pulse after 10 Gy IR. A schematic illustration of the experimental setup and quantification of p53 levels in A549 wild‐type cells after irradiation with 10 Gy IR and addition of 10 μM BML‐277 by immunofluorescence staining (see Materials and Methods section for details) are shown as box plots (see Data visualization section).B, CWe quantified promoter activity of BAX (B, sustained archetype) and PPM1D (C, pulsatile archetype) before (basal, gray) and 3 h (red), 6 h (blue), and 9 h (orange) after irradiation with 10 Gy IR and inhibition of the second p53 pulse by Chk2 inhibition. Left panel: The percentage of cells with active TSS, subdivided into populations with strong (> 75% of TSS, solid colors) and weak (< 75% of TSS, shaded colors) activity, is shown as stacked bar graphs, the mean fraction of active promoters is indicated above each bar. Right panel: Distributions of calculated transcription rates at active TSS are presented for each time point as probability density estimates (PDF, see Data visualization section). The fraction of active promoters was reduced at 6 and 9 h after irradiation; the transcription rate was not notably affected.DSequential treatment with Nutlin‐3 converts pulsatile p53 dynamics into sustained nuclear levels. A schematic illustration of the experimental setup and quantification of p53 levels in A549 *wild‐type* cells after irradiation with 10 Gy IR and sequential treatment with 0.75 μM Nutlin‐3 at 2.5 h, with 2.25 μM at 3.5 h and 4 μM at 5.5 h post‐IR based on immunofluorescence staining (see Materials and Methods section for details) are shown as box plots (see Data visualization section).E, FWe quantified promoter activity of MDM2 (E, transient archetype) and CDKN1A (F, transient archetype) before (basal, gray) and 3 h (red), 6 h (blue), and 9 h (orange) after irradiation with 10 Gy IR and sequential Nutlin‐3 treatment. Left panel: The percentage of cells with active TSS, subdivided into populations with strong (> 75% of TSS, solid colors) and weak (< 75% of TSS, shaded colors) activity, is shown as stacked bar graphs, the mean fraction of active promoters is indicated above each bar. Right panel: Distributions of calculated transcription rates at active TSS are presented for each time point as probability density estimates (PDF, see Data visualization section). The relative fraction of active promoters strongly increased, changing transient to sustained archetypes. The transcription rate increased as well both compared to basal levels and to previous experiments with pulsatile p53 dynamics (inset, fold change relative to IR alone for each time point). Source data are available online for this figure.

**Figure EV4:**
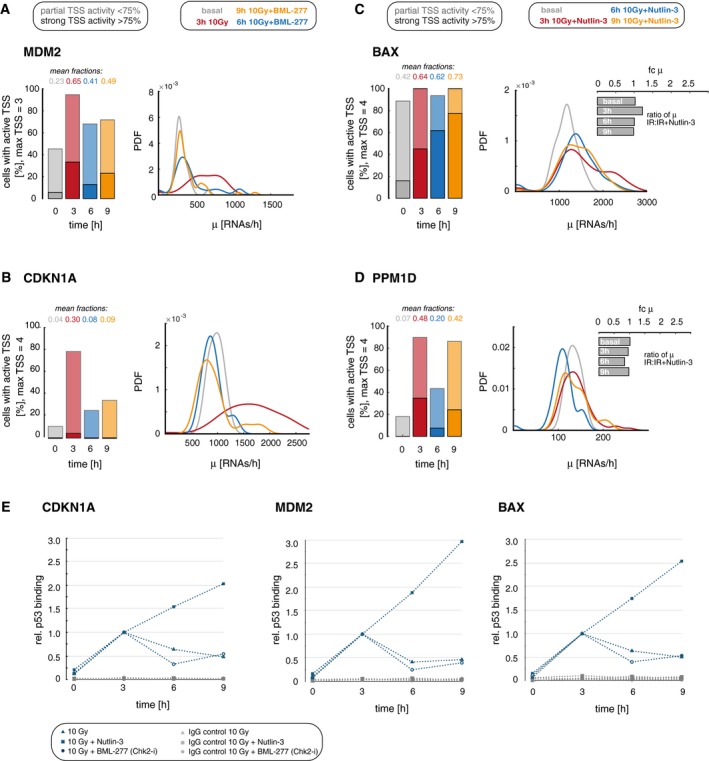
Quantification of bursting kinetics of target genes upon modulated p53 dynamics A, BWe quantified promoter activity of MDM2 (A, transient archetype) and CDKN1 (B, transient archetype) before (basal, gray) and 3 h (red), 6 h (blue), and 9 h (orange) after irradiation with 10 Gy IR and inhibition of the second p53 pulse by Chk2 inhibition. Left panel: The percentage of cells with active TSS, subdivided into populations with strong (> 75% of TSS, solid colors) and weak (< 75% of TSS, shaded colors) activity, is shown as stacked bar graphs, the mean fraction of active promoters is indicated above each bar. Right panel: Distributions of calculated transcription rates at active TSS are presented for each time point as probability density estimates (PDF, see Data visualization section). As both target genes were grouped in the transient archetype, no obvious changes in promoter activity were observed.C, DWe quantified promoter activity of BAX (C, sustained archetype) and PPM1D (D, pulsatile archetype) before (basal, gray) and 3 h (red), 6 h (blue), and 9 h (orange) after irradiation with 10 Gy IR and sequential Nutlin‐3 treatment. Left panel: The percentage of cells with active TSS, subdivided into populations with strong (> 75% of TSS, solid colors) and weak (< 75% of TSS, shaded colors) activity, is shown as stacked bar graphs; the mean fraction of active promoters is indicated above each bar. Right panel: Distributions of calculated transcription rates at active TSS are presented for each time point as probability density estimates (PDF, see Data visualization section). For BAX, both transcription parameters remained high. Interestingly, in the contrary to our observations for target genes that change their gene expression mode in response to Nutlin‐3 treatment, the transcription rate did not change notable for BAX. Surprisingly, the same holds true for PPM1D, a gene that we assigned to the pulsatile archetype.ETo measure relative p53 binding at different target gene promoters in perturbed and unperturbed cells, we performed ChIP experiments in the context of Nutlin‐3 and BML‐277 treatment for all p53 target genes at the indicated time points after 10 Gy IR‐CDKN1A (left), MDM2 (center), and BAX (right; we could not detect any p53 binding at the published response element in the PPM1D promoter in repeated experiments). The amount of bound p53 was calculated as percentage of input and normalized to the time point of the first p53 peak at 3 h. Gray symbols indicate the corresponding IgG control. Interestingly, pulsatile p53 and inhibition of the second p53 pulse both lead to a gradual decrease of p53 binding after the first peak, Nutlin‐3 treatment increases p53 binding at these promoters and may thereby contribute to the observed increase in promoter activity.

First, we generated a transient p53 response with only one accumulation pulse using the Chk2‐inhibitor BML‐277 at 4 h after IR (Fig 4A). This allowed us to focus on gene‐specific differences during the second p53 pulse, leaving the initial DNA damage regulation of p53 and transcriptional activation of targets unchanged. Our analysis revealed that both PPM1D (resembling the *pulsatile* archetype) and BAX (resembling the *sustained* archetype) had reduced frequencies of promoter activity when the p53 response was transient, while the transcription rate remained similar (Figs 4B and C, and EV4A and B). A direct comparison at the 9 h time point showed that in Chk2 inhibitor‐treated cells, the fraction of active BAX TSS was strongly reduced compared to pulsatile p53, while we observed a weaker effect at the PPM1D promoter. This indicated that the reoccurrence of a second p53 pulse is necessary to keep those genes in an active transcription mode after the first pulse. Notably, target gene expression is decreased significantly for genes that showed a trend to *transient* promoter activity as well when further p53 pulsing is prevented (Fig EV4A and B).

Next, we asked how persistent nuclear p53 accumulation affects stochastic gene expression. To test this, we used an increasing sequence of the small molecule MDM2 inhibitor Nutlin‐3 (Vassilev *et al*, 2004) after IR to change p53 dynamics from a pulsing to a sustained regime (Fig 4D) (Purvis *et al*, 2012). Upon Nutlin‐3 treatment, the frequency of promoter activation at the 9 h time point increased for all targets (Figs 4E and F, and EV4C and D) including MDM2 and CDKN1A that resembled the *transient* promoter archetype when p53 was pulsing. Interestingly, when p53 was kept at high levels for extended time periods, we did not solely detect an increase in the fraction of active promoters of 2.1‐fold for CDKN1A and 1.9‐fold for MDM2, but also an increase in transcription rates that was > 2‐fold higher than in response to pulsatile p53 (IR only; Fig 4E and F). This indicates that sustained nuclear p53 leads to a mechanistic shift in promoter regulation for targets with *transient* promoter activity via a different mechanism than upon IR only treatment. When we compared relative p53 binding under transient and sustained p53 conditions by ChIP, we further detected an increase at all analyzed promoters for sustained p53 (BAX, CDKN1A, and MDM2; Fig EV4E). Notably, transient p53 accumulation upon Chk2 inhibition did not lead to a complete loss of p53 binding at these promoters, but to comparable binding profiles as pulsatile p53 (Fig EV4E).

### The K370/382 methylation–acetylation switch contributes to transient promoter activity during the 2^nd^ p53 pulse

**Figure 5:**
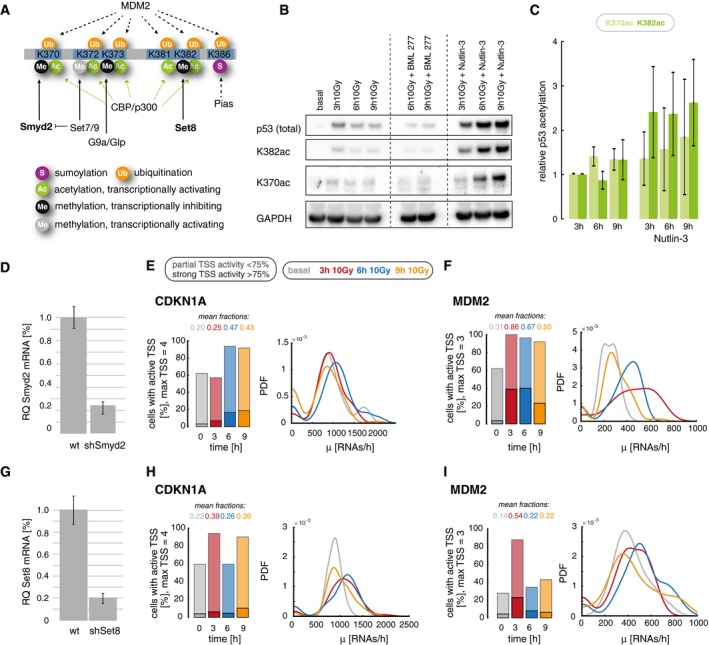
The interplay of p53's C‐terminal lysine acetylation and methylation regulates transiently expressed target genes in response to IR AA schematic illustration of p53's C‐terminal modifications and described functional implications, including key regulatory enzymes.BTotal p53, p53 acetylated at K382 and K370 as well as GAPDH were measured by Western blot at indicated time points in the context of different p53 dynamics: pulsing p53 (10 Gy IR), transient p53 (10 Gy IR + BML‐277, central lanes), and sustained p53 (10 Gy IR + Nutlin‐3, right lanes). See Fig 3 and Materials and Methods section for details.CThe relative change in p53 acetylation at K370 (light green) and K382 (dark green) was quantified from Western blot and normalized to the abundance 3 h post‐IR. Means and propagated standard errors from three independent experiments are indicated. Acetylation increased over time in the context of sustained p53. See also Appendix Fig S12.DThe p53‐K370 methylase Smyd2 was down‐regulated in a clonal stable A549 cell line expressing a corresponding shRNA. Transcript levels were measured in wild‐type and knockdown cells by qRT–PCR. Mean levels and standard deviation from technical triplicates are indicated.E, FPromoter activity of CDKN1A (E) and MDM2 (F) was quantified in Smyd2 knockdown cells before (basal, gray) and 3 h (red), 6 h (blue), and 9 h (orange) after DNA damage (10 Gy IR). Left panel: The percentage of cells with active TSS, subdivided into populations with strong (> 75% of TSS, solid colors) and weak (< 75% of TSS, shaded colors) activity, is shown as stacked bar graphs; the mean fraction of active promoters is indicated above each bar. Right panel: Distributions of calculated transcription rates at active TSS are presented for each time point as probability density estimates (PDF, see Data visualization section). We measured a higher fraction of active promoters upon damage compared to A549 wild‐type cells (Fig 3), while transcription rates remained unchanged. See Fig EV5A for corresponding changes in p53 acetylation patterns.GThe p53‐K382 methylase Set8 was down‐regulated in a clonal stable A549 cell line expressing a corresponding shRNA. Transcript levels were measured in wild‐type and knockdown cells by qRT–PCR. Mean levels and standard deviation from technical triplicates are indicated.H, IPromoter activity of CDKN1A (E) and MDM2 (F) was quantified in Set8 knockdown cells before (basal, gray) and 3 h (red), 6 h (blue), and 9 h (orange) after DNA damage (10 Gy IR). Left panel: The percentage of cells with active TSS, subdivided into populations with strong (> 75% of TSS, solid colors) and weak (< 75% of TSS, shaded colors) activity, is shown as stacked bar graphs; the mean fraction of active promoters is indicated above each bar. Right panel: Distributions of calculated transcription rates at active TSS are presented for each time point as probability density estimates (PDF, see Data visualization section). We measured a higher fraction of active promoters upon damage compared to A549 wild‐type cells (Fig. 3), while transcription rates remained unchanged. See Fig EV5A for corresponding changes in p53 acetylation patterns. Source data are available online for this figure.

The regulatory potential of p53's highly unstructured C‐terminal domain (CTD) has been in the focus of numerous studies aiming to disentangle its functions in modulating gene expression (Sullivan *et al*, 2018). It has been shown that post‐translational modifications of the CTD play a central role in regulating target gene transcription (Bode & Dong, 2004; Sims *et al*, 2004; Loffreda *et al*, 2017). In particular, acetylation of lysine residues K370, K372/73, and K381/82 by p300/CBP has been associated with a transcriptionally active state (Fig 5A) (Gu *et al*, 1997). In contrast, methylation of K370, K373, and K382 inhibits target gene expression (Huang *et al*, 2006; Shi *et al*, 2007). In absence of DNA damage, repressive methylation marks keep p53 transcriptionally inactive. Induction of DSBs induces a rapid change toward CTD acetylation that allow target gene expression (Berger, 2010; Loewer *et al*, 2010). To test whether C‐terminal acetylation contributes to transient MDM2 and CDKN1A expression during the p53 response, we induced pulsatile, transient, and sustained p53 accumulation as described above (see Fig 4) and analyzed p53 acetylation at K370 and K382 by Western blot (Fig 5B and Appendix Fig S12). We observed that K382ac levels were higher under sustained p53 conditions compared to pulsatile p53 (Fig 5C), suggesting a stabilization of acetylated p53 due to reduced protein turnover (Li *et al*, 2002) or reduced lysine methyltransferase (KMT) activity.

**Figure EV5:**
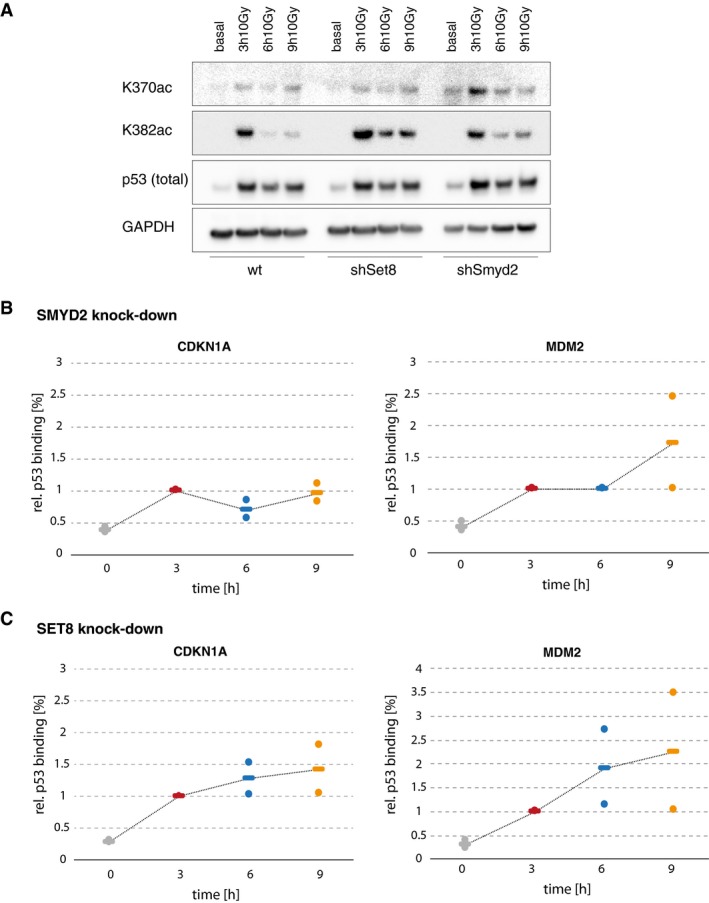
Smyd2 and Set8 activities affect p53 nuclear dynamics and promoter binding AWestern blot of acetylated p53 (K370/K382) in A549 Smyd2 and Set8 knockdown cells compared to wild‐type cell lines shows an increase in acetylation specifically at later time points in the DNA damage response. Dynamics of total p53 remained pulse like. GAPDH is shown as loading control.B, CAmount of p53 bound to CDKN1A and MDM2 promoters in A549 Smyd2 (B) and Set8 (C) knockdown cells before (basal, gray) and 3 h (red), 6 h (blue), and 9 h (orange) after DNA damage (10 Gy IR) as measured by ChIP. The amount of bound p53 was calculated as percentage of input and normalized to the time point of the first p53 peak at 3 h. Individual data points (mean values of triplicate quantification in qRT–PCR measurements) from two biological repeats are shown as dots; mean values are displayed as black horizontal lines. Dashed lines serve as guide to the eyes. We observed an increase in promoter binding at later time points similar to the results after Nutlin‐3 treatment.

Next, we asked how this change in K370/K382 modification state affects stochastic bursting of target genes that we allocated to the *transient* promoter archetype, specifically CDKN1A and MDM2. To this end, we generated stable clonal A549 shRNA knockdown cell lines, reducing the RNA levels of the corresponding methyl transferases Smyd2 and Set8 to 22 and 20%, respectively (Fig 5D and G). Notably, loss of methylation led to an increase in acetylation at the corresponding residue upon irradiation (Fig EV5A). We then characterized the frequency of active promoters and transcription rates at the same time points as previously after IR (Fig 5E, F, H and I). While we did not detect strong changes in the fraction of active promoters at basal condition and 3 h after IR compared to A549 *wild‐type* cells, the mean fraction of active promoters at 9 h was increased from 23 to 43% for CDKN1A and from 46 to 50% for MDM2 in the context of Smyd2 shRNA knockdown compared to IR irradiated A549 wild‐type cells (Fig 5E and F). Consistently, we observed increased binding of p53 to the corresponding REs in the CDKN1A and MDM2 promoters 6 and 9 h post‐damage induction (Fig EV5B, compare to Fig EV4E). Even though the increase in burst frequency at 9 h after sequential treatment with Nutlin‐3 was even stronger and may include also an impact of the change in integrated p53 abundance on bursting, this suggests that Smyd2‐mediated methylation contributes to reduced transcription during the second p53 pulse for *transient* p53 targets. Notably, in the context of Set8 knockdown (Figs 5H and I, and EV5C), we also detected extended expression of CDKN1A through frequency modulation and increased p53 binding to promoters with *transient* expression profiles, although less prominently than upon Smyd2 knockdown. As it has been previously shown that the different lysine residues in p53's CTD act in concert and embed redundant mechanisms to provide robustness, combinatorial effects of different residues or additional co‐factor interaction is likely to lead to transient transcription of MDM2 and CDKN1A.

## Discussion

P53 and other major TFs show stimulus‐specific dynamics correlated with cell fate. While the underlying molecular networks and response mechanisms have been largely characterized, it remains elusive how these proteins regulate gene expression mechanistically at specific promoters in individual cells. In this work, we show that p53‐dependent transcription upon IR is intrinsically stochastic and regulated mainly by burst frequency. For our selected panel of p53 targets, we observed that differential regulation of the *on:off* rate of promoter bursting contributes to gene‐specific dynamics of transcriptional activity. These dynamics could be allocated gradually along a spectrum defined by three archetypes of promoter activity: *transient*,* pulsatile,* and *sustained*. Archetypes differed mainly in their response to the second pulse of p53 accumulation upon DNA damage. While target genes resembling the *pulsatile* archetype tended to have low overall expression levels, we could so far not define molecular criteria that would predict expression archetypes for other target genes. Neither the number and location of p53 binding sites nor their predicted or measured affinities correlated with the expression archetype in the set of selected target genes (Fig EV1A) (Veprintsev & Fersht, 2008). Moreover, genes involved in the different response pathways contributed to all archetypes, indicating that archetypes are not directly correlated with cell fate. However, our analysis is so far limited to a small subset of p53 target genes. Further studies of promoter architecture, epigenetic states, and combinatorial control of transcription may help to reveal how gene‐specific modulation of bursting dynamics contributes to structuring the p53 response network upon damage induction.

Modulation of stochastic gene expression has previously been shown for other cellular processes. Stimulation of murine cells with serum or TGF‐β1 induced expression of the connective tissue growth factor (CTGF) gene by increasing the transcription rate and therefore burst sizes (Molina *et al*, 2013). Interestingly, initial activation of CTGF transcription upon serum starvation was followed by a long refractory period resembling transient expression of p53 targets. TGF‐β1 stimulation, in contrast, led to a sustained increase in burst sizes. Frequency modulation has been demonstrated for c‐fos dependent transcription after serum or zinc induction (Senecal *et al*, 2014), light‐controlled transcription by the White Collar Complex in Neurospora (Li *et al*, 2018), and dose‐dependent transcriptional regulation by ligand‐bound steroid receptors (Larson *et al*, 2013). Using targeted perturbations, it has further been shown that frequency modulation and polymerase pause release are key regulatory aspects of transcriptional regulation, while RNAP2 recruitment occurs subsequent to burst initiation (Bartman *et al*, 2019). The simplest model to explain frequency modulation is that the state of a gene is regulated by the *on:off* rate of TF binding to the RE, while the transcription rate in the active state depends on other processes downstream of RE binding. This model suggests that the occupancy of *cis‐*regulatory elements by sequence‐specific TFs can serve as a proxy for transcriptional output (Ptashne & Gann, 2001). Accordingly, we observed coordinated increases in promoter binding and burst frequencies for the initial p53 response to IR and a dependency on recurring p53 accumulation for the *pulsatile* and *sustained* archetypes. However, gene‐specific expression patterns at later time points could not be explained by the relatively uniform intermediate binding levels found at all promoters analyzed. Interestingly, we also observed a disconnect between nuclear protein levels and DNA binding after the first pulse of p53 accumulation. A similar disconnect between TF levels and gene expression has been observed for TGF‐β1 induced CTGF expression (Tidin *et al*, 2019). These observations argue against a simplified model of affinity‐based regulation of bursting parameters and suggest other regulatory mechanisms.

Surprisingly, we observed a gradual decrease in p53 promoter binding after the first accumulation peak instead of a tight coupling to p53 levels even in absence of a second p53 pulse (Fig EV4E). How is p53 stabilized at promoters while total p53 levels are reduced to basal state, despite fast binding kinetics of only a few milliseconds (Loffreda *et al*, 2017)? As relative binding curves were similar for all target genes, a global increase in DNA binding activity or selective stabilization of chromatin‐bound p53 can be assumed. For example, it has been previously shown that tetramerization of p53 leads to a stabilization of DNA binding in response to DNA damage (Gaglia & Lahav, 2014). In future studies, it would be interesting to investigate by fluorescence correlation spectroscopy if an increase in the tetrameric p53 population can be observed at 6 h after IR compared to basal state.

Another possibility would be that the promoter‐associated p53 pool shows dominant PTMs at C‐terminal lysine residues that are mutually exclusive with MDM2‐dependent ubiquitination. DNA damage induces numerous post‐translational modifications of the TF that lead to a stabilization of p53 levels in the nucleus but fulfill a variety of other functions as well. However, in our ChIP experiments, we only resolved total p53. We show that burst frequency is modulated in response to IR and that p53 network perturbations associated with an increase in K370 and K382 acetylation are correlated with higher burst frequencies and, partially, higher burst sizes at p53 target gene promoters. We can only hypothesize about potential mechanisms that lead to these changes as the function of p53's CTD has been controversially discussed in the literature (Laptenko *et al*, 2016; Sullivan *et al*, 2018) and its intrinsically disordered topology allows a variety of functions and interactions (Fuxreiter *et al*, 2008). The CTD binds DNA in a non‐sequence‐specific manner due to the basic nature of its many lysine residues. This allows sliding along the DNA and promotes and stabilizes the sequence‐specific binding of the DNA binding domain at p53 REs (McKinney & Prives, 2002; Laptenko *et al*, 2015). Further, it has been shown to interact with many co‐regulatory factors that strongly dependent on the post‐translational modification state, which could additionally affect stochastic bursting.

Using perturbation studies, we could demonstrate that *transient* expression of CDKN1A and MDM2 are differentially regulated via opposing acetylation and methylation of K370 and K382 residues and can be tuned to different modes of stochastic expression. In line with our findings, a previous study indicated reduced p53 promoter binding and transcription through Smyd2 mono‐methylation of K370 (Huang *et al*, 2006). However, as we still see over 50% p53 promoter binding at 9 h post‐IR, a reduction in promoter binding mediated through Smyd2‐dependent K370me cannot solely explain the *transient* expression of MDM2 and CDKN1A in A549 cells. Moreover, Set7/9 activity leading to inhibition of Smyd2 has been shown to be dynamically regulated during the first p53 pulse after IR (Ivanov *et al*, 2007). Furthermore, K382 mono‐methylation by Set8 induces binding of the chromatin compaction factor L3MBTL1 at CDKN1A and PUMA promoters (West *et al*, 2010). In contrast, CTD acetylation and DNA binding have been characterized in population studies, leading to controversial results about an increase or decrease in binding affinity (Gu & Roeder, 1997; Nakamura *et al*, 2000; Friedler *et al*, 2005). However, acetylation of C‐terminal lysine residues has been linked to its transcriptional activity (Tang *et al*, 2008). Recently, sophisticated single‐molecule studies revealed that transient p53‐chromatin interactions are modulated upon activation and interaction times reflect the acetylation state of C‐terminal p53 residues (Loffreda *et al*, 2017). Furthermore, it has also been suggested that an interaction in nuclear aggregates between RNAP2 CTD and disordered regions of TFs such as p53's CTD can lead to recruitment and transactivation (Sullivan *et al*, 2018) of RNAP2 into an elongation competent form (Kwon *et al*, 2013). It is possible to speculate that these mechanisms affect stochastic bursting by a direct or indirect increase in transcription initiation and PIC stability or release of paused RNAP2. However, to our knowledge none of these mechanisms have yet been correlated to repeated pulses of p53 on longer time scales during the DNA damage response or stochastic bursting at the respective promoters. Notably, it has previously also been suggested that Smyd2 affects the RNAP2 elongation rate independent of p53 (Brown *et al*, 2006). However, we did not see significant changes in transcription rates upon Smyd2 knockdown that would be expected from altered RNAP2 elongation rates (Fig 5). Therefore, p53‐independent transcriptional inhibition of RNAP2 elongation may only play a minor role in regulation of *transient* p53 targets under our experimental conditions (Brown *et al*, 2006).

Our data indicate that C‐terminal modifications of p53 change between the first and the second p53 pulse. Preventing protein turnover using Nutlin‐3 resulted in different promoter regulation and stochastic bursting modalities of p53 target genes, indicating stabilization and accumulation of otherwise transient PTMs. The differences in p53's first and second pulse activity hint toward a change in upstream processes that re‐initiate the p53 response after the first trough. To date, the common view on repeated pulses of nuclear p53 is that ATM and other kinases upstream of p53 are re‐activated as long as DNA damage is still present (Batchelor *et al*, 2008). A change in p53's PTM patterns may thereby hint toward either another layer of regulation downstream of PI3K‐like kinases or other co‐regulatory factors that reduce p53 PTMs. Besides C‐terminal acetylation, p53 S20 or S46 phosphorylation may also contribute to different archetypes, as both of these modifications correlate with promoter‐specific binding of p53 after etoposide or actinomycin D treatment of U‐2 OS cells (Smeenk *et al*, 2011).

While we focused on the role of p53 modifications in regulating stochastic target gene expression, other mechanisms have been suggested to control gene‐specific promoter activity. For example, long‐range enhancer–promoter interactions or forced chromatin looping influence burst frequency in other systems (Bartman *et al*, 2016; Fukaya *et al*, 2016) and it has been hypothesized that enhancer–promoter contacts are necessary for every burst (Chen *et al*, 2018). A recent study could further show that enhancer–promoter interactions of the Hbb1‐1 gene increase burst frequency (Bartman *et al*, 2019). Histone methylation preserves burst frequency between mother and daughter cells (Muramoto *et al*, 2012), and histone acetylation can affect transcriptional bursting, mainly burst frequency (Harper *et al*, 2011; Suter *et al*, 2011; Nicolas *et al*, 2018). Furthermore, nucleosome remodeling has been suggested to be rate limiting for transcriptional activation (Boeger *et al*, 2008; Kim & O'Shea, 2008). Markers of repressive chromatin architecture, such as CTCF boundaries, cohesin, and inhibitory histone marks, correlate with inducible expression of p53 targets and have been suggested to play a role in gene‐specific dampening of p53‐dependent expression upon damage (Su *et al*, 2015). It will be interesting to investigate in future studies to which extent these mechanisms contribute to regulating gene‐specific stochastic transcription of p53 target genes in the response to DNA damage. Interestingly, previous studies have suggested that expression patterns of p53 targets are mainly determined by RNA and protein stability (Porter *et al*, 2016; Hafner *et al*, 2017; Hanson *et al*, 2019), while changes in p53 dynamics are filtered at target gene promoters by distinct activation thresholds (Harton *et al*, 2019). Based on our model of single‐cell TSS activity, we suggest that direct transcriptional regulation of stochastic bursting provides an important contribution as well.

In general, our data highlight that p53 pulses allow for a broader diversity in gene‐specific stochastic transcriptional regulation compared to sustained p53 dynamics, which induces transcription of most p53 targets at high rates. Pulsatile TF nuclear dynamics thereby allow for differential promoter archetypes and fine‐tuning of transcription. Besides the pre‐dominant hypothesis of robustness of cellular signaling, this may play an important role for expanding the regulatory potential of TFs at target promoters over time.

## Materials and Methods

### Reagents and Tools table

**Table:** 

Reagent/resource	Reference or source	Identifier or catalog number
**Experimental models**		
A549 cells	ATCC	CCL‐185
MCF10A cells	ATCC	CRL‐10317
**Recombinant DNA**		
pRetroSuper.puro	Brummelkamp *et al* (2002)	
**Antibodies**		
Rabbit anti‐GAPDH pAb (1:10,000)	Sigma‐Aldrich	Cat #G9545
Mouse anti‐p53 mAb (1:5,000)	Santa Cruz	Cat #sc‐126
Rabbit anti‐p53 pAb (5 μg per IP)	Santa Cruz	Cat #sc‐6243
Rabbit anti‐p53 pAb (5 μl per IP)	Cell Signaling	Cat #9282
Rabbit anti‐p53K70ac mAb (1:1000)	Abcam	Cat #ab183544
Rabbit anti‐p53K382ac mAb (1:2500)	Abcam	Cat #ab75754
Rabbit anti‐H3K27Ac pAb (5 μg per IP)	Abcam	Cat #ab4729
Rabbit anti‐H3K27Me3 pAb (5 μg per IP)	Millipore	Cat #7‐449
Goat anti‐rabbit‐HRP (1:10,000)	Thermo Fisher Scientific	Cat #G‐21234
Goat anti‐mouse‐HRP (1:10,000)	Thermo Fisher Scientific	Cat #G‐21040
Goat anti‐rabbit‐Alexa Fluor 647 (1:1,000)	Thermo Fisher Scientific	Cat #A‐21245
**Oligonucleotides and other sequence‐based reagents**		
qRT–PCR:		
BAX forward: CTGACGGCAACTTCAACTGG	This study	
BAX reverse: GATCAGTTCCGGCACCTTGG	This study	
MDM2 forward: AGA TGT TGG GCC CTT CGT GAG AA	This study	
MDM2 reverse: GCC CTC TTC AGC TTG TGT TGA GTT	This study	
CDKN1A forward: TGG ACC TGT CAC TGT CTT GT	Finzel *et al* (2016)	
CDKN1A reverse: TGG ACC TGT CAC TGT CTT GT	Finzel *et al* (2016)	
DDB2 forward: GCC ATC TGT CCA GCA GGG GC	This study	
DDB2 reverse: GGG GTG AGT TGG GTG CCA CG	This study	
SESN1 forward: AGA TGA GGC AGT TAC AGG AAT G	This study	
SESN1 reverse: ATG ACG AGA TAC AGC TCT TGC	This study	
PPM1D forward: ATA AGC CAG AAC TTC CCA AGG	This study	
PPM1D reverse: TGG TCA ATA ACT GTG CTC CTT C	This study	
SET8 forward CCC TTC CAC GGG CTG CTA C	Loewer *et al* (2010)	
SET8 reverse GTG CAG TTT GGT TTG GCA GTT CC	Loewer *et al* (2010)	
SMYD2 forward CCT CAA CGT GGC CTC CAT GTG	Loewer *et al* (2010)	
SMYD2 reverse TGG ATG ATC TTT GCC GTG AGC TAC	Loewer *et al* (2010)	
TP53 forward TGA CTG TAC CAC CAT CCA CTA	This study	
TP53 reverse AAA CAC GCA CCT CAA AGC	This study	
β‐ACTIN forward, GGC ACC CAG CAC AAT GAA GAT CAA	Finzel *et al* (2016)	
β‐ACTIN reverse, TAG AAG CAT TTG CGG TGG ACG ATG	Finzel *et al* (2016)	
ChIP assays:		
BAX forward: AAC CAG GGG ATC TCG GAA G	Sánchez *et al* (2014)	
BAX reverse: AGT GCC AGA GGC AGG AAG T	Sánchez *et al* (2014)	
MDM2 forward: GTT CAG TGG GCA GGT TGA CT	Sánchez *et al* (2014)	
MDM2 reverse: CGG AAC GTG TCT GAA CTT GA	Sánchez *et al* (2014)	
CDKN1A forward: AGC CTT CCT CAC ATC CTC CT	Sánchez *et al* (2014)	
CDKN1A reverse: GGA ATG GTG AAA GGT GGA AA	Sánchez *et al* (2014)	
DDB2 forward: CTC CAA GCT GGT TTG AAC	This study	
DDB2 reverse: CAC AGG TAG CCG AGC TAA G	This study	
SESN1 forward: GCC GCG GTC ATG TAA ATG AAA G	This study	
SESN1 reverse: GAC TTG TCC AGA CGA CAA TG	This study	
PPM1D forward: CGG ACA AGT CCA GAC ATC	This study	
PPM1D reverse: TTC GAC GAC GCC GAG AAG	This study	
BAC‐derived DNA FISH probes:		
BAX (RP11‐29G15)	Empire Genomics	
SESN1 (RP11‐26B10)	Empire Genomics	
DDB2 (RP11‐601F23)	Empire Genomics	
MDM2 (RP11‐1137N1)	Empire Genomics	
See Dataset EV2 for Stellaris probe sets		
**Chemicals, enzymes and other reagents**		
DRB (5,6‐dichlorobenzimidazole 1‐b‐D‐ribofuranoside)	Cayman	Cat # 1001030250
Chk‐2 inhibitor II, BML‐277	Sigma‐Aldrich	Cat # 220486
Nutlin‐3	Sigma‐Aldrich	Cat # N6287
Dynabeads Protein G	Thermo Fisher Scientific	Cat # 10004D
RNase A	Applichem	Cat # A2760
Precision Plus Protein Dual Color Standards	Bio‐Rad	Cat # 1610374
Trichostatin A	APExBio	Cat # A8183
Deacetylase Inhibitor Cocktail	MedChemExpress	Cat # HY‐K0030
Inhibitor Cocktail Plus	Roth	Cat # 3751.1
Phosphatase Inhibitor Cocktail 3	Sigma‐Aldrich	Cat # P0044
Alexa Fluor 488 *N*‐Hydroxysuccinimide	Thermo Fisher Scientific	#A‐20000
Hoechst‐33342	Thermo Fisher Scientific	#62249
**Software**		
MATLAB	MathWorks	
FIJI	Schindelin *et al* (2012)	
FISH‐quant	Mueller *et al* (2013)	
Trans Quant	Bahar Halpern and Itzkovitz (2016)	
**Other**		
Monarch® PCR & DNA Cleanup Kit	NEB	#T1030
WesternBright™ Quantum™	Advansta	K‐12042

### Methods and Protocols

##### Cell line and constructs

A549 cells were cultured in McCoy's medium supplemented with 10% fetal bovine serum, 1% penicillin, and streptomycin. When required, the medium was supplemented with selective antibiotics to maintain transgene expression (400 μg/ml G418, 50 μg/ml hygromycin, or 0.5 μg/ml puromycin). The A549 reporter and p53 knockdown cell lines have been described before (Finzel *et al*, 2016). To generate A549 knockdown cell lines for SMYD2 and SET8 and a MCF10a knockdown cell line for p53, we used previously published vectors based on pRetroSuper.puro to express corresponding small hairpin RNAs (Brummelkamp *et al*, 2002); Loewer *et al*, 2010). VSV‐G pseudotyped retroviral particles expressing SMYD2, SET8, or p53 shRNA were produced in 293T cells and subsequently used to infect A549 or MCF10A *wild‐type* cells. These cells were used as polyclonal populations in further experiments.

##### Antibodies and reagents

Stellaris probe sets for smFISH (Biosearch Technologies) were custom‐designed for intron and exon regions (see Dataset EV2 for oligo list) and conjugated with CAL Fluor 610 (Exons) and Quasar 670 (Introns). We used antibodies against total p53 (FL‐393, #6243 and DO‐1, #sc‐126) from Santa Cruz and against acetylated p53 (K373/382, #ab131442, ab62376) from Abcam. A fluorescent‐labeled secondary antibody conjugated with Alexa Fluor 647 as wells as Alexa Fluor 488 *N*‐Hydroxysuccinimide (NHS, 1‐hydroxy‐2,5‐pyrrolidinedione) and Hoechst‐33342 staining solution was purchased from Cell Signaling/Life Technologies (Thermo Fisher Scientific, #A‐21245, 20000). DRB (5,6‐dichlorobenzimidazole 1‐b‐d‐ribofuranoside) was purchased from Cayman (used at 10 μM, #1001030250), Chk‐2 inhibitor II BML‐277 (used at 10 μM) from and Nutlin‐3 (used at 0.75–4 μM, #N6287) from Sigma.

##### Single‐molecule FISH

A549 cells were cultured for 24 h on 18‐mm uncoated coverglass (thickness #1). After treatment, cells were washed on ice, fixed with 2% para‐formaldehyde (EM‐grade) for 10 min at room temperature, and permeabilized overnight with 70% ethanol at 4°C. Custom probe sets for smFISH (Biosearch Technologies) were hybridized at a final concentration of 0.1 μM probe following manufacturer's instructions overnight at 37°C. Following hybridization procedure, cells were washed and incubated with Alexa Fluor 488 *N*‐Hydroxysuccinimide (NHS‐AF88) for 10 min at RT for unspecific cytoplasmic protein staining, followed by Hoechst nuclear counterstain. Coverglasses were mounted on Prolong Gold Antifade (Molecular Probes, Life Technologies). Cells were imaged on a Nikon Ti‐inverted fluorescence microscope with an EMCCD camera (ANDOR, DU iXON Ultra 888), Lumen 200 Fluorescence Illumination Systems (Prior Scientific), and a 60× plan apo objective (NA 1.4) using appropriate filter sets (Hoechst: 387/11 nm excitation [EX], 409 nm dichroic beam splitter [BS], 447/60 nm emission [EM]; Alexa Fluor 488: 470/40 nm [EX], 495 nm [BS], 525/50 nm [EM]; CAL Fluor 610: 580/25 nm [EM], 600 nm [BS], 625 nm [EX]; Quasar 670: 640/30 nm [EX], 660 nm [BS], 690/50 nm [EM]). Images were acquired as multipoint of 21 z‐stacks of each cell (field of view) with 300 nm step‐width using Nikon Elements software. Quantification of RNA counts per cell was performed using FISH‐quant (Mueller *et al*, 2013) and custom‐written MATLAB software.

##### Analysis of smFISH data

Multicolor z‐stacks from Nikon Elements software were extracted into individual tif‐stacks and imported into FISH‐quant (Mueller *et al*, 2013). For nuclei and cytoplasmic segmentation, two approaches dependent on the quality of cytoplasmic staining by NHS‐AF488 were used. For high‐quality cytoplasmic staining and low cell density, the FISH‐quant build‐in cell profiler interface for automatic cell outline detection was used. Parameters of filtering and local focus projection were optimized per dataset. For dense cells and lower intensity cytoplasmic staining, nuclei were automatically detected in the FISH‐quant outline‐detection GUI, and cytoplasmic outlines were drawn manually. In both cases, each cell outline and nucleus was manually checked for correct segmentation before analysis. TSS were identified based on co‐localization of exon and intron signal in nuclei. After identification based on co‐localization, we defined the area of a TSS based on the exon signal in all z‐planes. In brief, according to the FISH‐quant workflow for spot detection, images were filtered, pre‐detection was performed, then spots were fitted, and fits were further thresholded to exclude outliers. For TSS detection, an average cytoplasmic spot was computed. All analysis was performed using FISH‐quant batch processing toolbox. RNA spots counts and respective localization were directly taken from FISH‐quant based analysis.

##### Quantification of bursting parameters

Bursting activity was characterized based on previously published models (Raj *et al*, 2008; Bahar Halpern *et al*, 2015b). To calculate TSS intensity, we used the FISH‐quant parameter TS_Pix_sum (sum of all pixels around brightest pixel of TSS) and the mean intensity of all quantified spots at the respective time point. While we used the kernel probability density estimate for representation of the probability density function (pdf) in figure panels, all calculations were performed based on raw data.

###### Identifying transcribing TSS

For each gene, a second smFISH probe set targeting intronic RNA regions was designed to identify actively transcribing promoters by co‐localized nuclear fluorescence signals (Fig EV2A and B). Based on the exon staining, each TSS was segmented for further analyses.

###### The correlation between burst size, frequency, and RNAs per cell

The number of RNAs per cell (*X*_RNA_) can be derived from the burst size, the burst frequency, and the RNA degradation rate (*d*_RNA_) as *X*_RNA_ = n*f*μ/*d*_RNA._

###### Fraction of active promoters (*f*) as a proxy for burst frequency

The fraction of active promoters *f* was used to approximate burst frequency, as both are correlated. It was calculated as the ratio of the number of TSS identified from co‐stained nuclear dots per cell (#TSS) and the number of genetic loci (*n*) as *f* = #TSS/*n*.

###### Correction factors for probe position and inferred RNAP2 occupancies

In line with previous work, a fixed value of 1.5 was used to correct for RNAP2 occupancies (ϰ) (Bahar Halpern *et al*, 2015b). A correction factor η for the probe position of smFISH probes was calculated based on the positioning of probes in the mRNA sequence using TransQuant software (Appendix Fig S10) (Bahar Halpern & Itzkovitz, 2016).

###### Transcription rate at active promoters (μ) as a proxy for burst size

The transcription rate at active sites (μ) was inferred from summed FIs of nascent RNAs at the TSS. For this approach, the FI of an average cytoplasmic mRNA spot is calculated from the median FI of all mature RNA spots per experiment (mInt_mRNA_) and from the relative comparison to the measured TSS intensity (Int_TSS_). Based on the summed TSS intensity values (Int_TSS_), the occupancy of RNAP2 (*M*) is calculated as the quotient of the TSS intensity and the median intensity of a cytoplasmic mRNA spot (mInt_mRNA_) as *M* = Int_TSS_/ϰ*η*mInt_mRNA_, including correction factors for the probe position (η) and inferred RNAP2 occupancies (ϰ) as described above (Bahar Halpern & Itzkovitz, 2016).

The transcription rate (μ) per hour at each TSS is then calculated as μ = M*v/*l* from the RNAP2 occupancy (*M*), the estimated RNAP2 elongation speed (*v*), and the gene length of each target (*l*) to estimate changes in burst size. Based on previous measurements in human cells, we used an RNAP elongation speed of *v* = 3 kb/min for all calculations (Fuchs *et al*, 2014). Notably, the used elongation speed affects the calculation of the transcription rate. This approach assumes an equal probability for each nucleotide position and neglects erratic transcription due to pausing or co‐transcriptional processing. The transcription rate for each cell is calculated as the number of transcribed RNAs per hour based on the RNAP2 occupancy (*M*_sum_) from all active TSS per cell as μ_sum_ = *M*_sum_*v/*l*.

###### Mean RNA degradation rate (*d*_RNA_)

Except for the RNA degradation rate (*d*_RNA_), all parameters were extracted from smFISH images. *d*_RNA_ was calculated based on *X*_RNA _= n*f*μ/*d*_RNA_. RNA lifetimes (*t*_1/2_) can be calculated as *t*_1/2 _= ln(2)/*d*_RNA_ from the decay rate (Chen *et al*, 2008), with *d*_RNA_ being the RNA degradation rate.

##### Immunofluorescence

Cells were grown on high precision coverslips #1 and fixed with 2% para‐formaldehyde, at the indicated time point after DNA damage. Subsequently, cells were permeabilized with 0.1% Triton X‐100 (Carl Roth) in phosphate‐buffered saline and blocked with 10% goat serum (PAN‐Biotech). Cells were then incubated with p53‐Fl393 for 1 h at 37°C. Cells were washed, incubated with secondary antibody coupled to Alexa Fluor 647 (Cell Signaling), and washed again. Finally, they were stained with Hoechst and embedded in Prolong Gold Antifade (Thermo Fisher Scientific). Microscopy setup was identical to the above‐mentioned description for smFISH if not described differently as follows: Images were acquired with a 20× Plan Apo objective (NA 0.75) using appropriate filter sets (Hoechst: 387/11 nm excitation [EX], 409 nm dichroic beam splitter [BS], 447/60 nm emission [EM]; Alexa Fluor 647: 640/30 nm [EX], 660 nm [BS], 690/50 nm [EM]). Images were acquired as multipoint datasets. Automated segmentation of nuclei and quantitative analysis of p53 levels based in integrated intensity of the fluorescence signal in each nucleus was performed in MATLAB (MathWorks) using custom‐written software.

##### RNA sequencing

For RNA sequencing of MCF10A cells, RNA quality was analyzed with the Agilent RNA 6000 Nano Kit, and the concentration was measured with the Qubit RNA Assay Kit (Thermo Fisher Scientific). Library preparation was carried out with the TruSeq RNA Sample Preparation Kit (Illumina) using barcoded primers. Libraries were sequenced on an Illumina HiSeq using the single read protocol (1 × 100 nt).

##### Quantitative real‐time PCR (qRT–PCR)

mRNA was extracted at the indicated time points using the High Pure RNA Isolation kit (Roche, Mannheim, Germany). cDNA was generated using M‐MuLV reverse transcriptase (NEB, Ipswich, MA) and oligo‐dT primers. Quantitative PCR was performed in triplicates using SYBR Green reagent (Applied Biosciences) on a StepOnePlus PCR machine (Thermo Fisher Scientific) or a CFX96 PCR machine (Bio‐Rad).

##### DNA FISH

DNA FISH probes for CDKN1A were amplified from genomic DNA using custom‐designed primers. Probes were labeled using DIG‐DNA labeling KIT (Roche). For detection, five probes were pooled after labeling. Before use, probes were denaturated for 10 min at 70°C and kept on ice until incubation. Cells were grown on high‐precision coverslips #1 and fixed with 2% para‐formaldehyde, washed with PBS and 2× SSC following RNAse A incubation for 2 h. Afterward a 70% formamide shock/2× SSC for 5 min was applied to reduce secondary structures and DNA was denaturated for 10 min at 80°C. Cells were rinsed in 50% formamide/2× SSC, washed with PBS, and incubated with denaturated probe for 72 h in humidified chamber sealed with rubber cement on a hybridization slide (Thermo Fisher Scientific). Afterward, cells were washed with 50% formamide/2× SSC at 42°C, 0.1% SCC at 60°C, and 4× SSC/0.1% Tween at 42°C and PBS. To detect DIG‐labeled DNA probes, anti‐DIG antibody was used. Cells were counterstained with Hoechst and mounted in Prolong Gold Antifade (Thermo Fisher Scientific). Cells were imaged on a Nikon Ti‐inverted fluorescence microscope with a ORCA R2 CCD camera (Hamamatsu), Lumen 200 Fluorescence Illumination Systems (Prior Scientific), and a 100× plan apo objective (NA 1.45) using appropriate filter sets (Hoechst: 387/11 nm excitation [EX], 409 nm dichroic beam splitter [BS], 447/60 nm emission [EM]; Alexa Fluor 647: 640/30 nm [EX], 660 nm [BS], 690/50 nm [EM]). Images were acquired as single points of 21 z‐stacks of each cell (field of view) with 300 nm step‐width using Nikon Elements software. Images of CDKN1A in Appendix Fig S9 were median‐filtered, self‐subtracted, maximum‐projected, and overlayed with nuclear (Hoechst) staining for visualization purposes using FiJi (Schindelin *et al*, 2012). For all other p53 targets, commercially available DNA FISH probes from the human RP11 BAC library were used. BAX (RP11‐29G15), SESN1 (RP11‐26B10), DDB2 (RP11‐601F23) and MDM2 (RP11‐1137N1) BAC probes were purchased from Empire Genomics labeled with 5‐ROX fluorescent dye, and staining was performed based on manufacturers protocols. BAX Images were taken as 300 nm step‐width z‐stacks with 100× objective (NA 1.4) on a Nikon Ti‐inverted microscope as described above but with an EMCCD camera (ANDOR, DU iXON Ultra 888) for BAX. For SESN1, DDB2, and MDM2, images were acquired as 300 nm step‐width z‐stacks with 60× objective (NA 1.4) on a Leica SP8 confocal microscope with 590 nm laser excitation and detection gating from 610 to 650 nm. For visualization in images (Appendix Fig S9), cells in focus were maximum‐projected and brightness‐ and contrast‐enhanced.

##### Chromatin immunoprecipitation (ChIP)

1.6 × 10^7^ cells per condition were washed once with PBS and crosslinked with 1% formaldehyde in PBS for 10 min. Cells were rinsed with cold PBS and the fixation was stopped using 125 mM Glycine in PBS for 5 min. Cells were washed with cold PBS and harvested in PBS supplemented with 1 mM PMSF. The cell pellet was resuspended in Lysis buffer (5 mM Tris–HCl, pH 8.0, 85 mM KCl, 0.5% Igepal‐CA630 supplemented with protease inhibitor cocktail from Roth and 1 mM PMSF) and incubated on ice for 20 min. The nuclear pellet was collected by centrifugation, resuspended in Sonication buffer (50 mM Tris–HCl, pH 8.1, 0.3% SDS [w/v], 10 mM EDTA supplemented with 1 mM PMSF and Protease Inhibitor Cocktail), and incubated for 30 min on ice. Chromatin was sonicated using the Covaris S220 Sonicator (PIP 105, Duty Factor 2%, CPB 200, 2 min). The sonicated samples were centrifuged and the supernatant collected. Eighty microgram of chromatin was diluted with dilution buffer (16.7 mM Tris–HCl, 167 mM NaCl, 0.01% SDS [w/v], 1.2 mM EDTA, 1.1% Triton [v/v], 1 mM PMSF, Protease Inhibitor Cocktail) and incubated overnight at 4°C with 5 μg p53 antibody (FL‐393, Santa Cruz) or a control IgG (Normal rabbit IgG, EMD Millipore). To collect the immunocomplexes, 25 μl of Dynabeads Protein G (Thermo Fisher Scientific) was added for 2 h at 4 °C. The beads were washed once with low salt washing buffer (0.1% SDS [w/v], 2 mM EDTA, 20 mM Tris–HCl pH 8.1, 1% Triton X‐100 [v/v], and 150 mM NaCl), high salt washing buffer (0.1% SDS [w/v], 2 mM EDTA, 20 mM Tris–HCl pH 8.1, 1% Triton X‐100 [v/v], and 500 mM NaCl) and LiCl washing buffer (10 mM Tris–HCl pH 8.1, 1 mM EDTA, 1% IGEPAL CA630 [v/v], 1% deoxycholic acid [w/v], 250 mM LiCl), and TE‐Buffer (10 mM Tris–HCl pH 8.1, 1 mM EDTA). The DNA was eluted from the beads for 30 min at 37 °C in Elution buffer (1% SDS, 0.1 M NaHCO_3_) twice. Crosslinks were reversed by adding 200 mM NaCl and by subsequent incubation at 65°C overnight. Fifty microgram per milliliter RNase A was added for 30 min at 37°C, then 100 μg/ml Proteinase K, 10 mM EDTA, and 40 mM Tris–HCl ph 6.5 were added and the samples were incubated for 3 h at 45°C. The DNA was cleaned up using the Monarch^®^ PCR & DNA Cleanup Kit (NEB). For qPCR, 3 μl of each sample was used.

##### Western blot and immunodetection

Cells were plated 2 days before experiments in 6‐cm dishes at 5 × 10^5^ cell density. After IR, we harvested cells at indicated time points and isolated proteins by lysis in the presence of protease and phosphatase inhibitors (Roth and Sigma‐Aldrich), Trichostatin A (APExBio), and deacetylase inhibitor cocktail (MedChemExpress). Total protein concentrations were measured by Bradford assay (Roth). Equal amounts of protein were separated by electrophoreses on NuPAGE 4–12% Bis–Tris (Invitrogen) or self‐made 10% acrylamide gels and transferred to PVDF membranes (GE Healthcare) by electroblotting (Bio‐Rad). We blocked membranes with 5% bovine serum albumin and incubated them overnight with primary antibody. The next day, membranes were washed, incubated with secondary antibody coupled to peroxidase, washed again, and protein levels were determined using chemiluminescence (Western Bright Quantum, Advansta). For detection of p53 acetylation, all blocking, wash and incubation buffers contained TSA. Precision Plus Protein Dual Color Standards (Bio‐Rad) was used for molecular mass comparison. GAPDH and acetylated p53 were detected on the same membrane. The antibodies were stripped to detect total p53 levels. Used antibodies were as follows: anti‐GAPDH (Sigma‐Aldrich, G9545), anti‐p53 (Santa Cruz #DO1, Cell Signaling, #9282), anti‐p53K70ac (Abcam, ab183544), anti‐p53K382ac (Abcam, ab75754), goat anti‐rabbit‐HRP (Thermo Fisher Scientific), goat anti‐mouse‐HRP (Thermo Fisher Scientific). Blots for p53 acetylation upon Nutlin‐3 and BML‐277 were quantified by densitometry using FIJI software (Schindelin *et al*, 2012). Measurements for total p53, p53‐K370ac, and K382ac were first individually normalized to the corresponding measurement at 3 h after irradiation, before the ratio of total and modified p53 was calculated. Mean ratios from three biological replicates are presented; error bars reflect the propagated standard error.

##### Data visualization

In boxplots, lines indicate medians of distributions; boxes include data between the 25^th^ and 75^th^ percentiles; whiskers extend to maximum values within 1.5× the interquartile range. Notches represent 5% confidence intervals for the median. Outliers are not displayed. To show single‐cell distributions of RNA spots as well as transcription rates at different time points in the same plot and allow better comparison, probability density estimates are shown instead of histograms. Data were fitted using the MATLAB function ksdensity that is a Kernel smoothing function to estimate univariate and bivariate data and returns a probability density estimate based on a normal kernel function. The kernel probability density estimate is a nonparametric representation of the probability density function (pdf) and integrates to one. PDF were only used to visualize data, all calculations were performed based on raw data. For comparison with density functions in main Figure panels, we also fitted the distribution smoothing function to histograms using the MATLAB function *histfit*, e.g., for representing RNA counts per cell. For image visualization in Figure panels, selected images of individual cells were extracted from raw data, maximum‐projected, median‐filtered and contrast‐enhanced, as described in the respective figure captions. All analysis was performed on raw data. Stacked bar graphs of the percentage of cells with active TSS were generated by binning cellular TSS activity into strongly active (> 75% of TSS) and weakly active (< 75% of TSS) cells.

## Author contributions

AL and DF designed experiments and conceived the study. DF performed experiments, analyzed data, and prepared figures. LF performed ChIP and Western blot experiments, AF RNA‐seq, and qPCR experiments. SP and AH provided substantial resources and helped with interpreting data. DF and AL wrote the manuscript with contributions from all authors. AL supervised the research.

## Conflict of interest

The authors declare that they have no conflict of interest.

## Supporting information



Appendix

Expanded View Figures PDF

Code EV1

Dataset EV1

Dataset EV2

Review Process File

Source Data for Figure 1

Source Data for Figure 2

Source Data for Figure 3

Source Data for Figure 4

Source Data for Figure 5

## Data Availability

The quantitative single‐cell measurements presented in the main and EV figures are provided as figure source data. Please note that the sorting in different source data files is not identical, i.e., data points cannot directly be related. Analysis scripts are available as Code EV1.
