# Supplementary material for: Stochastic transcription in the p53‐mediated response to DNA damage is modulated by burst frequency
Source: Mol Syst Biol. 2019 Dec 2;15(12):e9068. doi: 10.15252/msb.20199068 (PMC6886302; doi:10.15252/msb.20199068)
Supplement: Supplementary file 1 — Appendix [file MSB-15-e9068-s001.pdf]

Appendix: Stochastic transcription in the p53-mediated response to DNA damage is modulated by burst frequency.

Appendix Fig S1: RNA expression fold-change of the selected panel of p53 target genes in RNA-Seq experiments. 2

Appendix Fig S2: Nuclear p53 dynamics after 10 Gy IR in fluorescent reporter cells and wild-type A549 cells measured by live cell time-lapse microscopy and immunofluorescence. 3

Appendix Fig S3: Quantification of biological replicates of MDM2 smFISH with different sample sizes. 4

Appendix Fig S4: Histograms and fitted probability density functions of RNA counts per cell....................5

Appendix Fig S5: Fano factor of nuclear and cytoplasmic RNA levels....................6

Appendix Fig S6: Variability of RNA distributions normalized by nuclear and cell area....................7

Appendix Fig S7: Ratios of nuclear to cytoplasmic percentiles of p53 targets remain largely constant upon IR. 8

Appendix Fig S8: Number of quantified TSS in the population in basal condition and after DNA damage. .9

Appendix Fig S9: DNA FISH to confirm the number of genomic loci in A549 cells....................10

Appendix Fig S10: Probe position along the nascent RNA and correction factor for probe position from Trans Quant. 11

Appendix Fig S11: H3K27ac and H3K27me ChIP at p53 target gene promoters after IR....................12

Appendix Fig S12: Western Blot repeat experiments....................13

Appendix References 14

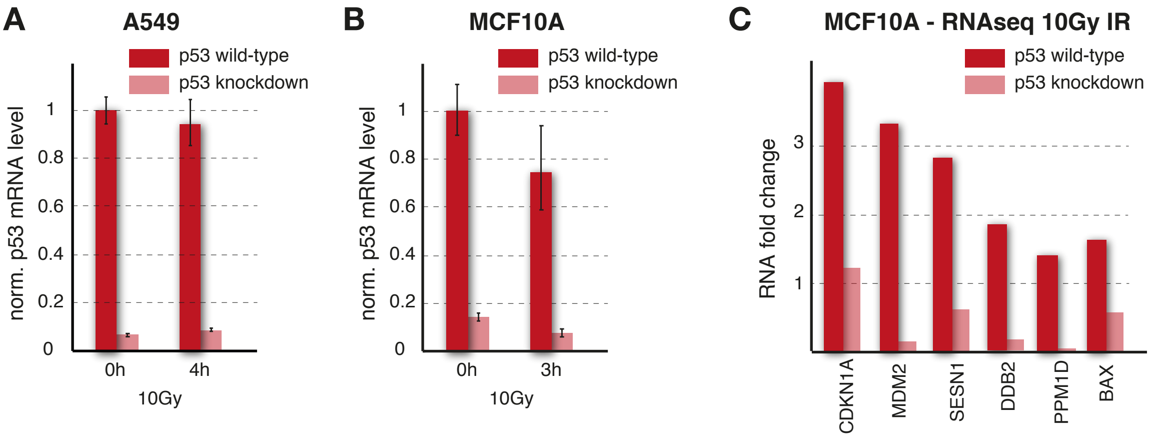

D

	MCF10A			MCF7 (Hafner 2017)			A549		
		0 h (basal)	4 h 10 Gy		0 h (basal)	4 h 10 Gy		0h (basal)	3 h 10 Gy
	fc	RPKM	RPKM	fc	FPKM	FPKM	fc	RNA counts	RNA counts
**BAX**	1.8	50.6	89.0	2.2	103.7	224.8	1.5	103.0	168.0
**CDKN1A**	3.9	62.5	246.2	14.4	35.6	514.2	1.6	103.0	195.0
**DDB2**	1.9	24.8	47.6	3.6	34.2	123.6	2.6	14.0	37.0
**MDM2**	3.3	15.7	51.6	3.4	38.1	127.9	2.3	106.0	261.0
**PPM1D**	1.9	2.1	4.1	2.0	56.3	110.8	6.8	8.0	61.0
**SESN1**	3.0	5.2	15.6	5.9	11.5	67.8	1.4	22.0	31.0

Appendix Fig S1: RNA expression fold-change of the selected panel of p53 target genes in RNA-Seq experiments.

A Relative p53 RNA expression levels at basal state and 3 h after 10 Gy IR in p53 wild-type (dark red) and p53 knockdown (light red) A549 cells measured by qRT-PCR.

B Relative p53 RNA expression levels at basal state and 3 h after 10 Gy IR in p53 wild-type cells (dark red) and p53 knockdown (light red) MCF10A cells measured by qRT-PCR.

C Bargraphs of the expression fold change 4 h after 10 Gy IR compared to basal state in wild-type and p53 knockdown MCF10A cells measure by RNA-Seq.

D The fold changes of expression of the selected panel of p53 target genes have been analyzed after 10 Gy IR in previous studies. The overview table shows average RNA levels quantified in three different cell lines: A549, MCF7 and MCF10A by RNA-Seq (MCF7 and MCF10A) and smFISH (A549). Fc is the fold change of induction and was calculated from the change in read counts (RNA-Seq) or smFISH spots (A549) measured before DNA damage and 3 h or 4 h after 10 Gy IR. Data for MCF7 cells was extracted from a recent publication by Hafner et al. (Hafner et al, 2017). To give an approximate of the expression strength, read counts from RNA-Seq are listed for both IR treated cell lines as well. Gy: Gray; RPKM: reads per kilo base per million mapped reads; FPKM: fragments per kilo base per million reads.

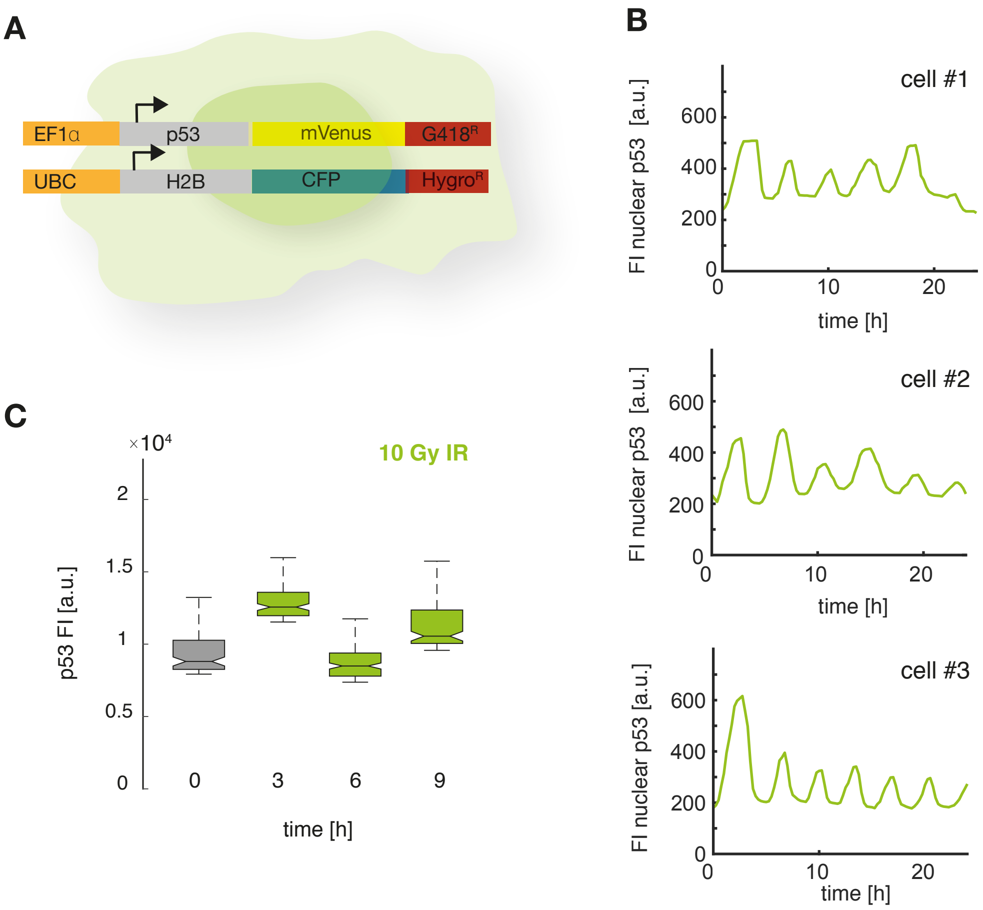

Appendix Fig S2: Nuclear p53 dynamics after 10 Gy IR in fluorescent reporter cells and wild-type A549 cells measured by live cell time-lapse microscopy and immunofluorescence.

A Live cell time lapse imaging of transgenic A549 reporter cell lines allows to validate synchronous nuclear pulsing and define time points of repeated pulses of nuclear accumulation of p53 upon DNA damage by 10 Gy IR. A schematic representation of the p53-mVenus, H2B-CFP reporter cell line used to validate time point for smFISH measurements is shown. p53-mVenus is constitutively expressed from a human EF1a-promoter, a Neomycine resistance gene is expressed for antibiotic selection (G418). H2B-CFP is constitutively expressed from a human Ubiquitine-C promoter, a Hygromycine resistance gene is expressed for antibiotic selection(Hygro).

B Representative examples of three quantitative trajectories from 24 h live cell time lapse microscopy imaging of A549 reporter cells after 10Gy IR (See Methods section for details on reporter and quantification).

C Automated quantification of p53 levels in A549 wild-type cells based on immunofluorescence experiments. Quantified as integrated intensity of p53-AF647 (FI a.u.) antibody staining in nuclear area of A549 wild type cells before (basal) and 3h, 6h, 9h after 10 Gray of IR. Quantification of integrated intensity in cells with the highest 10% of signal (see Data Visualization section). Sample size of the total dataset is 4839 cells (quantified nuclei), the upper 10th percentile of each condition is represented by 482 cells in total with similar sample sizes of n >100 for all conditions.

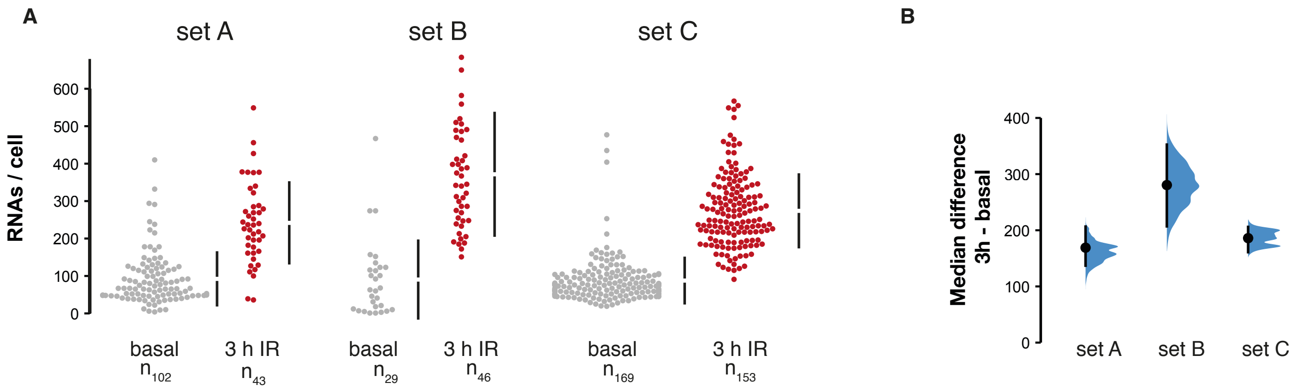

Appendix Fig S3: Quantification of biological replicates of MDM2 smFISH with different sample sizes.

A Estimation plot comparison of three datasets of MDM2 smFISH with different sample sizes. A shows the RNA raw data of RNA counts per cell in basal state and 3 h after 10 Gy IR in three biological replicates (set A,B,C) with different sample sizes (n=29 - n=169).

B Plot of median difference as bootstrap sampling distribution of 5000 bootstrap samples. Median differences are shown as dots; 95% confidence intervals are indicated by the ends of the vertical error bars. The effect sizes and CIs as effect size [CI width lower bound; upper bound]: The unpaired median difference between basal (set A) and 3 h (set A) is 1.69e+02 [95.0%CI 1.37e+02, 2.06e+02]. The two-sided P value of the Kruskal test is 2.31e-13. The unpaired median difference between basal (set B) and 3 h (set B) is 2.8e+02 [95.0%CI 2.07e+02, 3.52e+02]. The two-sided P value of the Kruskal test is 5.36e-11. The unpaired median difference between basal (set C) and 3 h (set C) is 1.86e+02 [95.0%CI 1.61e+02, 2.06e+02]. The two-sided P value of the Kruskal test is 5.51e-48. Calculations were performed using estimation statistic software tools provided by Ho et al. (Ho et al, 2019).

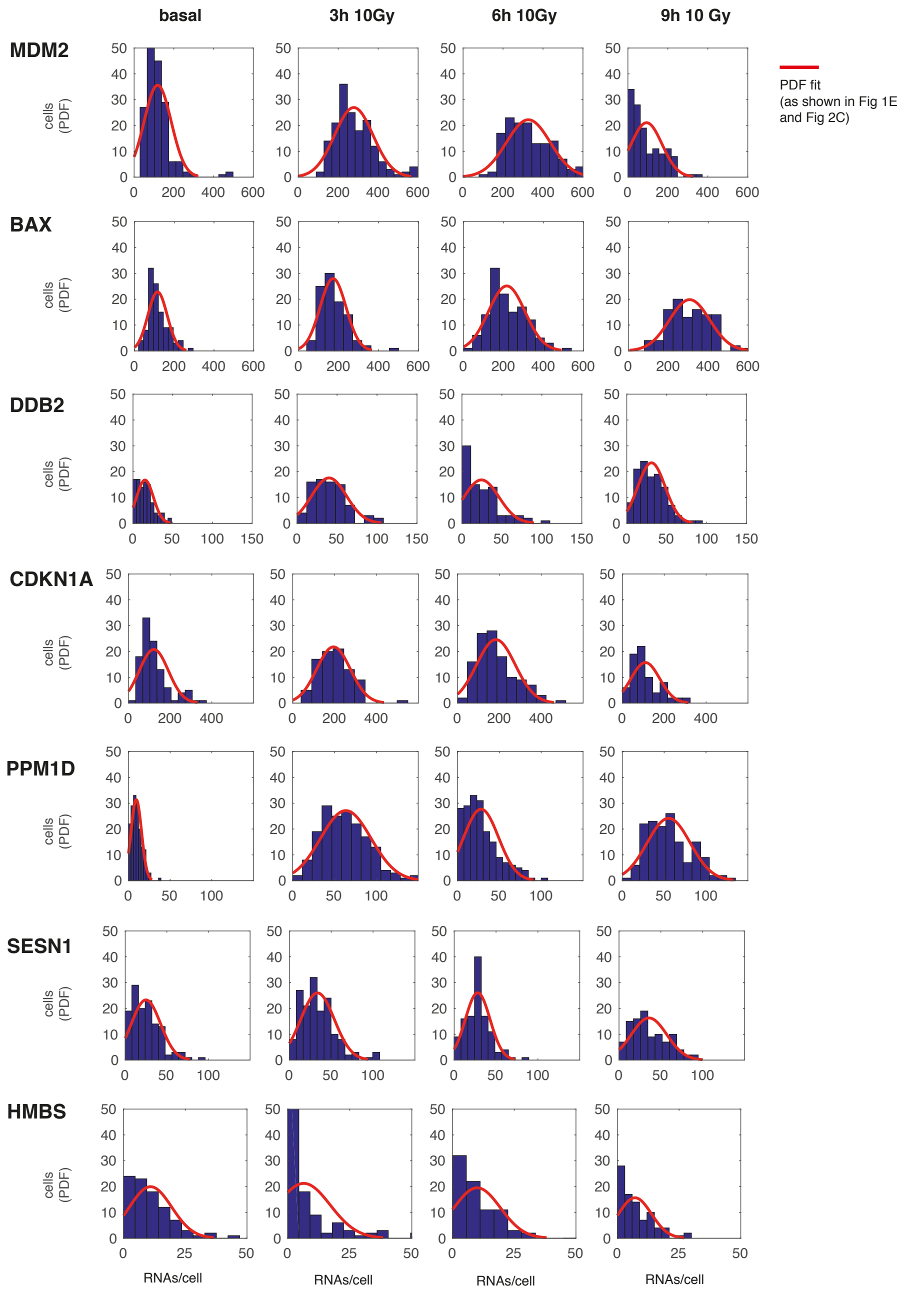

Appendix Fig S4: Histograms and fitted probability density functions of RNA counts per cell.

Histograms and probability density estimates (red line) as shown in Figure 2C of total RNAs/cell at each time point for all p53 target genes are plotted for comparison of raw data to fits. As described in the Methods section the Matlab function „*histfit*" was used to fit pdf to data (see Data Visualization section).

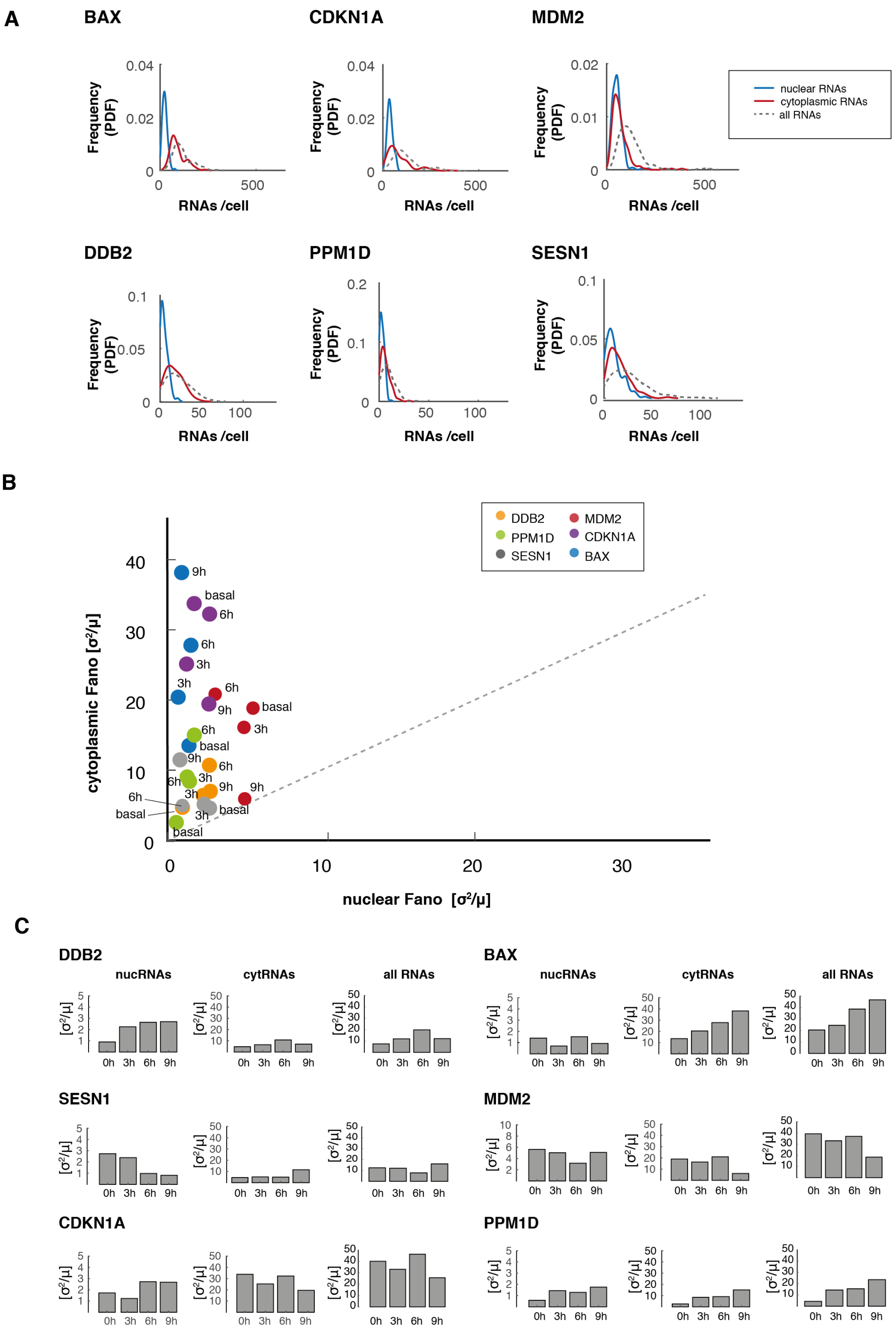

Appendix Fig S5: Fano factor of nuclear and cytoplasmic RNA levels.

A Probability density estimates (PDF) of RNA dispersions in nucleus (blue) and cytoplasm (red) compared to all cells (dashed line, grey) in basal conditions show rather increased noise in cytoplasm compared to nucleus. This indicates an either similar noise state or an amplification of gene expression noise from the nucleus to the cytoplasm. RNAs were plotted from nuclear or cytoplasmic fractions for all p53 target genes in our panel.

B We calculated the Fano factor of RNA counts in the nuclear and cytoplasmic fraction of cells for all p53 targets as a measure of noise strength of gene expression over time after DNA damage (10Gy IR).. Data points are labeled, time points are visualized by the indicated color code. The diagonal is shown as a guide to the eye (dashed line).

C After DNA damage, changes in noise strength of gene expression as measured by Fano factors are gene specific. Fano factors for nuclear (left) and cytoplasmic (middle) and total RNAs (right panel) are shown for the indicated target genes and time-points. Cytoplasmic fractions in general show higher noise levels, but both sub-cellular regions seem to undergo specific changes in noise that do not necessarily follow the same trends. In noise measurements of total RNAs the distinct nuclear RNA noise levels are masked.

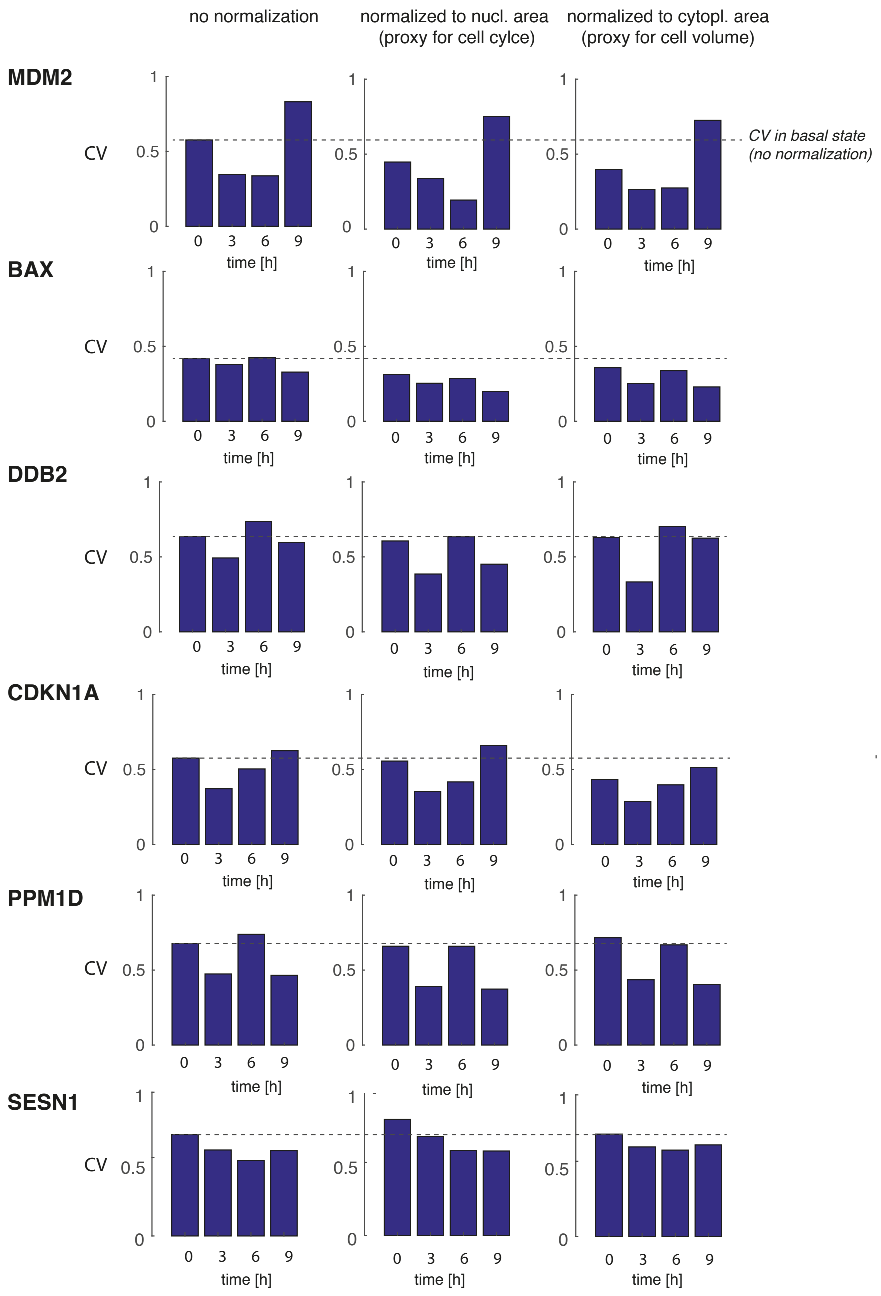

Appendix Fig S6: Variability of RNA distributions normalized by nuclear and cell area.

Variability in the distribution of mRNAs was measured as Coefficient of Variation (CV). Data from raw measurements (left panel), counts normalized by nuclear area as a proxy for cell cycle (middle panel) and counts normalized by cell area from cytoplasmic staining as a proxy for cell volume (right panel) is shown . For all quantifications, relative changes from median size was factorized into RNA counts before CV calculations. Dashed line shows CV at basal state w/o normalization.

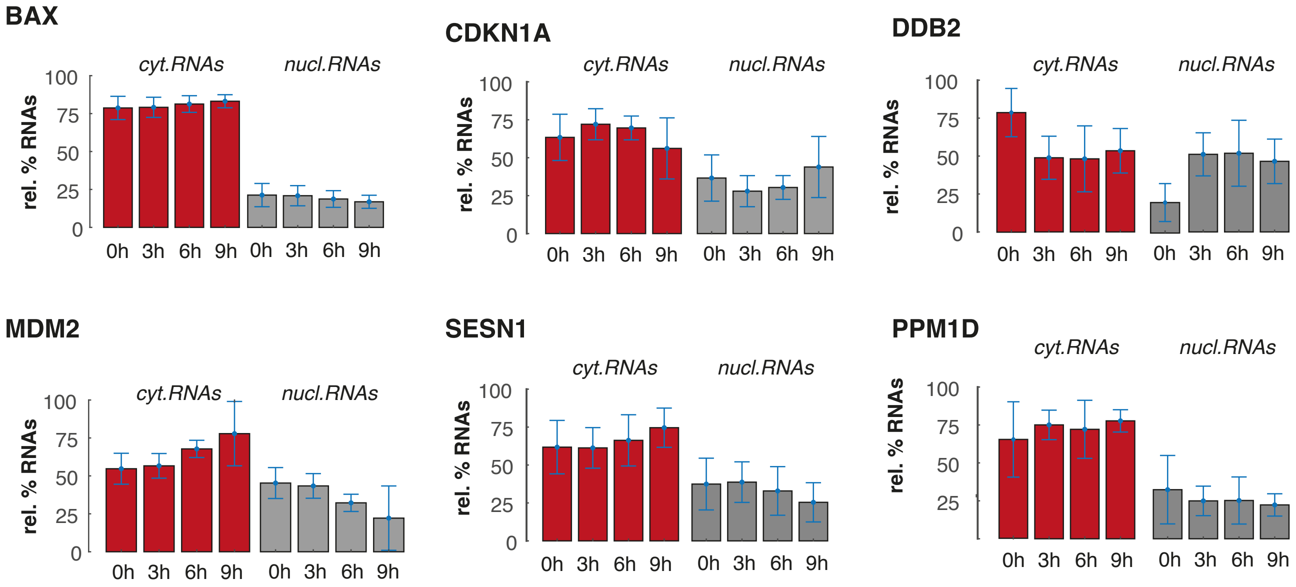

Appendix Fig S7: Ratios of nuclear to cytoplasmic percentiles of p53 targets remain largely constant upon IR.

Relative quantification of the ratios of nuclear to cytoplasmic percentiles in single cells over time after DNA damage for all p53 target genes. RNA counts in the nucleus and the cytoplasm have been quantified using FISH-quant software (Mueller et al, 2013), sub-cellular localization was defined based on Hoechst and NHS-488 staining. The relative percentage of RNAs in nucleus and cytoplasm was calculated and compared in the population. Red bars depict the percentage of cytoplasmic RNAs, grey bars of RNAs in nucleus. Error bars represent standard deviation (SD) of the calculated ratio, the sample size (n) for each dataset can be found in Fig 2B.

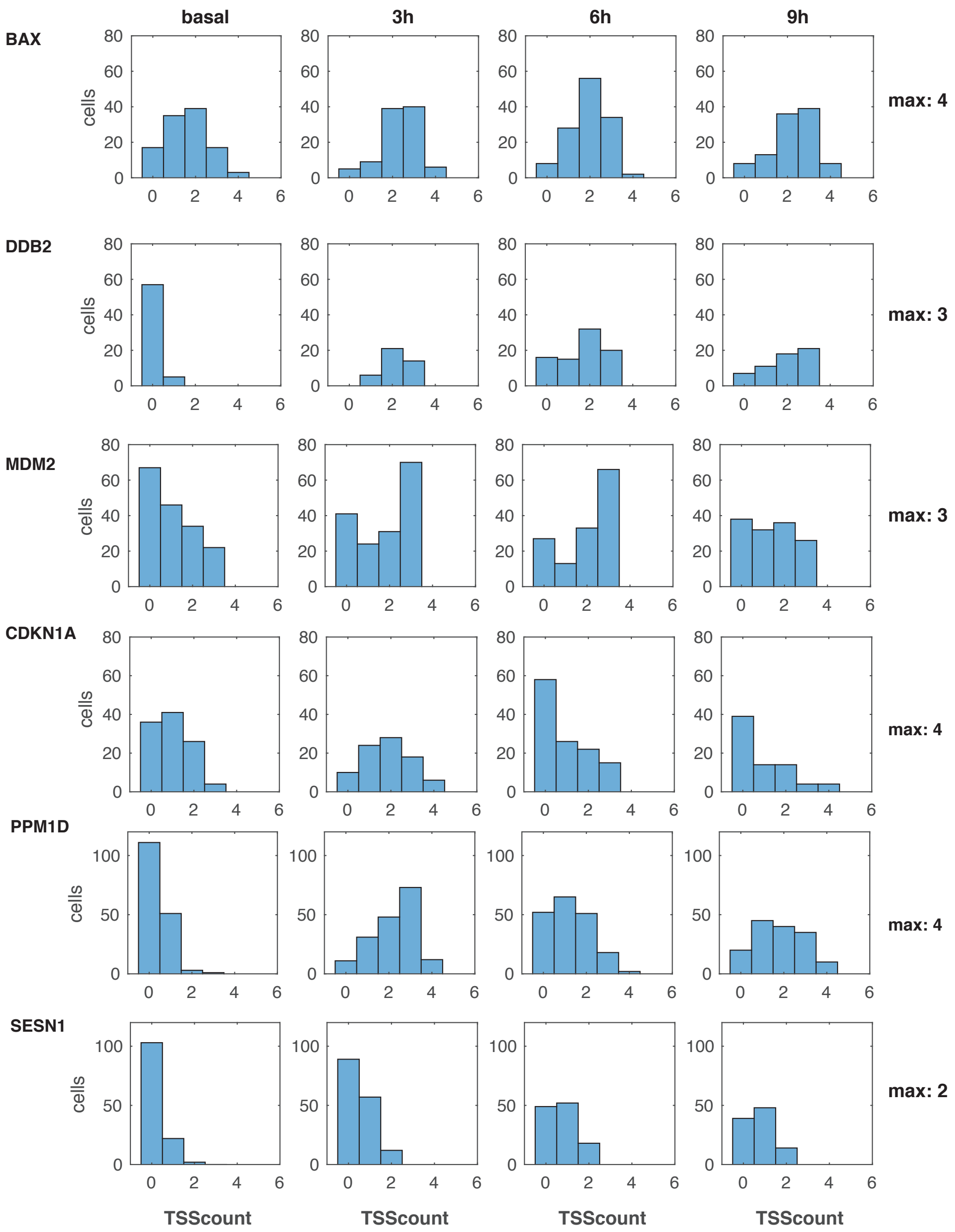

Appendix Fig S8: Number of quantified TSS in the population in basal condition and after DNA damage.

Histograms show the distribution of identified TSS per cell for all p53 target genes at the measurement time-points identified from co-localization of intron-exon staining. The sample size is as indicated in Fig 2/3. The identified TSS numbers per cell ranged between 2 (SESN1) and 4 (BAX). Most p53 target genes had more than two TSS that were detected from co-stained nuclear smFISH spots in A549 cells.

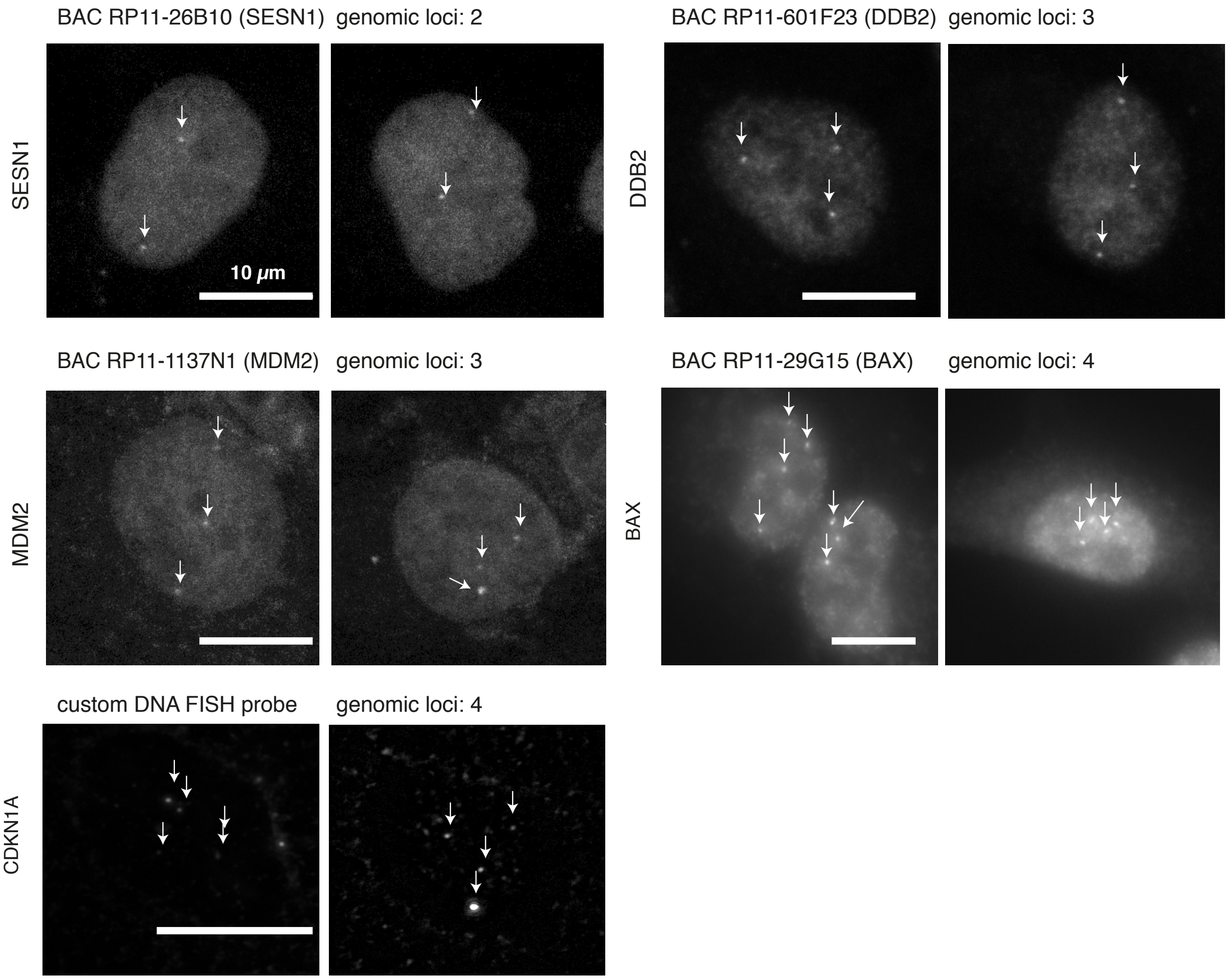

Appendix Fig S9: DNA FISH to confirm the number of genomic loci in A549 cells

Images of fluorescent labeling of genomic loci by DNA FISH to confirm the number of maximum TSS as estimated in Appendix Fig S8. DNA FISH for the different target genes has been performed with different probes and microscopy set ups (see methods section). Arrows indicate genomic loci, scale bars correspond to 10 µm. DNA FISH measurements confirmed 2 loci for SESN1, 3 loci for MDM2 and DDB2 and 4 loci for BAX and CDKN1A. For PPM1D neither a custom designed probe amplified from genomic DNA nor an RP11 library based commercial BAC probe provided suitable signals. However, as all other DNA FISH experiments confirmed the number of identified TSS from smFISH co-staining, we assumed that intron-exon co-staining by smFISH can be used reliable to identify TSS.

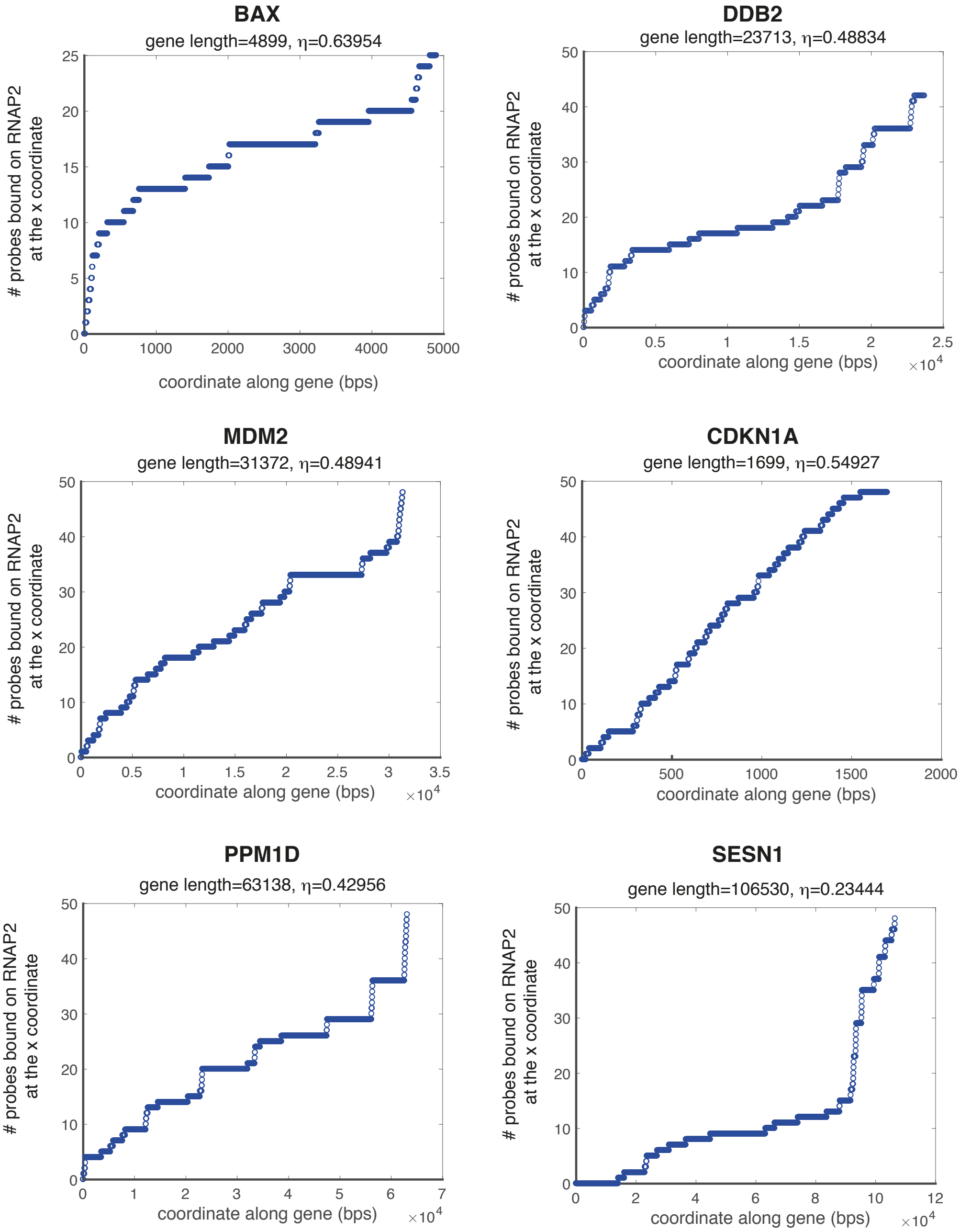

Appendix Fig S10: Probe position along the nascent RNA and correction factor for probe position from *Trans Quant.*

To correct for the positioning of smFISH oligos along each target gene, we included a correction factor for probe position into our calculations of RNAP2 occupancy and thus transcription rate as suggested by Bahar Halpern et al. (Bahar Halpern et al, 2015). To calculate this correction factor we used *Trans Quant* software, providing the sequences of target genes and smFISH oligos (Bahar Halpern & Itzkovitz, 2016).

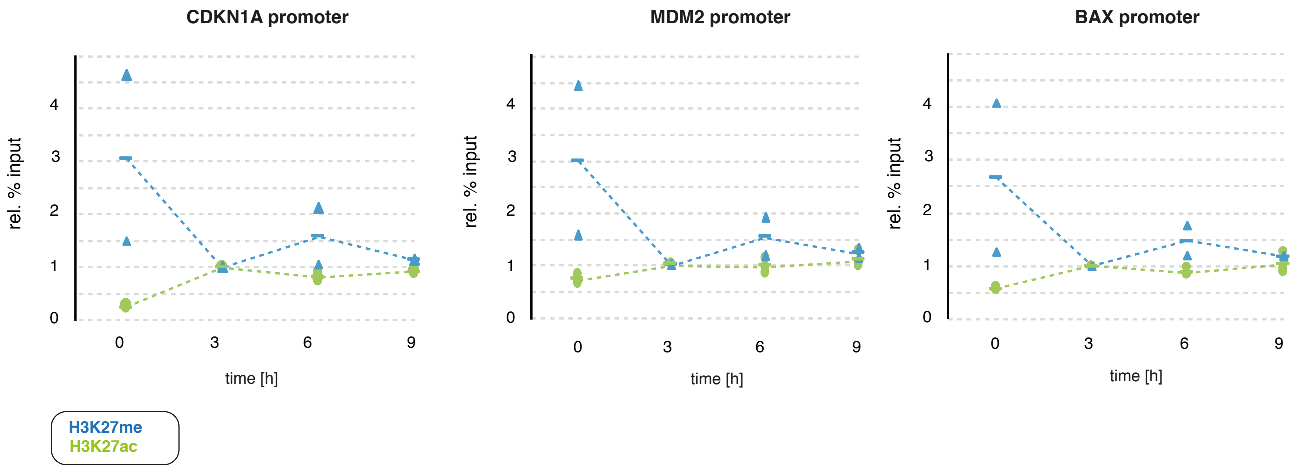

Appendix Fig S11: H3K27ac and H3K27me ChIP at p53 target gene promoters after IR.

ChIP experiment to measure the relative fraction of H3K27ac (green) and H3K27me (blue) after DNA damage at indicated promoters. The amount of modified histones was calculated as percentage of input and normalized to the time-point of the first p53 peak at 3 h. Individual data points (mean values of triplicate quantification in qRT-PCR measurements) from 2 biological repeats are shown as dots, mean values are displayed as horizontal lines. Dashed lines serve as guide to the eyes.

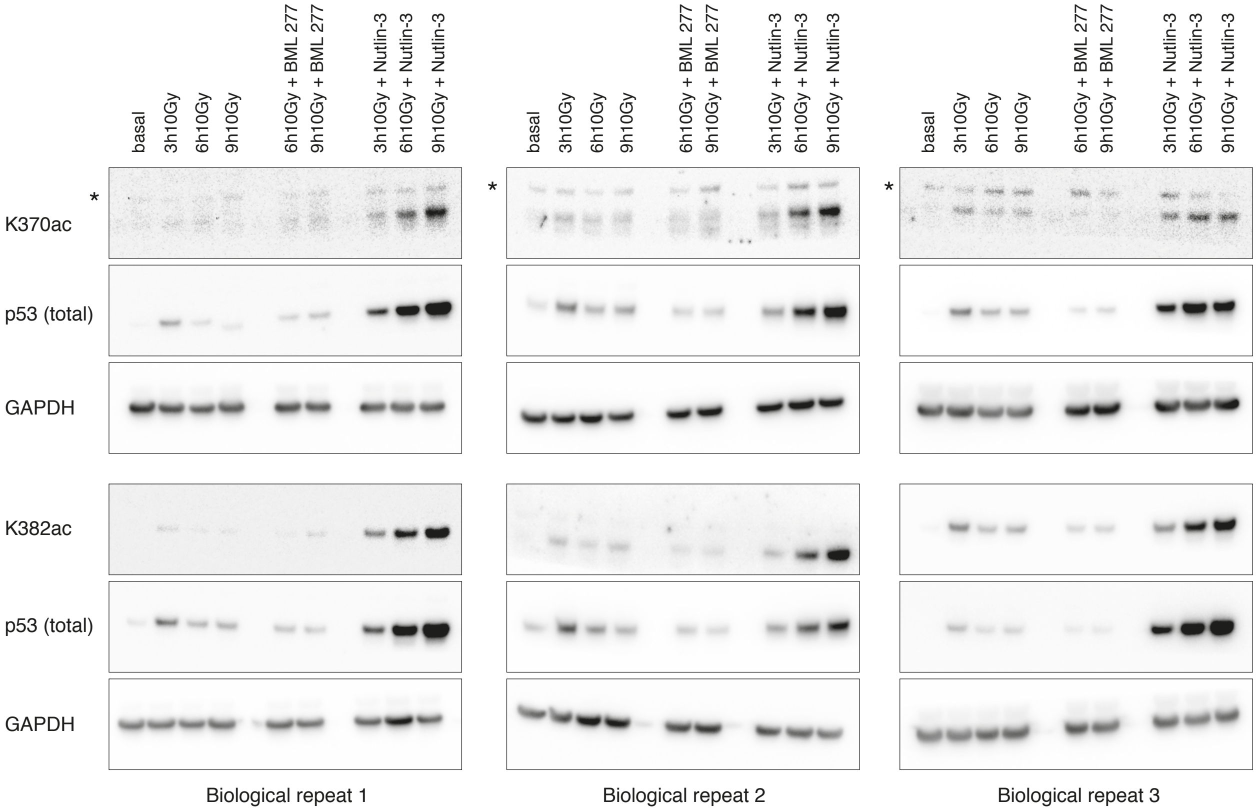

Appendix Fig S12: Western Blot repeat experiments

Total p53, p53 acetylated at K382 and K370 as well as GAPDH were measured by Western Blot at indicated time points in the context of different p53 dynamics: pulsing p53 (10 Gy IR), transient p53 (10 Gy IR + BML-277, central lanes) and sustained p53 (10 Gy IR + Nutlin-3, right lanes). Three biological replicates that have been used for the quantification in Fig 5C using FIJI (Schindelin et al, 2012) are shown. Asterix indicates unspecific bands.

Appendix References

Bahar Halpern K, Itzkovitz S (2016) Single molecule approaches for quantifying transcription and degradation rates in intact mammalian tissues. *Methods* **98:** 134-142

Bahar Halpern K, Tanami S, Landen S, Chapal M, Szlak L, Hutzler A, Nizhberg A, Itzkovitz S (2015) Bursty gene expression in the intact mammalian liver. *Mol Cell* **58:** 147-156

Hafner A, Stewart-Ornstein J, Purvis JE, Forrester WC, Bulyk ML, Lahav G (2017) p53 pulses lead to distinct patterns of gene expression albeit similar DNA-binding dynamics. *Nat Struct Mol Biol* **24:** 840-847

Ho J, Tumkaya T, Aryal S, Choi H, Claridge-Chang A (2019) Moving beyond P values: data analysis with estimation graphics. *Nat Methods* **16:** 565-566

Mueller F, Senecal A, Tantale K, Marie-Nelly H, Ly N, Collin O, Basyuk E, Bertrand E, Darzacq X, Zimmer C (2013) FISH-quant: automatic counting of transcripts in 3D FISH images. *Nat Methods* **10:** 277-278

Schindelin J, Arganda-Carreras I, Frise E, Kaynig V, Longair M, Pietzsch T, Preibisch S, Rueden C, Saalfeld S, Schmid B, Tinevez JY, White DJ, Hartenstein V, Eliceiri K, Tomancak P, Cardona A (2012) Fiji: an open-source platform for biological-image analysis. *Nat Methods* **9:** 676-682
