# Supplementary material for: Stochastic transcription in the p53‐mediated response to DNA damage is modulated by burst frequency
Source: Mol Syst Biol. 2019 Dec 2;15(12):e9068. doi: 10.15252/msb.20199068 (PMC6886302; doi:10.15252/msb.20199068)
Supplement: Supplementary file 2 — Expanded View Figures PDF [file MSB-15-e9068-s002.pdf]

# Expanded View Figures

**Figure EV1. Noise in RNA abundance indicates stochastic bursting and a change in burst frequency after DNA damage.**

A p53 target genes have different genomic architecture and *cis*-regulatory logic. The number and position of p53 response element vary dependent on the target gene. A schematic illustration shows the relative positioning of p53 response elements (RE), transcriptional start sites (TSS), and exons (Tebaldi *et al*, 2015; Fischer, 2017). p53 REs analyzed by ChIP are marked in red. Gene length and chromosomal positions are indicated. Please note that introns and exons are not drawn to scale. Gene structure represents sum of all alternative transcripts.

B Scheme of smFISH probe design and strategy. Multicolor fluorescent imaging enables segmentation of subcellular regions based on nuclear and cytoplasmic stains that can then be used to assign RNA counts to their cells of origin and subcellular locations. From left to right: Individual images of nuclei by Hoechst-405, cytoplasmic staining by NHS-488 and smFISH exon staining by Cal Fluor 610 nm staining. Examples of outline generation for nuclear and cytoplasmic regions and spot detection on exon stainings. See Materials and Methods for details.

C The total cytoplasmic expression noise was plotted against the total nuclear expression noise (each measured as $\sigma^2/\mu^2 = CV^2$) for the indicated p53 target genes before and 3, 6, and 9 h after 10 Gy IR. Data points are labeled; time points are visualized by the indicated color code. The diagonal is shown as a guide to the eye (dashed line).

D The squared coefficient of variation ($CV^2$) in relation to mean RNA count per cell is shown for the indicated p53 target genes over time after DNA damage (10 Gy IR). Data points are labeled with target gene and time points after IR. As guides to the eye, hyperbolic lines show the expected noise scaling upon regulating the frequency of promoter activation ($CV^2 = b/\langle mRNA \rangle$ with $b = \mu/k_{on}$ (burst size, assuming an overall low frequency of promotor activity ($k_{off} \gg k_{on}$), see Fig 3A and B for an illustration of the underlying model). Noise scaling for burst sizes ranging from 5 to 70 is shown as indicated on top of the graph (Singh *et al*, 2010; Dar *et al*, 2016).

© 2019 The Authors 

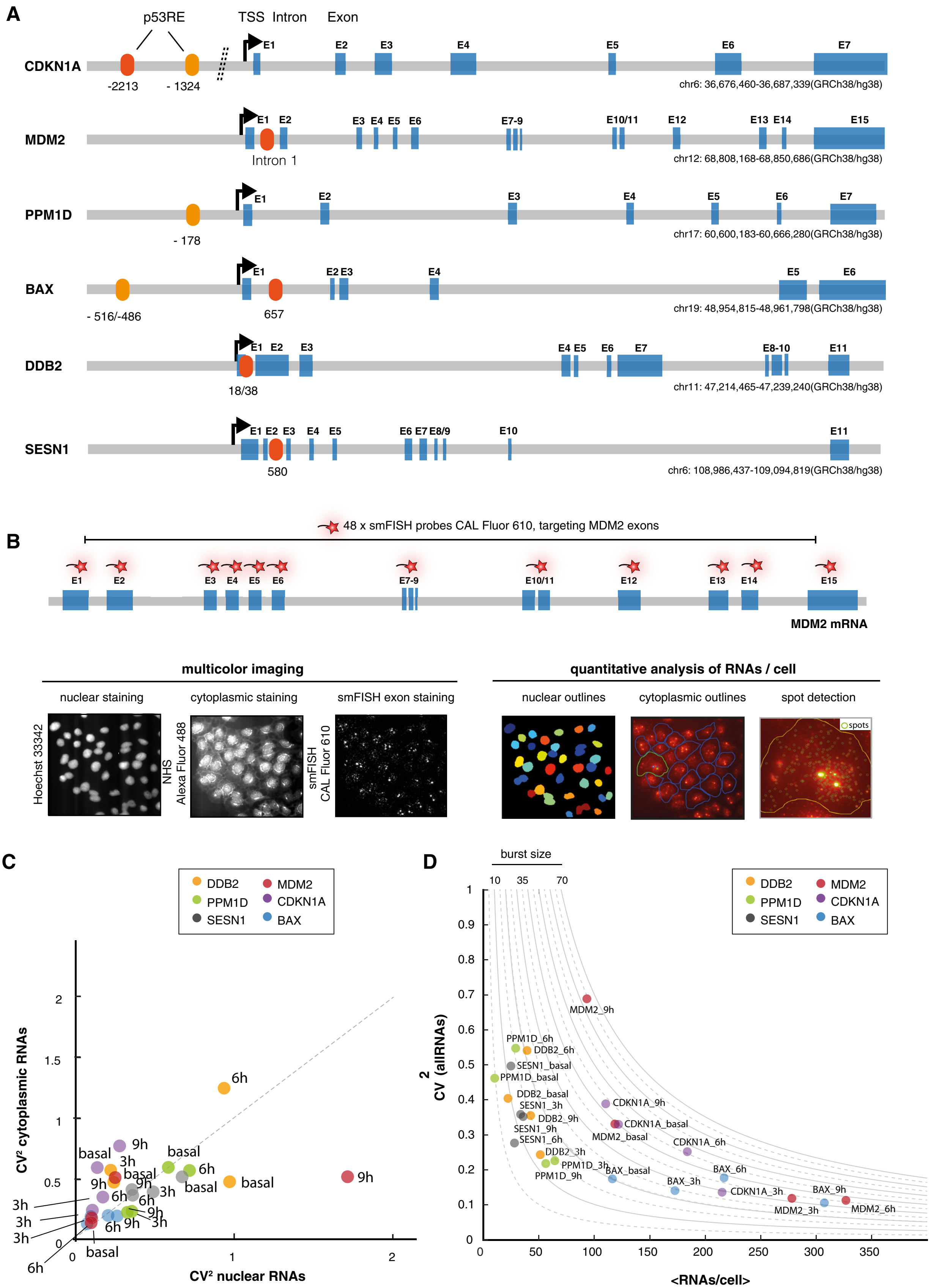


**Figure EV1.**

 © 2019 The Authors

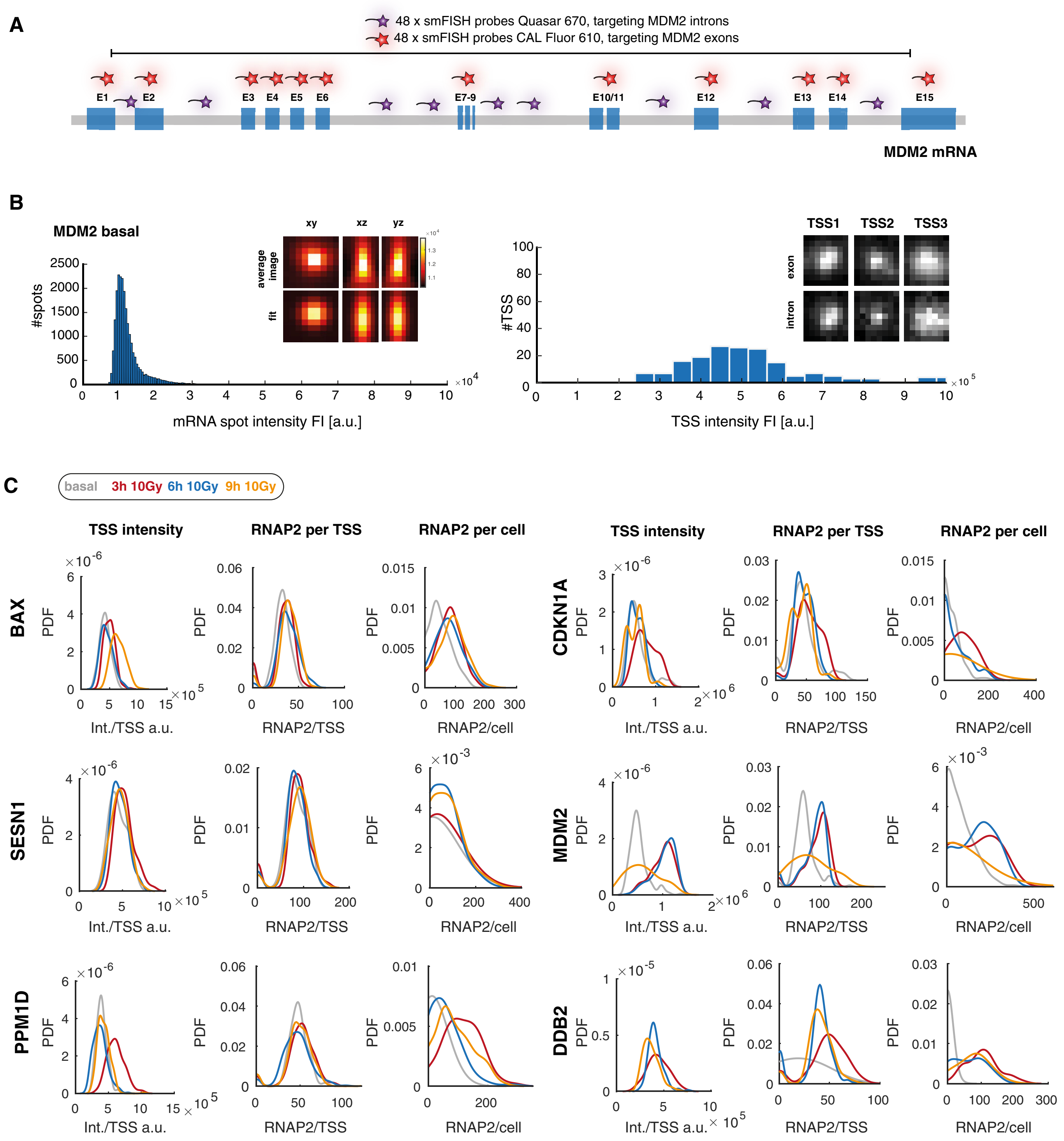
A
48 x smFISH probes Quasar 670, targeting MDM2 introns
48 x smFISH probes CAL Fluor 610, targeting MDM2 exons
E1 E2 E3 E4 E5 E6 E7-9 E10/11 E12 E13 E14 E15
MDM2 mRNA
B
MDM2 basal
#spots
mRNA spot intensity FI [a.u.]
xy xz yz
average image
fit
TSS1 TSS2 TSS3
exon
intron
#TSS
TSS intensity FI [a.u.]
C
basal 3h 10Gy 6h 10Gy 9h 10Gy
TSS intensity
RNAP2 per TSS
RNAP2 per cell
BAX
SESN1
PPM1D
CDKN1A
MDM2
DDB2
PDF
Int./TSS a.u.
RNAP2/TSS
RNAP2/cell

Figure EV2.

**Figure EV2. Calculation of RNAP2 occupancy and transcription rate from TSS intensity.**

A Schematic representation of smFISH co-staining to identify TSS. Exon probes are labeled with CAL Fluor Red 610 and intron probes with Quasar 670. The latter probes are spread over several introns.

B Left panel: Histogram of the fluorescence intensity (FI) distribution for MDM2 RNA spots in basal state as an example. Calculated images in *xy*, *xz*, and *yz* dimensions of the average MDM2 RNA spot generated by FISH-quant are depicted (upper row) as well as the corresponding fits (lower row). The FI intensity is indicated by a heat map. Right panel: Histogram showing the distribution of the FI for identified TSS transcribing MDM2 RNAs in basal state as an example. Image clippings show examples of intron and exons staining of three MDM2 TSS in basal state. For visualization, images were maximum-projected and brightness- and contrast-enhanced.

C Quantified parameters of promoter activity for the indicated target genes before (basal, gray) and 3 h (red), 6 h (blue), and 9 h (orange) after DNA damage (10 Gy IR). The left panel for each target gene shows distributions for quantified TSS intensities from FISH-quant displayed as probability density estimates (pdf) of all active TSS. Center panels indicate distributions of RNAP2 occupancies at individual TSS, right panels the RNAP2 occupancies in the whole cell as calculated from the relative intensity of a TSS and the average cytoplasmic mRNA intensity (see Materials and Methods section for details). These occupancies were used to calculate transcription rates per hour.

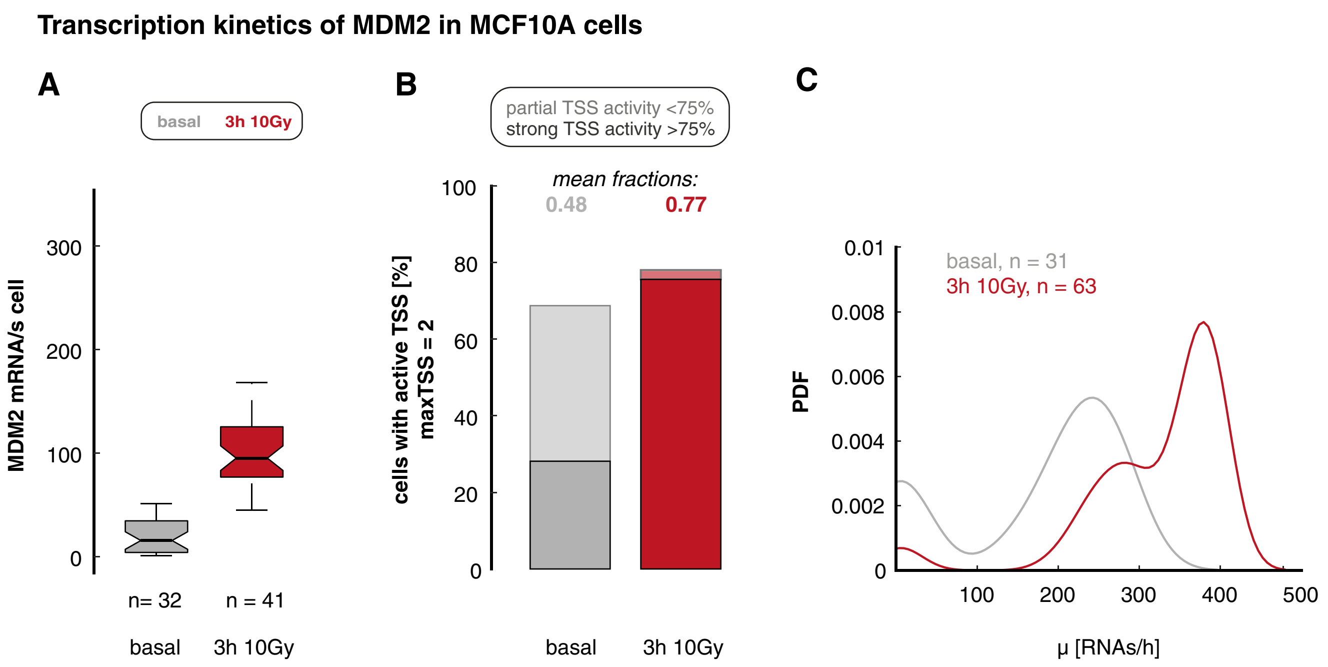


**Figure EV3. Bursting kinetics of MDM2 in MCF10A cells.**

A We quantified MDM2 RNAs per cell before (basal, gray) and 3 h (red) after DNA damage (10 Gy IR) in diploid non-transformed MCF10A cells. Boxplots show smFISH measured RNA counts from single-cell analysis of gene expression (see Data Visualization section); lines indicate medians of distributions; boxes include data between the 25$^{th}$ and 75$^{th}$ percentiles; whiskers extend to maximum values within 1.5× the interquartile range. Notches represent 5% confidence intervals for the median. The number of measured cells is indicated as *n*.

B The percentage of MCF10A cells with active MDM2 TSS, subdivided into populations with strong (> 75% of TSS, solid colors) and weak (< 75% of TSS, shaded colors) activity, is shown as stacked bar graphs; the mean fraction of active promoters is indicated above each bar. As for A549 cells, we observed an increase in the fraction of active MDM2 promoters (as a proxy for burst frequency) upon DNA damage.

C Distributions of calculated transcription rates at active MDM2 TSS are presented for each time point as probability density estimates (PDF, see Data visualization section). The transcription rates increased 3 h after DNA damage.

 © 2019 The Authors

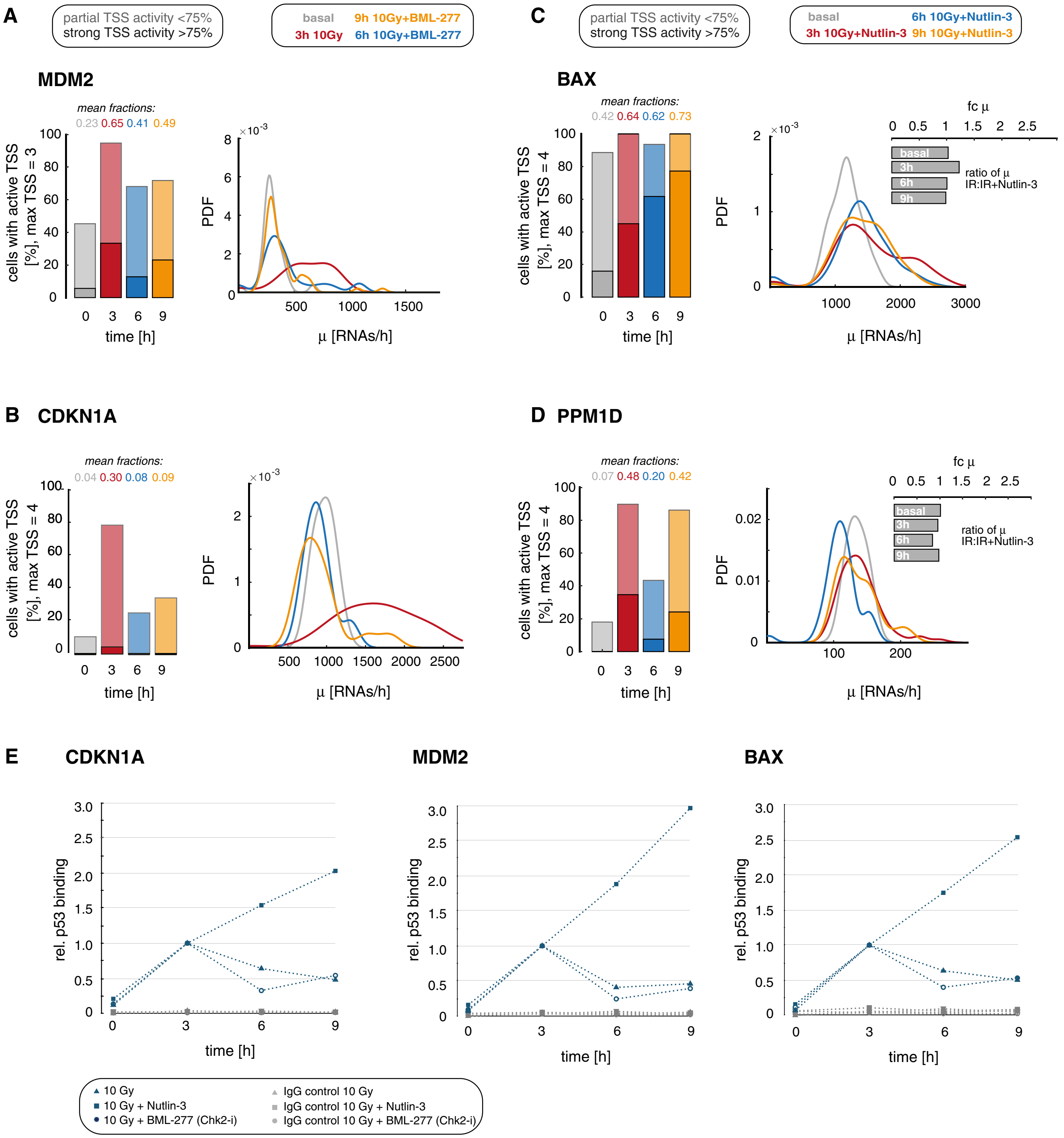
A
partial TSS activity <75%
strong TSS activity >75%
basal 9h 10Gy+BML-277
3h 10Gy 6h 10Gy+BML-277
MDM2
mean fractions:
0.23 0.65 0.41 0.49
cells with active TSS [%], max TSS = 3
time [h]
PDF
µ [RNAs/h]
C
partial TSS activity <75%
strong TSS activity >75%
basal 6h 10Gy+Nutlin-3
3h 10Gy+Nutlin-3 9h 10Gy+Nutlin-3
BAX
mean fractions:
0.42 0.64 0.62 0.73
cells with active TSS [%], max TSS = 4
fc µ
ratio of µ IR:IR+Nutlin-3
basal
3h
6h
9h
B CDKN1A
mean fractions:
0.04 0.30 0.08 0.09
D PPM1D
mean fractions:
0.07 0.48 0.20 0.42
E
CDKN1A
MDM2
BAX
rel. p53 binding
time [h]
10 Gy
10 Gy + Nutlin-3
10 Gy + BML-277 (Chk2-i)
IgG control 10 Gy
IgG control 10 Gy + Nutlin-3
IgG control 10 Gy + BML-277 (Chk2-i)

**Figure EV4.**

◀

**Figure EV4. Quantification of bursting kinetics of target genes upon modulated p53 dynamics.**

A, B We quantified promoter activity of MDM2 (A, transient archetype) and CDKN1 (B, transient archetype) before (basal, gray) and 3 h (red), 6 h (blue), and 9 h (orange) after irradiation with 10 Gy IR and inhibition of the second p53 pulse by Chk2 inhibition. Left panel: The percentage of cells with active TSS, subdivided into populations with strong (> 75% of TSS, solid colors) and weak (< 75% of TSS, shaded colors) activity, is shown as stacked bar graphs, the mean fraction of active promoters is indicated above each bar. Right panel: Distributions of calculated transcription rates at active TSS are presented for each time point as probability density estimates (PDF, see Data visualization section). As both target genes were grouped in the transient archetype, no obvious changes in promoter activity were observed.

C, D We quantified promoter activity of BAX (C, sustained archetype) and PPM1D (D, pulsatile archetype) before (basal, gray) and 3 h (red), 6 h (blue), and 9 h (orange) after irradiation with 10 Gy IR and sequential Nutlin-3 treatment. Left panel: The percentage of cells with active TSS, subdivided into populations with strong (> 75% of TSS, solid colors) and weak (< 75% of TSS, shaded colors) activity, is shown as stacked bar graphs; the mean fraction of active promoters is indicated above each bar. Right panel: Distributions of calculated transcription rates at active TSS are presented for each time point as probability density estimates (PDF, see Data visualization section). For BAX, both transcription parameters remained high. Interestingly, in the contrary to our observations for target genes that change their gene expression mode in response to Nutlin-3 treatment, the transcription rate did not change notable for BAX. Surprisingly, the same holds true for PPM1D, a gene that we assigned to the pulsatile archetype.

E To measure relative p53 binding at different target gene promoters in perturbed and unperturbed cells, we performed ChIP experiments in the context of Nutlin-3 and BML-277 treatment for all p53 target genes at the indicated time points after 10 Gy IR-CDKN1A (left), MDM2 (center), and BAX (right; we could not detect any p53 binding at the published response element in the PPM1D promoter in repeated experiments). The amount of bound p53 was calculated as percentage of input and normalized to the time point of the first p53 peak at 3 h. Gray symbols indicate the corresponding IgG control. Interestingly, pulsatile p53 and inhibition of the second p53 pulse both lead to a gradual decrease of p53 binding after the first peak, Nutlin-3 treatment increases p53 binding at these promoters and may thereby contribute to the observed increase in promoter activity.

 © 2019 The Authors

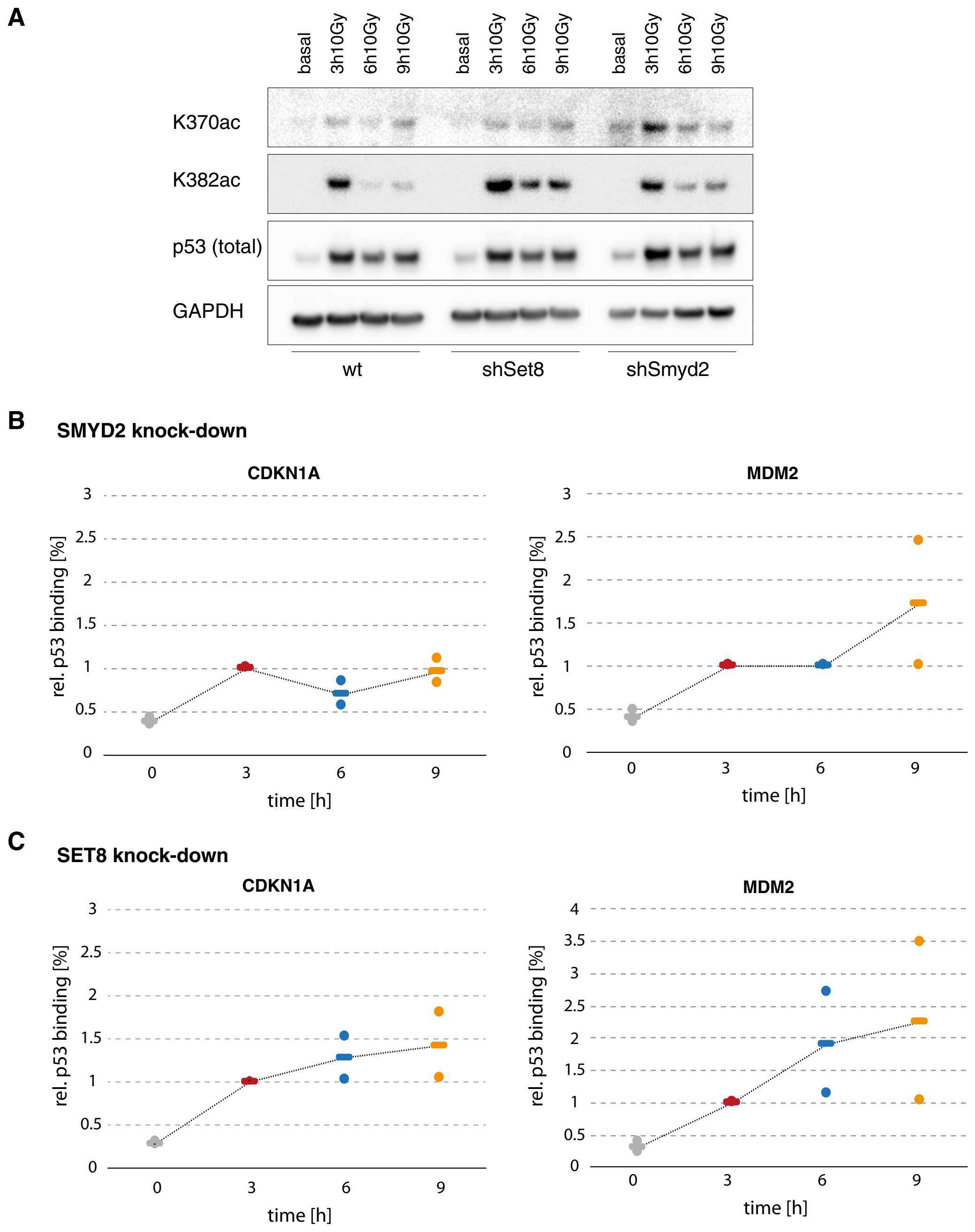


**Figure EV5. Smyd2 and Set8 activities affect p53 nuclear dynamics and promoter binding.**

A Western blot of acetylated p53 (K370/K382) in A549 Smyd2 and Set8 knockdown cells compared to wild-type cell lines shows an increase in acetylation specifically at later time points in the DNA damage response. Dynamics of total p53 remained pulse like. GAPDH is shown as loading control.

B, C Amount of p53 bound to CDKN1A and MDM2 promoters in A549 Smyd2 (B) and Set8 (C) knockdown cells before (basal, gray) and 3 h (red), 6 h (blue), and 9 h (orange) after DNA damage (10 Gy IR) as measured by ChIP. The amount of bound p53 was calculated as percentage of input and normalized to the time point of the first p53 peak at 3 h. Individual data points (mean values of triplicate quantification in qRT–PCR measurements) from two biological repeats are shown as dots; mean values are displayed as black horizontal lines. Dashed lines serve as guide to the eyes. We observed an increase in promoter binding at later time points similar to the results after Nutlin-3 treatment.

© 2019 The Authors
