# Supplementary material for: Stochastic transcription in the p53‐mediated response to DNA damage is modulated by burst frequency
Source: Mol Syst Biol. 2019 Dec 2;15(12):e9068. doi: 10.15252/msb.20199068 (PMC6886302; doi:10.15252/msb.20199068)
Supplement: Supplementary file 6 — Review Process File [file MSB-15-e9068-s011.pdf]

Stochastic transcription in the p53-mediated response to DNA damage is modulated by burst frequency.

Dhana Friedrich, Laura Friedel, Ana Finzel, Andreas Herrmann, Stephan Preibisch, Alexander Loewer.

Review timeline:

Submission date:	20th June 2019
Editorial Decision:	6th August.2019
Revision received:	16th October 2019
Editorial Decision:	21st October 2019
Revision received:	4th November 2019
Accepted:	7th November 2019

Editor: Maria Polychronidou

Transaction Report:

(Note: With the exception of the correction of typographical or spelling errors that could be a source of ambiguity, letters and reports are not edited. The original formatting of letters and referee reports may not be reflected in this compilation.)

1st Editorial Decision 6th August.2019

Thank you again for submitting your work to Molecular Systems Biology. We have now heard back from the three referees who agreed to evaluate your study. As you will see below, the reviewers acknowledge that the study addresses a relevant topic and presents potentially interesting findings. They raise however a series of concerns, which we would ask you to address in a major revision.

I think that the reviewers' recommendations are rather clear and there is therefore no need to repeat the comments listed below. Most of the reviewers' concerns refer to the need to provide further clarifications (both related to the model and the experiments) and to improve the presentation of the study in order to make the data and the main conclusions easily accessible to the reader.

REFEREE REPORTS

Reviewer #1:

The authors study the detailed kinetics of p53 dependent transcrptional reponses of mammalian genes after ionizing radation. The method used to resolve bursting parameters is to analyze smRNA FISH count distributions and compare with prediction from mathematical models. The burting patterns fall into three classes that cannot be distinguished by p53 levels or promoter occupancy. However, it is found that acetylation levels of a p53 lysine residue can explain gene specific responses.

The analysis of p53 bursting dynamics is certainly timely and relevant, and to my knowledge new. The experiments are carefully conducted, for example the perturbations showing response with

© European Molecular Biology Organization

reduced burst frequency for transient activation is convincing. The lack of correspondance between TF levels/localization and transcription responses is an emerging theme (see e.g. Tidin PMID:30926874) but has been shown only in few cases and it is thus relevant to document this further, here with p53. The proposed post-translation mechanism offers a mechanistic explanation. The authors should clarify the points raised below about terminology and verify some of the statements on the models.

Specific points:

Fig. 1C-D. It would be nice to show how the absolute levels compare in the RNA-seq and FISH data across all genes measured. Are the high basal levels observed in FISH consistent with RNA-seq?

It is quite interesting that the Fano factors are so large in the cytoplasm compared the nucleus (EV1B). Can the authors explain this? It would also be useful to show the corresponding plot with the CVs.

The authors need to explain better the model they use to interpret the data (perhaps mathematically), in particular which approximations to the telegrah model they consider, if any (e.g. short bursts, etc). For example in EV1D what are the plotted hyperbola? Similary in Fig. 2C, why does the burst size have units of inverse time ? As the name indicates, it should be a number, not a rate. I have the impression that the mu in the authors's notation is the transcription rate and not the burst size (as also suggested in their Figure 2A). This may just be a question of nomenclature but needs to be clarified. Of course the burst size is equal to the transcription rate times the on-time. It is usually quite convenient to use the burst size because otherwise one needs to estimate also the on time, which is more difficult from FISH. Has the on-time been estimated?

It needs to be explained in the methods how mu is calculated frm the data, citing Raj and Bahar Halpern is not sufficient.

When discussing variability (Fig. 1F, EV1C), it more useful to discuss the CV.

Note that the fraction of active promoters is not the same as the burst frequency. The two are proportional, and related through the elongation time and transcript life time.

"According to the random telegraph model, ... via higher burst size lead to increased noise (CV2)". The authors should check this because the CV only depends very weakly on the burst size (for b>1) and mostly depends on the inverse frequency. Moreover increased burst size when frequency is fixed leads to slightly decreased, not increased noise (Zoller PMID: 26215071).

p.5 The discussion of EV1D and gene specific correlation is unclear.

On p.6 the analysis of the first vs second pulse is very interesting. Does p53 repsonses behave similarly than other stimuli? Discuss this in comparison with Molina PMID:24297917.

Fig. 4D. Have the authors checked that Smyd2 KD leads to increased p53 acetylation? Or is this already known?

Minor:
The authors should talk about the modulation of burst frequency in the abstract since this is a main results as annouced in the title.

Reviewer #2:

In this manuscript Friedrich et al. examined mechanisms modulating the stochastic transcription of the p53-dependent target genes. More specifically, they used a pulsatile p53 expression system to address questions regarding how pulsatile accumulation of p53 results in establishing distinct gene expression programs, which they correlated with the binding affinity of p53 at its target genes. Furthermore, they perturbed with chemical compounds the pulsatile p53 expression pattern towards both a transient and a constitutive state of p53 expression and examined the behavior of its targets

genes. Using a variety of molecular methods, including smFISH, DNA FISH, Immunofluorescence, RNA-seq, qRT-PCR, ChIP, the authors provide a quantitative model of promoter activity at the single cell level, which shows that p53 dependent transcription upon IR is intrinsically stochastic and regulated mainly by burst frequency.
Overall, this study meets the journal's requirements regarding the novelty and the validity of the findings, highlighting that pulsatile p53 expression is absolutely required for the proper activation of its target genes. Nevertheless, there are concerns regarding the clarity of the text, especially in the discussion. For example, the general lack of a summarizing statement at the end of each paragraph in the Results section makes it difficult for the reader to follow the flow of the manuscript. Furthermore, although the authors present a consistent quantitative model, the Discussion section is full of speculations regarding epigenetic and post-transcriptional mechanisms which in general may or may not affect the behavior of the system. The manuscript needs extensive rewriting for better clarity and correction of the numerous spelling errors

1. Page 2 , line 16: The authors suggest directly that pulsatile expression of p53 is highly related with transient cell states like cell cycle arrest. They induce cell cycle arrest with ionizing radiation which causes DNA damage. How is this situation transient for a cell after such a stressful stimulus which causes irreversible changes to the genome integrity?

2. Figure 1C. The authors should provide data about the level of the p53 knock down.

3. Page 5 line 30: They should elaborate more on the strategy regarding the smFISH design they used. This is a highlight of the paper and it would be beneficial to show with a figure how they designed the probes to examine active sites of transcription.

4. Appendix figure S8A is not informative.
5. Figure 1D: There are only smFISH images regarding the basal state and not after the treatment with ionizing radiation. It would be more consistent to present all the images. Are the differences still detectable after the treatment?

6. Figure 1F. Dots representing the relative % input of total p53 binding in 0h should not be on the y axis' line as it is difficult to see both its value and standard deviation.

7. "As variability in mRNA levels could have other sources than bursty transcription such as differences in stability, we aimed to measure transcription states unambiguously in single cells after IR."
In reality, in this paragraph the authors just validate the findings of the first Results paragraph instead of searching for other mechanisms of mRNA levels regulation (confusing statement).
8. The authors claim that the major mechanism of the elevated mRNA levels upon IR induction is frequency burst increase. Will this be true for cells with only two genomic loci available? (diploid cells)

9. "...we validated the maximal number of genomic loci for a representative gene independently in A549 cells by DNA-FISH."
The validation should be done for more, if not all genes.

10. "To help our understanding of the observed gene-specific time-dependent patterns of stochastic gene expression, we defined the three promoter archetypes "sustained", "pulsatile" and "transient" and assigned our set of target genes gradually along this spectrum (Fig 2D)."
It is not stated in a clear way which archetype is assigned to each promoter neither in the text nor in the corresponding image. It is only mentioned later in text for only a subset of the genes.

11. "H3K27ac remained at high levels at the measured time points without notable differences (Appendix Fig S10)."
Acetylation levels of CDKN1a in fact rise substantially after induction, in contrast to the rest of the genes, but this is not mentioned/commented. It is not clear why the authors chose to show only these 3 genes. Are they representatives of each promoter archetype?

12. Why is the ChIP against p53 on PPM1D promoter missing (Fig 2F)? In cases of genes like BAX that have two p53 Response Elements, which one was chosen? Did the authors checked them both?

13. It would be interesting to discuss the possible role of the p53 Response Elements' sequence/number on each target gene's promoter in p53 binding (affinity) and gene expression profile. The only reference to the Res is the Fig EV1A.

14. "Despite high nuclear RNA counts for some targets such as BAX, MDM2 and CDKN1A, the nuclear to cytoplasmic ratio of mRNAs did not change for most of the analyzed targets upon IR (Appendix Fig S7), indicating that nuclear export is not limiting at this time scale."
"Furthermore, we did not observe noticeable changes in RNA stability upon IR for the selected genes and time-points upon IR."
Upon IR stimulation the percentage of nuclear RNAs of DDB2 is 2 fold increased compared to the basal levels. In addition, in Figure 2E, DDB2 shows a dramatic increase in the RNA degradation rate. The authors should explain if the high burst frequency in DDB2 gene upon IR is due to the augmented RNA degradation.

15. Figure 4D. K370ac western blot should be replaced by a clearer blot. In the blot which is provided it is difficult to detect the K370 levels and there are two protein bands in each well. Furthermore, all the blots should have the same levels of saturation. Moreover, the authors should provide in the raw data the blots of all 3 independent western blot experiments as the standard errors in the Nutlin-3 cells are too high. In addition, it is not clear which software is used for the quantification of Western Blots in Figure 4C. Based on the Figure 4B, the absolute K370ac levels increase upon Nutlin-3 treatment. However, the relative K370ac protein levels (K370ac/p53) seem to be reduced upon Nutlin-3 treatment.

16. Figure 4E. Since in Figures 4B and C it is clear that K383ac plays a more important role in p53 acetylation post IR, Figures 4D, E and F (p53-K370 methylase Smyd2 knockdown) should be replaced by the Figure EV4 (p53-K382 methylase Set8 knockdown).

17. "Even though the increase in burst frequency at 9 h after sequential treatment with Nutlin-3 was even stronger and may include also an impact of the change in integrated p53 abundance on bursting, this suggests that Smyd2 mediated methylation contributes to reduced transcription during the second p53 pulse for transient p53 targets."
It would be really interesting to perform a ChIP experiment in order to check the binding of p53 in wild type and shSmyd2 cells in the same locus with those used in Figure EV3E, before and after IR . In shSmyd2 cells, the binding profile of p53 should be similar with the Nutlin-3 treated cells (showing in Figure EV3E) that would be an addition evidence of p53 dynamics change from a pulsing to a sustained regime.

18. In the discussion section there is no reference to Figure 5 which is the concluded model of the study

Reviewer #3:

This paper by Loewer and colleagues does a nice job of addressing an important, unanswered question: what transcriptional parameters are regulated by complex, dynamic signaling pathway activity. They address this question in the context of the first two pulses of p53 activity after DNA damage, looking at 6 target genes with different overall expression levels and response dynamics. The authors conclude that p53 activation by DNA damage regulates the burst frequency but not burst size, except in the case of certain perturbations that also seem to alter the balance of p53 acetylation and/or methylation.

In general I am enthusiastic for publication of this thorough and interesting study. But in its current form, the authors have made many choices that make the manuscript *extremely* hard to follow for someone who is not deeply embedded in the RNA-FISH bursting model community. There are mislabeled panels, hidden raw data, missing figure legends, and analyses that are not clearly explained throughout the paper. I would strongly advocate for a clearer presentation, with some more raw data in the main figures and clearer explanations in the text/legends, so that the overall flow of the paper can be followed without having to place so much faith in the authors and "data not

shown". Virtually all of these changes could be made without requiring additional experiments, with the exception of two minor experiments related to the acetylation/methylation work in Figure 4.

My concerns and suggestions:

The "CDFs":

- Every probability distribution across Figures 1-4 is mislabeled as a "cumulative distribution function." This is incorrect! A CDF monotonically increases from 0 to 1. I assume the authors meant to refer to these as "probability density functions" (PDFs), and I assume they integrate to a value of 1. (I suppose it is possible that they are something else entirely.)

- For every figure, the "CDFs" (or PDFs, as is most likely) are fitted or smoothened using some kind of model that hides the raw data histograms. This fitting procedure is not described anywhere, and I am quite suspicious that it may distort the raw histograms substantially (this would be especially true if a single bin has a large number of cells in it, as would be expected for the '0' bin if there are many transcriptionally silent cells). Also, the raw data is hidden from the reader everywhere except in Figure 1D, so it is impossible even to gauge whether the fits are good or poor! It is also not clear whether the raw sample data was used to measure sample means, standard deviations, etc - or whether the fitted curves were used instead. Which choice was made, and can the authors justify it statistically?

Poorly labeled figures and missing panels:

- In Figure 1C, what are the different conditions treated with, and what are the fold-changes relative to? I assume for 'p53 wildtype', the comparison is between 0 h and 4 h after IR. For 'p53 knockdown', I am guessing the cells were not irradiated, but instead this is a comparison between unstimulated p53 knockdown and p53 wild-type cells. Is that correct? (The graduate student who helped me review the paper had the opposite guess as me, so I guess it is not clear to everyone.) Clearer figure legends that describe what was done, rather than how the authors would like the reader to interpret the data, would be helpful throughout the paper.

- The legend of Figure 1E-F is really hard to find, way off in the upper right-hand corner, even above the figure label. This label is also meant to apply to both panels 1E and 1F. For 1E, it would be helpful to at least label the x axis as 'time', or to place that legend close to the data panels in both 1E and 1F.

- Similarly, I am guessing that in Figure 1, the gene names labeled in 1D are supposed to carry through all the way down through 1E and 1F, so that MDM2 labels everything on the left, etc etc. I would strongly advocate for labeling individual plots - it is not obvious that we should be using gene names from other panels. (Perhaps in 1E, the authors can label the y axis "MDM2 RNAs/cell"...)

- In Figure 2D's "active TSS/cell [%]" figure, the data for SESN1 is just a copy of the data for DDB2. Presumably there is real SESN1 data somewhere to add here!

- Do Figures 2E-F really show means + standard deviations from 3 independent experiments, as listed in the legend!? Many standard deviations are so tiny that they cannot even be seen over the size of the data points. It is hard for me to conceive that any biological replicates in any assay could be this precisely overlapping in all cases. Also, why not just plot the individual data points for all three experiments instead of their mean + SD?

Understanding burst fraction and burst size in Figure 2

I had a big problem with the assumptions made in Figure 1 - where changes to the Fano factor are interpreted specifically as changes to burst frequency. This is a terribly indirect method to infer something about a highly specific process, especially since this work pushes far outside the telegraph model's usual assumptions. (The usual bursting kinetics models were developed for stationary processes, where the probability distributions are not changing and system dynamics have reached some kind of steady state. This is clearly not the case after acute stimulation, while the upstream signaling pathway is also varying over time!)

Fortunately, my impression is that the authors had the same suspicions and used an elegant, direct approach to measure burst size and frequency in Figure 2. Let me describe my understanding here in case I am missing something: by double-labeling intronic and exonic RNAs, the authors can identify nascent transcription sites (from the intron signal) and the total number of tethered nascent RNAs at that site (from the exon signal). Then the total exon brightness of a spot is a measurement of the burst size, and the number of intronic foci per cell is the fraction of TSSs that are bursting at the current time point, and thus directly related to burst frequency (assuming that bursts turn on & off reasonably fast). With this direct of a measurement, no mathematical modeling is necessary at all, correct? It would be great for the authors to provide a more clear explanation of these points, including how they do not rely on a model with easily-violated assumptions! It would also be really great to have some images of raw data in the main text, showing an image of intron and exon foci and describing how they will be quantified in Figure 2D.

Now, with all that said, I am confused by a number of things in how the resulting data is presented. In Figure 2D, what is "active TSS / cell [%]", and how can it be measured for different sub-populations of cells (those with "strong" vs "weak" TSS activity)? I can imagine that some cells have 1 TSS, some 2, some 3, so that the y axis could be a stacked bar of the % of cells with these different numbers of TSSs. But that is not what the figure or legend describe! Alternatively, the authors could do what they say in Figure 2C and just count the total # of TSSs and the total # of genomic loci, and divide them to get 'f'. That appears not to be what was done either! Whatever the case, the authors must state (and clearly justify) a mathematical equation that describes what they are plotting on the y-axis, and how it relates to the burst frequency, which I think is what we really care about.

For the smoothened histogram plots in Figure 2D, the plots are labeled as "burst size / TSS" above and "μ/TSS" below. What do these quantities actually mean? Aren't they just histograms of μ, the quantification of the brightness of exon spots that colocalize with intron spots, right? Something like integrated intensity of the exon probe at each locus? I do not understand the description in the figure legend: "TSS activity divided by the number of active TSS shown as cumulative distribution functions". What is "TSS activity" defined to be? The integrated intensity of a single exon focus? If this is quantified for each TSS individually, why would one need to divide by the # of active TSS per cell? Or are the authors actually plotting μ/n, where n is the total number of loci?

My apologies for this long-winded section. I made a serious effort to understand this paper and I don't think I'm completely obtuse, so I conclude that other readers will probably share my confusion and would appreciate clarification on these points.

Other miscellaneous confusion

- Which genes do the authors assign to each 'archetype', and what does this teach us? I see the schematic of the archetypes in Figure 2, but no effort is made to classify actual genes based on these archetypes in the text or the figure. I guess I can do it by eye myself: Mdm2 and p21 are transient, PPM1D and BAX are pulsatile, and DDB2 is sustained? (I don't know what SESN1 looks like yet...) It appears that the same conclusions could be drawn from the total RNA levels in Fig 1E - is that correct? This observation seems to further confirm that RNA degradation rates are not regulated, so that promoter activity and total RNA dynamics are well-correlated to one another. Maybe that is worth discussing briefly?

Two minor but important experiments for the acetylation/methylation experiments

- The authors knock down Smyd2 and see a change in p21 and Mdm2 transcription. But two crucial experiments need to be done in order to interpret this result cleanly:
First, what do p53 levels look like over time in Smyd2 KD cells - is it still pulsatile? We see that p21 and Mdm2 dynamics are no longer transient, and it is important to know whether this is because p53 itself is no longer transient (as in the case of Nutlin).
Second, is p53 methylation or acetylation actually affected by this knockdown? The authors should perform a Western or some kind of PTM analysis to test whether the Smyd2 KD has any effect on p53 itself in their hands.

© European Molecular Biology Organization

Response to Reviewers

Reviewer #1:

Point 1: *It would be nice to show how the absolute levels compare in the RNA-seq and FISH data across all genes measured. Are the high basal levels observed in FISH consistent with RNA-seq? (Related to Fig. 1C-D.)*

For comparison, we added RPKM values for our RNAseq data (MCF10A) as well as for published data (MCF7, Hafner 2019) and present it with our quantified RNA counts from smFISH datasets in A549 in an overview table (new Appendix Fig. S1D). In brief, high basal levels of p53 target genes can be observed in RNAseq data as well, although there are cell line specific differences. In addition, we replaced Figure 1C with qPCR data from A549 to consistently present data from only one cell line in the main figures (revised Fig. 1C). We now also include qPCR data for the level of p53 knock-down in A549 and MCF10A cell in new Appendix Fig S1A-B (see Reviewer #2 Point 2)..

Point 2: *It is quite interesting that the Fano factors are so large in the cytoplasm compared the nucleus (EV1B). Can the authors explain this? It would also be useful to show the corresponding plot with the CVs.*

We now show total nuclear and cytoplasmic noise $\eta^2 = \frac{\sigma^2}{\mu^2} = CV^2$ in Figure EV1B and C. Additionally, we present the corresponding plots of the noise strength measured as $\frac{\sigma^2}{\mu}$ (Fano factor) in the appendix (revised Fig. EV1C-D and Appendix Fig. S5). We initially focused on the Fano factor because of recent work by Hansen et al. that suggests to use Fano factor for comparing nuclear and cytoplasmic variability as it is independent of Poisson scaling and measures noise as the deviation from a Poisson process (Hansen et al., 2018). When CV^2 is used, the differences in mean RNA levels between nucleus and cytoplasm need to be taken into account. Without further experimental studies, we can only speculate that high cytoplasmic Fano factors are caused by additional molecular processes that act as noise sources such as multi-step mRNA translation and degradation (Hansen et al. Cell Systems 2018, Baudrimont et al. Cell Reports 2019). We now mention this hypothesis in the main text.

Point 3.1: *The authors need to explain better the model they use to interpret the data (perhaps mathematically), in particular which approximations to the telegraph model they consider, if any (e.g. short bursts, etc). For example in EV1D what are the plotted hyperbola?*

In line with previous studies, we assume low burst frequencies (k_{off} >> k_{on}) when applying the telegraph model. The hyperbola in Fig. EV1D correspond to the equation $CV^2 = \frac{\frac{T}{k_off}}{<mRNA>}$ where T is the transcription rate and k_{off} the rate of the promoter switching to the off state (Dar et al. 2016). Accordingly, $\frac{T}{k_off}$ corresponds to the burst size. Hyberbola corresponding to burst sizes ranging from 5 to 70 are presented (as indicated on top of the graph). We expanded the corresponding information in the main text and the figure legend.

Point 3.2: *Similary in Fig. 2C, why does the burst size have units of inverse time ? As the name indicates, it should be a number, not a rate. I have the impression that the mu in the authors's notation is the transcription rate and not the burst size (as also suggested in their Figure 2A). This may just be a question of nomenclature but needs to be clarified. Of course the burst size is equal to the transcription rate times the on-time. It is usually quite convenient to use the burst size because otherwise one needs to estimate also the on time, which is more difficult from FISH. Has the on-time been estimated?*

The reviewer is correct, μ indeed corresponds to the transcription rate. Using smFISH, we only estimated the fraction of active promoters, not the on-time. We apologize for the unclear nomenclature and have reworked the manuscript to be more precise and consistent in terminology. We now use *transcription rate* and *fraction of active promoters* throughout the manuscript and relate these to burst size and frequency.

Point 4: *It needs to be explained in the methods how mu is calculated from the data, citing Raj and Bahar Halpern is not sufficient.*

We now included a detailed description of our calculation in the Methods section and added further information on the calculation the correction factor for probe position (new Appendix Fig. S10). In brief, we use the fraction of active promoters as a proxy for burst frequency. The transcription rate μ at active TSS is calculated from the estimated number of nascent RNAs (from fluorescence intensity at the TSS), probe positioning, gene length and a constant elongation speed. For a better impression of TSS activity, RNAP2 occupancies at active TSS are plotted along with fluorescence intensities at TSS and RNAP2 occupancies per cell in Figure EV2C.

Point 5: *When discussing variability (Fig. 1F, EV1C), it more useful to discuss the CV.*

We have added corresponding plots using CV^2 for Fig. EV1C (see also Point 2) and provide CV values in Fig. 1F. We adjusted the corresponding sections in the main text as well.

Point 6: *Note that the fraction of active promoters is not the same as the burst frequency. The two are proportional, and related through the elongation time and transcript life time.*

We would again like to apologize for unclear nomenclature. We have reworked the figures and main text to provide more precise and consistent terminology (see Point 3.2)

Point 7: *"According to the random telegraph model, ... via higher burst size lead to increased noise (CV2)". The authors should check this because the CV only depends very weakly on the burst size (for b>1) and mostly depends on the inverse frequency. Moreover increased burst size when frequency is fixed leads to slightly decreased, not increased noise (Zoller PMID: 26215071).*

We have clarified the corresponding section and now focus on the expected noise scaling if mean RNA expression is increased by more frequent promoter activation alone (increased burst frequency) according to previous studies (Dar 2012, Dar 2016).

Point 8: *p.5 The discussion of EV1D and gene specific correlation is unclear.*

We reworked the corresponding section (see above) and now mention explicit examples of deviations from the expected noise scaling

Point 9: *On p.6 the analysis of the first vs second pulse is very interesting. Does p53 responses behave similarly than other stimuli? Discuss this in comparison with Molina PMID:24297917.*

We thank the reviewer for pointing out this relevant publication. Similarities and differences to the p53 system are now addressed in the discussion section.

Point 10: *Fig. 4D. Have the authors checked that Smyd2 KD leads to increased p53 acetylation? Or is this already known?*

We used western blot analysis to validate that Smyd2 and SET8 knock-down leads to increased p53 acetylation at residues 370 and 382, respectively, and added the corresponding data to new Fig. EV5A.

Point 11: *The authors should talk about the modulation of burst frequency in the abstract since this is a main results as annouced in the title.*

In the abstract, we mention modulation of burst frequency in the following context: “We found that p53 target promoters are regulated by frequency modulation of stochastic bursting and can be grouped along three archetypes of gene expression.”

Reviewer #2:

Point 1: *Page 2 , line 16: The authors suggest directly that pulsatile expression of p53 is highly related with transient cell states like cell cycle arrest. They induce cell cycle arrest with ionizing radiation which causes DNA damage. How is this situation transient for a cell after such a stressful stimulus which causes irreversible changes to the genome integrity?*

The cell fate upon ionizing radiation depends on the dose, which correlates with the duration of p53 pulsing dynamics. In the cited publication (Purvis 2012), it was shown that at 2.5 and 5 Gy, the majority of cells are able to repair the DNA damage and resume cycling when p53 accumulation is pulsatile. When p53 dynamics are converted to sustained accumulations using a small molecule, the cells preferentially induce senescence. We now mention dose-dependency in the corresponding section to clarify this point.

Point 2: *Figure 1C. The authors should provide data about the level of the p53 knock down.*

We now provide measurements of p53 knockdown levels by qPCR for A549 and MCF10A cells (new Appendix Fig. S1A-B, see also Reviewer #1, Point 1).

Point 3: *Page 5 line 30: They should elaborate more on the strategy regarding the smFISH design they used. This is a highlight of the paper and it would be beneficial to show with a figure how they designed the probes to examine active sites of transcription.*

We added a figure panel highlighting the general design of the smFISH probes (new Fig. EV1B) as well as additional information regarding the identification of transcriptional start sites (new Fig EV2A-C). Moreover, we provide additional details regarding quantification and modeling of smFISH data (see Reviewer #1, Point 3).

Point 4: *Appendix figure S8A is not informative.*

We reworked Appendix Fig. S8A of our initial submission and integrated it into the DNA FISH analysis of additional target genes (now Appendix Fig. S9, see below). We show that for most tested p53 target genes the maximum number of TSS that we identified by smFISH based co-localization fits the DNA measurements for genomic loci. For PPM1D we were not successful to generate DNA FISH probes from genomic DNA. Even commercial RP11 based BAC probes for DNA FISH, as used for the other targets, were not obtainable for PPM1D. As all performed DNA FISH experiments confirmed our smFISH based TSS identification, it is likely that also PPM1D measurements are correct.

Point 5: *Figure 1D: There are only smFISH images regarding the basal state and not after the treatment with ionizing radiation. It would be more consistent to present all the images. Are the differences still detectable after the treatment?*

To provide examples of smFISH images after irradiation, we now show a set of images acquired at 3h after 10Gy y-IR (new Fig. 2A). The variability of expression is still existent after DNA damage as visible in the quantification of RNAs / cell (Fig. 2B-C). As the nature of the images is repetitive and as images of individual cells do not themselves provide a lot of additional information, we decided against showing smFISH images for time points beyond 3h.

Point 6: *Figure 1F. Dots representing the relative % input of total p53 binding in 0h should not be on the y axis' line as it is difficult to see both its value and standard deviation.*

As suggested, we adjusted the data visualization to prevent data points on the lines representing y-axes (updated Fig. 3E and G, , Fig. EV5 B-C, EV4E and Appendix Fig. S11).

Point 7: *"As variability in mRNA levels could have other sources than bursty transcription such as differences in stability, we aimed to measure transcription states unambiguously in single cells after IR." In reality, in this paragraph the authors just validate the findings of the first Results paragraph instead of searching for other mechanisms of mRNA levels regulation (confusing statement).*

We thank the reviewer for pointing this out and adjusted our wording in the corresponding paragraph to avoid confusion.

Point 8: *The authors claim that the major mechanism of the elevated mRNA levels upon IR induction is frequency burst increase. Will this be true for cells with only two genomic loci available? (diploid cells)*

To address this question, we analyzed stochastic expression of Mdm2 in non-transformed diploid MCF10A cells in basal state and 3h after 10Gy IR (new Fig. EV3). In brief, we also observed an increased fraction of active promoters, while the transcription rate remained relatively constant, which is consistent with the data presented for A549 cells.

Point 9: *"...we validated the maximal number of genomic loci for a representative gene independently in A549 cells by DNA-FISH." The validation should be done for more, if not all genes.*

We now provide additional DNA FISH data in A549 cells for Mdm2, Bax, DDB2 and SESN1 in addition to the previous measurement for CDKN1A in the revised Appendix Fig. 9. The analysis confirms our previous assumptions based on smFISH data (see also Point 4).

Point 10. *"To help our understanding of the observed gene-specific time-dependent patterns of stochastic gene expression, we defined the three promoter archetypes "sustained", "pulsatile" and "transient" and assigned our set of target genes gradually along this spectrum (Fig 2D)." It is not stated in a clear way which archetype is assigned to each promoter neither in the text nor in the corresponding image. It is only mentioned later in text for only a subset of the genes.*

As the promoter archetypes represent prototypical expression patterns, we are hesitant to unambiguously assign them to specific target genes. Most of the target genes do not perfectly fit to the sustained, pulsatile and transient archetypes, but instead, we see them gradually aligning along the axis defined by the prototypical expression patterns. We now provide a corresponding visualization in the main figure and refer to it in the main text (new Fig. 3F).

Point 11: *"H3K27ac remained at high levels at the measured time points without notable differences (Appendix Fig S10)." Acetylation levels of CDKN1a in fact rise substantially after induction, in contrast to the rest of the genes, but this is not mentioned/commented. It is not clear why the authors chose to show only these 3 genes. Are they representatives of each promoter archetype?*

The analyzed genes represent the transient and sustained promoter archetypes (see below for issues with PPM1D as a representative of the pulsatile archetype). There is indeed an increase in H3K27ac in the the CDKN1A promoter. However, as H3K27 acetylation in the MDM2 promoter, which also shows transient expression, is comparable to the BAX promoter showing sustained expression, it is unlikely that H3K27 acetylation is causatively related to expression archetypes.

We now expanded our discussion of the results in the corresponding section of the main text to emphasize this line of thought.

Point 12: *Why is the ChIP against p53 on PPM1D promoter missing (Fig 2F)? In cases of genes like BAX that have two p53 Response Elements, which one was chosen? Did the authors checked them both*?

We attempted to measure p53 binding to the published binding site in the PPM1D promoter using ChIP but didn't obtained sufficient signals. While several p53 response elements have been predicted (Talibalidi 2015), this is consistent with a lack of ChIP data for PPM1D in the literature (e.g. Fischer, 2017). We now explicitly mention this in the legend of the corresponding figure. Moreover, we indicate the p53 Response Elements analyzed by ChIP in the overview figure of the target gene structure (updated Fig. EV1A).

Point 13: *It would be interesting to discuss the possible role of the p53 Response Elements' sequence/number on each target gene's promoter in p53 binding (affinity) and gene expression profile. The only reference to the Res is the Fig EV1A.*

So far, we could not detect any correlation between number and location of p53 binding sites or their predicted (measured) affinities with expression profiles. However, we have so far only analyzed a small subset of the characterized p53 target genes and a more systematic analysis may provide further insights. We now mention this in the first paragraph of our discussion section.

Point 14. *"Despite high nuclear RNA counts for some targets such as BAX, MDM2 and CDKN1A, the nuclear to cytoplasmic ratio of mRNAs did not change for most of the analyzed targets upon IR (Appendix Fig S7), indicating that nuclear export is not limiting at this time scale." "Furthermore, we did not observe noticeable changes in RNA stability upon IR for the selected genes and time-points upon IR." Upon IR stimulation the percentage of nuclear RNAs of DDB2 is 2 fold increased compared to the basal levels. In addition, in Figure 2E, DDB2 shows a dramatic increase in the RNA degradation rate. The authors should explain if the high burst frequency in DDB2 gene upon IR is due to the augmented RNA degradation.*

We apologize for neglecting this regulation of the DDB2 mRNA. We now explicitly mention the altered ratio of nuclear to cytoplasmic DDB2 RNA and the increased degradation rates upon irradiation in the corresponding sections of the main text. Both are consistent with reported post-transcriptional regulation via the 3'-UTR of DDB2 (Melanson 2013). As we determine the fraction of

active promoters directly from staining of nascent RNAs, augmented RNA degradation should have no affect on this measurement.

Point 15: *Figure 4D. K370ac western blot should be replaced by a clearer blot. In the blot which is provided it is difficult to detect the K370 levels and there are two protein bands in each well. Furthermore, all the blots should have the same levels of saturation. Moreover, the authors should provide in the raw data the blots of all 3 independent western blot experiments as the standard errors in the Nutlin-3 cells are too high. In addition, it is not clear which software is used for the quantification of Western Blots in Figure 4C. Based on the Figure 4B, the absolute K370ac levels increase upon Nutlin-3 treatment. However, the relative K370ac protein levels (K370ac/p53) seem to be reduced upon Nutlin-3 treatment.*

It is not trivial to detect acetylation of p53 at K370 using the available antibodies. Despite our own dissatisfaction with the relative low quality of the blots, we believe that our data is comparable with other reports in the field. Nevertheless, we have improved the presentation to show blots with similar levels of saturation and choose a different area to avoid the unspecific band that may cause confusion (updated Fig. 5B). We now also show all independent western blot experiments in the Appendix using wider clipping to reveal the unspecific band in p53-K370ac (new Appendix Fig. S12) and describe the quantification procedure in the methods section.

Point 16. *Figure 4E. Since in Figures 4B and C it is clear that K382ac plays a more important role in p53 acetylation post IR, Figures 4D, E and F (p53-K370 methylase Smyd2 knockdown) should be replaced by the Figure EV4 (p53-K382 methylase Set8 knockdown).*

We decided to show both the results for the Smyd2 and the Set8 knockdown in the main figure to provide easier access to the data (revised Fig 5D-I).

Point 17: *"Even though the increase in burst frequency at 9 h after sequential treatment with Nutlin-3 was even stronger and may include also an impact of the change in integrated p53 abundance on bursting, this suggests that Smyd2 mediated methylation contributes to reduced transcription during the second p53 pulse for transient p53 targets." It would be really interesting to perform a ChIP experiment in order to check the binding of p53 in wild type and shSmyd2 cells in the same locus with those used in Figure EV3E, before and after IR . In shSmyd2 cells, the binding profile of p53 should be similar with the Nutlin-3 treated cells (showing in Figure EV3E) that would be an addition evidence of p53 dynamics change from a pulsing to a sustained regime.*

We performed the suggest ChIP experiments in Smyd2 and Set8 knockdown cells (new Fig. EV5B-C). As predicted by the reviewer, we observed increasing p53 binding to the MDM2 and CDKN1A promoters upon DNA damage compared to wild-type cells similar to Nutlin-3 treated cells. This supports the transition from a transient to a sustained expression regime for these genes.

Point 18. *In the discussion section there is no reference to Figure 5 which is the concluded model of the study.*

We thank the reviewer for pointing this out to us. As we felt that the figure was redundant, we removed it in the revised version of the manuscript.

__Reviewer #3:__

Point 1.1: *Every probability distribution across Figures 1-4 is mislabeled as a "cumulative distribution function." This is incorrect! A CDF monotonically increases from 0 to 1. I assume the authors meant to refer to these as "probability density functions" (PDFs), and I assume they integrate to a value of 1. (I suppose it is possible that they are something else entirely.) For every figure, the "CDFs" (or PDFs, as is most likely) are fitted or smoothened using some kind of model that hides the raw data histograms. This fitting procedure is not described anywhere, and I am quite suspicious that it may distort the raw histograms substantially (this would be especially true if a single bin has a large number of cells in it, as would be expected for the '0' bin if there are many transcriptionally silent cells).*

We apologize for this mistake. As correctly identified by the reviewer, we present probability density estimates in the corresponding figure panels. The estimates are based on kernel density estimation using a normal kernel evaluated at equally spaced points. We choose this visualization method to allow comparison of multiple distributions in one plot. To make our choices more transparent, we now include a Data visualization section in the methods parts.

Point 1.2: *Also, the raw data is hidden from the reader everywhere except in Figure 1D, so it is impossible even to gauge whether the fits are good or poor! It is also not clear whether the raw sample data was used to measure sample means, standard deviations, etc - or whether the fitted curves were used instead. Which choice was made, and can the authors justify it statistically?*

We want to emphasize here that raw data was used for all calculation throughout the manuscript. Fits such as probability density estimates were exclusively used for data visualization purposes. We now explicitly state this in the methods section. To allow the reader to judge fit quality, we

additionally show histograms and the corresponding fits in the Appendix (new Appendix Fig. S4). Furthermore, to allow easy access to raw data, we make use of the Expanded View features of MSB and provide source data for main figure panels that allow interested readers to re-plot the data in any possible way. As in our initial submission, we also provide the most important measurements in form of Expanded View Dataset S1.

Point 2.1: *In Figure 1C, what are the different conditions treated with, and what are the fold-changes relative to? I assume for 'p53 wildtype', the comparison is between 0 h and 4 h after IR. For 'p53 knockdown', I am guessing the cells were not irradiated, but instead this is a comparison between unstimulated p53 knockdown and p53 wild-type cells. Is that correct? (The graduate student who helped me review the paper had the opposite guess as me, so I guess it is not clear to everyone.) Clearer figure legends that describe what was done, rather than how the authors would like the reader to interpret the data, would be helpful throughout the paper.*

In Figure 1C, the plot shows the relative fold change of target gene expression at 4h after 10Gy IR compared to the basal state (without DNA damage) both for p53 wild-type and for p53 knockdown cells. We clarified this both in the figure itself and in the figure legend. Please also see Reviewer #1 Point 1 for changes to this figure panel. Moreover, we reworked all figure legends to provide more detailed descriptions of what was done.

Point 2.2.: *The legend of Figure 1E-F is really hard to find, way off in the upper right-hand corner, even above the figure label. This label is also meant to apply to both panels 1E and 1F. For 1E, it would be helpful to at least label the x axis as 'time', or to place that legend close to the data panels in both 1E and 1F.*

To improve clarity, we now repeat legends for individual figure panels. We also improved axis labels for plots in Figure 1 and throughout the manuscript.

Point 2.3: Similarly, I am guessing that in Figure 1, the gene names labeled in 1D are supposed to carry through all the way down through 1E and 1F, so that MDM2 labels everything on the left, etc etc. I would strongly advocate for labeling individual plots - it is not obvious that we should be using gene names from other panels. (Perhaps in 1E, the authors can label the y axis "MDM2 RNAs/cell"...)

As suggested by the reviewer, we now label individual plots either by titles or by extended axis labels.

Point 2.4: *In Figure 2D's "active TSS/cell [%]" figure, the data for SESN1 is just a copy of the data for DDB2. Presumably there is real SESN1 data somewhere to add here!*

We apologize for the mistake and thank the reviewer for pointing this out. In fact, the SESN1 plot was correct and unintentionally duplicated instead of showing the corresponding data for DDB2. We replaced the figure panel with the correct plot for DDB2 (new Fig 3D) .

Point 2.5: *Do Figures 2E-F really show means + standard deviations from 3 independent experiments, as listed in the legend!? Many standard deviations are so tiny that they cannot even be seen over the size of the data points. It is hard for me to conceive that any biological replicates in any assay could be this precisely overlapping in all cases. Also, why not just plot the individual data points for all three experiments instead of their mean + SD?*

As suggested by the reviewer, we now show individual data points as well as the mean for all ChIP experiments (revised Figure 3G, EV4E and Appendix Figure S12).

Point 3.1: *I had a big problem with the assumptions made in Figure 1 - where changes to the Fano factor are interpreted specifically as changes to burst frequency. This is a terribly indirect method to infer something about a highly specific process.*

As this approach has been used in the past, we present it as segue to our more direct measurements of promoter features. We now state this more directly in the main text.

Point 3.2 *It would be great for the authors to provide a more clear explanation of [the approach to quantify stochastic gene expression directly from smFISH data], including how they do not rely on a model with easily-violated assumptions! It would also be really great to have some images of raw data in the main text, showing an image of intron and exon foci and describing how they will be quantified in Figure 2D.*

We now provide a more detailed explanation of our analysis of smFISH data and how we derived parameters such as fraction of active promoters and transcription rates both in Fig EV1,EV2 , Appendix Fig. S10 and in the method section (see also Reviewer #1 Point 4 and Reviewer #2 Point 3).

Point 3.3: *In Figure 2D, what is "active TSS / cell [%]", and how can it be measured for different sub-populations of cells (those with "strong" vs "weak" TSS activity)? I can imagine that some cells have 1 TSS, some 2, some 3, so that the y axis could be a stacked bar of the % of cells with these*

different numbers of TSSs. But that is not what the figure or legend describe! Alternatively, the authors could do what they say in Figure 2C and just count the total # of TSSs and the total # of genomic loci, and divide them to get 'f'. That appears not to be what was done either! Whatever the case, the authors must state (and clearly justify) a mathematical equation that describes what they are plotting on the y-axis, and how it relates to the burst frequency, which I think is what we really care about.

We apologize for the misleading labeling. What we indeed showed was the percentage of cells with active promoters. In our initial submission, we divided the populations into cells where almost all TSS are activated ("strong TSS activity") and those were only a subset was activity ("partial TSS activity") for better visualization in the stacked bar graph plot. The detailed criteria for the subdivision was provided in the methods section. We now simplified the scheme and consistently classify cells with 75% or more TSS active as having "strong TSS activity" and those with at least 1 but less than 75% of TSS active as having "partial TSS activity". We added this information both to the methods section and the figure legends.

In addition, we already provided the mean fraction of active TSS (total # of TSS divided # of genomic loci) as a number ('f') below the stacked bar graph plot. We now moved these numbers to the top of the graph, labeled them more clearly and added corresponding information to the legend.

Point 3.4: *For the smoothened histogram plots in Figure 2D, the plots are labeled as "burst size / TSS" above and "μ/TSS" below. What do these quantities actually mean? Aren't they just histograms of μ, the quantification of the brightness of exon spots that colocalize with intron spots, right? Something like integrated intensity of the exon probe at each locus? I do not understand the description in the figure legend: "TSS activity divided by the number of active TSS shown as cumulative distribution functions". What is "TSS activity" defined to be? The integrated intensity of a single exon focus? If this is quantified for each TSS individually, why would one need to divide by the # of active TSS per cell? Or are the authors actually plotting μ/n, where n is the total number of loci?*

The transcription rate μ shown in Figure 2D (new Fig 3D) is derived from the RNAP2 occupancy, which is the integrated fluorescence intensity of active TSS divided by the average fluorescence intensity of all non-TSS spot corrected for probe positioning. To calculate μ, RNAP2 occupancies are multiplied by the ratio of polymerase speed (v) and gene length (L). We calculate μ both for individual TSS (shown in Figure 2D/ new Fig 3D) as well as for entire cells (sum of RNAP2 occupancies from all active TSS multiplied by v/L). This rate is shown together with raw TSS intensities and RNAP2 occupancies (at TSS) in Figure EV2C. To make our calculations more transparent, we have added more details to the method section (see Reviewer #1 Point 4 as well).

Point 4: *Which genes do the authors assign to each 'archetype', and what does this teach us? I see the schematic of the archetypes in Figure 2, but no effort is made to classify actual genes based on these archetypes in the text or the figure. I guess I can do it by eye myself: Mdm2 and p21 are transient, PPM1D and BAX are pulsatile, and DDB2 is sustained? (I don't know what SESN1 looks like yet...) It appears that the same conclusions could be drawn from the total RNA levels in Fig 1E - is that correct? This observation seems to further confirm that RNA degradation rates are not regulated, so that promoter activity and total RNA dynamics are well-correlated to one another. Maybe that is worth discussing briefly?*

We now provide a visual attribution of target genes to archetypes in the main figures and refer to it in the main text (new Fig. 3F). Please see Reviewer #2 Point 10 for a more detailed discussion. In general, we agree with the Reviewer that patterns of promoter activity can be observed in total RNA levels as degradation rates are not regulated for most genes. However, as degradation rates are only inferred and not directly measured, we are hesitant to highlight it too strongly.

Point 5.1: *What do p53 levels look like over time in Smyd2 KD cells - is it still pulsatile? We see that p21 and Mdm2 dynamics are no longer transient, and it is important to know whether this is because p53 itself is no longer transient (as in the case of Nutlin).*

We measured total p53 in Smyd2 and Set8 knockdown cell lines using western blot analysis (new Fig. EV5A). We found that it is still largely pulsatile.

Point 5.2: *Second, is p53 methylation or acetylation actually affected by this knockdown? The authors should perform a Western or some kind of PTM analysis to test whether the Smyd2 KD has any effect on p53 itself in their hands.*

We measured p53 acetylation at K370 and K383 in Smyd2 and Set8 knockdown cell lines using western blot analysis (new Fig. EV5A) and found increased modification levels as expected (see Reviewer #1 Point 10).

2nd Editorial Decision 21st October 2019

Thank you again for submitting your work to Molecular Systems Biology. We are now satisfied with the modifications made and we think that the study is now suitable for publication.

Before we can formally accept your manuscript for publication, we would ask you to address some remaining editorial issues.

© European Molecular Biology Organization

EMBO PRESS

YOU MUST COMPLETE ALL CELLS WITH A PINK BACKGROUND

PLEASE NOTE THAT THIS CHECKLIST WILL BE PUBLISHED ALONGSIDE YOUR PAPER

Corresponding Author Name: Alexander Löwer

Journal Submitted to: Molecular Systems Biology
Manuscript Number: MSB-19-9068

USEFUL LINKS FOR COMPLETING THIS FORM

http://www.antibodypedia.com
http://1degreebio.org
http://www.equator-network.org/reporting-guidelines/improving-bioscience-research-reporting-the-arrive-
http://grants.nih.gov/grants/olaw/olaw.htm
http://www.mrc.ac.uk/Ourresearch/Ethicsresearchguidance/Useofanimals/index.htm
http://ClinicalTrials.gov
http://www.consort-statement.org
http://www.consort-statement.org/checklists/view/32-consort/66-title
http://www.equator-network.org/reporting-guidelines/reporting-recommendations-for-tumour-marker-pro
http://datadryad.org
http://figshare.com
http://www.ncbi.nlm.nih.gov/gap
http://www.ebi.ac.uk/ega
http://biomodels.net/
http://biomodels.net/miriam/
http://jjj.biochem.sun.ac.za
http://oba.od.nih.gov/biosecurity/biosecurity_documents.html
http://www.selectagents.gov/

Reporting Checklist For Life Sciences Articles (Rev. June 2017)

This checklist is used to ensure good reporting standards and to improve the reproducibility of published results. These guidelines are consistent with the Principles and Guidelines for Reporting Preclinical Research issued by the NIH in 2014. Please follow the journal's authorship guidelines in preparing your manuscript.

A- Figures
1. Data
The data shown in figures should satisfy the following conditions:

- the data were obtained and processed according to the field's best practice and are presented to reflect the results of the experiments in an accurate and unbiased manner.
- figure panels include only data points, measurements or observations that can be compared to each other in a scientifically meaningful way.
- graphs include clearly labeled error bars for independent experiments and sample sizes. Unless justified, error bars should not be shown for technical replicates.
- if n< 5, the individual data points from each experiment should be plotted and any statistical test employed should be justified
- Source Data should be included to report the data underlying graphs. Please follow the guidelines set out in the author ship guidelines on Data Presentation.

2. Captions

Each figure caption should contain the following information, for each panel where they are relevant:

- a specification of the experimental system investigated (eg cell line, species name).
- the assay(s) and method(s) used to carry out the reported observations and measurements
- an explicit mention of the biological and chemical entity(ies) that are being measured.
- an explicit mention of the biological and chemical entity(ies) that are altered/varied/perturbed in a controlled manner.
- the exact sample size (n) for each experimental group/condition, given as a number, not a range;
- a description of the sample collection allowing the reader to understand whether the samples represent technical or biological replicates (including how many animals, litters, cultures, etc.).
- a statement of how many times the experiment shown was independently replicated in the laboratory.
- definitions of statistical methods and measures:
  - common tests, such as t-test (please specify whether paired vs. unpaired), simple χ2 tests, Wilcoxon and Mann-Whitney tests, can be unambiguously identified by name only, but more complex techniques should be described in the methods section;
  - are tests one-sided or two-sided?
  - are there adjustments for multiple comparisons?
  - exact statistical test results, e.g., P values = x but not P values < x;
  - definition of 'center values' as median or average;
  - definition of error bars as s.d. or s.e.m.

Any descriptions too long for the figure legend should be included in the methods section and/or with the source data.

In the pink boxes below, please ensure that the answers to the following questions are reported in the manuscript itself. Every question should be answered. If the question is not relevant to your research, please write NA (non applicable).
We encourage you to include a specific subsection in the methods section for statistics, reagents, animal models and human subjects.

B- Statistics and general methods

Please fill out these boxes ê (Do not worry if you cannot see all your text once you press return)

1.a. How was the sample size chosen to ensure adequate power to detect a pre-specified effect size?	Sample size was estimated based on biological replicate experiments. We provide an example of three biological replicates of MDM2 smFISH measurements in Appendix Fig S3.
1.b. For animal studies, include a statement about sample size estimate even if no statistical methods were used.	no animal studies included
2. Describe inclusion/exclusion criteria if samples or animals were excluded from the analysis. Were the criteria pre-established?	no animal studies included
3. Were any steps taken to minimize the effects of subjective bias when allocating animals/samples to treatment (e.g. randomization procedure)? If yes, please describe.	no animal studies included
For animal studies, include a statement about randomization even if no randomization was used.	no animal studies included
4.a. Were any steps taken to minimize the effects of subjective bias during group allocation or/and when assessing results (e.g. blinding of the investigator)? If yes please describe.	no animal studies included
4.b. For animal studies, include a statement about blinding even if no blinding was done	no animal studies included
5. For every figure, are statistical tests justified as appropriate?	To estimate reproducibility and estimate sample size, we measured distribution statistics as confidence intervals using bootstrapping as described for Appendix Figure S3. For statistical comparison we used the DABEST package by Ho, 2018.
Do the data meet the assumptions of the tests (e.g., normal distribution)? Describe any methods used to assess it.	Single cell RNA FISH data does not show a normal distribution as shown in our our main Figure were we plot distributions as probability density estimates. Therefore, we used non-parametric test in the DABEST package for statistical comparison (see pt.5).

Is there an estimate of variation within each group of data?	As part of our study we calculated the single-cell heterogeneity in gene expression noise as CV2 and Fano fators. The measurements are included in the respective Figure panels.
Is the variance similar between the groups that are being statistically compared?	As indicated by the Fano factor and CV2 measurement the variance between measurement time-points and different RNA expression datasets changes, which we analyzed as part of our study.

C- Reagents

6. To show that antibodies were profiled for use in the system under study (assay and species), provide a citation, catalog number and/or clone number, supplementary information or reference to an antibody validation profile. e.g., Antibodypedia (see link list at top right), 1DegreeBio (see link list at top right).	We provide catalog numbers of antibodies and reagents in a resource table in our Materials and Methods section.
7. Identify the source of cell lines and report if they were recently authenticated (e.g., by STR profiling) and tested for mycoplasma contamination.	A549 and MCF10A cells from ATCC have been used in this study.

* for all hyperlinks, please see the table at the top right of the document

D- Animal Models

8. Report species, strain, gender, age of animals and genetic modification status where applicable. Please detail housing and husbandry conditions and the source of animals.	not applicable
9. For experiments involving live vertebrates, include a statement of compliance with ethical regulations and identify the committee(s) approving the experiments.	not applicable
10. We recommend consulting the ARRIVE guidelines (see link list at top right) (PLoS Biol. 8(6), e1000412, 2010) to ensure that other relevant aspects of animal studies are adequately reported. See author guidelines, under 'Reporting Guidelines'. See also: NIH (see link list at top right) and MRC (see link list at top right) recommendations. Please confirm compliance.	not applicable

E- Human Subjects

11. Identify the committee(s) approving the study protocol.	not applicable
12. Include a statement confirming that informed consent was obtained from all subjects and that the experiments conformed to the principles set out in the WMA Declaration of Helsinki and the Department of Health and Human Services Belmont Report.	not applicable
13. For publication of patient photos, include a statement confirming that consent to publish was obtained.	not applicable
14. Report any restrictions on the availability (and/or on the use) of human data or samples.	not applicable
15. Report the clinical trial registration number (at ClinicalTrials.gov or equivalent), where applicable.	not applicable
16. For phase II and III randomized controlled trials, please refer to the CONSORT flow diagram (see link list at top right) and submit the CONSORT checklist (see link list at top right) with your submission. See author guidelines, under 'Reporting Guidelines'. Please confirm you have submitted this list.	not applicable
17. For tumor marker prognostic studies, we recommend that you follow the REMARK reporting guidelines (see link list at top right). See author guidelines, under 'Reporting Guidelines'. Please confirm you have followed these guidelines.	not applicable

F- Data Accessibility

18: Provide a "Data Availability" section at the end of the Materials & Methods, listing the accession codes for data generated in this study and deposited in a public database (e.g. RNA-Seq data: Gene Expression Omnibus GSE39462, Proteomics data: PRIDE PXD000208 etc.) Please refer to our author guidelines for 'Data Deposition'.<br><br>Data deposition in a public repository is mandatory for:<br>a. Protein, DNA and RNA sequences<br>b. Macromolecular structures<br>c. Crystallographic data for small molecules<br>d. Functional genomics data<br>e. Proteomics and molecular interactions	We provide a "Data Availability" section in our manuscript. Quantitative data is accessible as figure source data.
19. Deposition is strongly recommended for any datasets that are central and integral to the study; please consider the journal's data policy. If no structured public repository exists for a given data type, we encourage the provision of datasets in the manuscript as a Supplementary Document (see author guidelines under 'Expanded View' or in unstructured repositories such as Dryad (see link list at top right) or Figshare (see link list at top right).	We use the expanded view features of MSB to provide EV Datasets that summarize key measurement of the study and also provide source data for all main Figures
20. Access to human clinical and genomic datasets should be provided with as few restrictions as possible while respecting ethical obligations to the patients and relevant medical and legal issues. If practically possible and compatible with the individual consent agreement used in the study, such data should be deposited in one of the major public access-controlled repositories such as dbGAP (see link list at top right) or EGA (see link list at top right).	not applicable
21. Computational models that are central and integral to a study should be shared without restrictions and provided in a machine-readable form. The relevant accession numbers or links should be provided. When possible, standardized format (SBML, CellML) should be used instead of scripts (e.g. MATLAB). Authors are strongly encouraged to follow the MIRIAM guidelines (see link list at top right) and deposit their model in a public database such as Biomodels (see link list at top right) or JWS Online (see link list at top right). If computer source code is provided with the paper, it should be deposited in a public repository or included in supplementary information.	The used software in this study has been previously published and is referenced.

G- Dual use research of concern

22. Could your study fall under dual use research restrictions? Please check biosecurity documents (see link list at top right) and list of select agents and toxins (APHIS/CDC) (see link list at top right). According to our biosecurity guidelines, provide a statement only if it could.	no
